# A highly stretchable tri-channel fiber for composite motion decoupling

**DOI:** 10.1038/s41467-026-73959-4

**Published:** 2026-06-12

**Authors:** Zhangcheng Li, Wen Wang, Kangrui Ji, Can Wang, Pan Xiong, Zeguang Du, Zhi Liang, Yu He, Bijin Xiong, Chong Hou

**Affiliations:** 1 State Key Laboratory of New Textile Materials and Advanced Processing, Research Center for Intelligent Fiber Devices and Equipment and School of Optical and Electronic Information, Huazhong University of Science and Technology, Wuhan, China; 2 Key Laboratory of Materials Chemistry for Energy Conversion and Storage of Ministry of Education, School of Chemistry and Chemical Engineering, Huazhong University of Science and Technology, Wuhan, China; 3 School of Intelligent Sports Engineering, Engineering Research Center of Sports Health Intelligent Equipment of Hubei Province, Key Laboratory of Sports Engineering of General Administration of Sports of China, Wuhan Sports University, Wuhan, China; 4 Shenzhen Huazhong University of Science and Technology Research Institute, Shenzhen, China

**Keywords:** Polymers, Electrical and electronic engineering, Mechanical properties, Sensors and biosensors

## Abstract

Fiber electronics have shown considerable potential in various applications, including electronic skin, human-machine interfaces, and intelligent sensing systems. However, stretchable fiber-based strain sensors confront fundamental challenges in concurrently achieving robust mechanical endurance, wide linear response range, and effective composite motions decoupling under complex deformation conditions. Here we present a highly stretchable tri-channel fiber featuring concentric and double-helical microchannels integrated with gallium-based liquid metal, constructing a dual-strain fiber sensor capable of decoupling composite motions involving both elongation and torsional deformations. The helical architecture promotes a three-dimensional orientation of polymer chains, thereby effectively enhancing both the stretchability and cyclic durability of the sensor. Owing to the specific configuration within the fiber, the sensor exhibits a highly linear response to tensile strain, along with bidirectional torsional strain sensing across a wide operational range. Furthermore, by synergistically integrating geometric deformation with hybrid resistive-capacitive sensing mechanisms, the sensor demonstrates the ability to simultaneously monitor and decouple stretching and twisting composite motion behaviors. This strategy enables the precise characterization of object motion and deformation states, offering valuable prospects for real-time health monitoring and motion tracking applications.

## Abstract (web summary)

Regulated helical orientation of polymer chains proved to greatly enhance fiber mechanics, and the highly stretchable fibers were capable of simultaneously sensing and decoupling tensile and torsional strains via a designed tri-channel structure

## Introduction

Recent advances in fiber electronics have facilitated the integration of functional materials into fibers with tailored geometries and architectures^1–5^, thereby unlocking a broad spectrum of functionalities, including sensing^6,7^, display^8,9^, thermal management^10,11^, and smart textiles^12,13^. Particularly in the context of the rapid growth of robotics and wearable technologies, monitoring and decoupling composite mechanical motions—such as elongation, bending, and torsion—have emerged as critical capabilities for executing complex tasks in dynamic operational environments. This imposes stringent requirements on the flexibility, durability, and strain-response performance of the devices^14–17^. Notably, stretchable fiber-based devices exhibit exceptional promise due to their intrinsically one-dimensional structure and superior shape conformability, rendering them well-suited for diverse technological fields, such as deformation detection^18,19^, human-machine interaction^20,21^, and soft robotics^22,23^. Despite progress in performance and functionality, existing stretchable fiber strain sensors still face pressing challenges in concurrently achieving mechanical compliance and electrical stability under sustained strain^24,25^. Current stretchable fiber strain sensors frequently exhibit deficiencies in response linearity, sensing range, and mechanical endurance^26^. These limitations compromise accuracy under extreme strain conditions and long-term reliability, thereby restricting their capacity to simultaneously monitor and decouple composite motion modes in practical scenarios^27^.

Effective approaches for decoupling composite motions under extreme strain remain limited, primarily owing to constraints, such as inadequate material deformability and signal interference among varied sensing mechanisms^28^. The mechanical and sensing performance of fiber strain sensors is largely governed by both material properties and structural configurations^29^. On the one hand, intrinsically elastic polymers or hydrogels can be incorporated with functional nanomaterials to establish signal transduction pathways and strain-responsive mechanisms within stretchable fiber architectures^30,31^. Modifying the physicochemical properties of the elastic matrix via molecular structure designs and orientation of polymer chains can effectively improve both the stretchability and sensing stability of fiber sensors^32,33^. On the other hand, engineered device geometries utilizing wavy, island-bridge, or helical configurations facilitate improved stress distribution and localized strain mitigation, thereby reinforcing overall mechanical resilience^34,35^. The incorporation of such specific structures also introduces new sensing capabilities, enabling the sensor to respond to varying strain modalities by altering geometric parameters. Nevertheless, reconciling the balance between mechanical robustness and multimodal strain sensing capability remains a persistent challenge^36^. Despite the proliferation of research into multi-modal fibers, the majority of existing stretchable strain sensors prioritize performance optimization under isolated deformation modes^37^. This limitation stems not from a deficiency in fundamental sensing mechanisms but rather from the inherent challenge of decoupling independent signals from either monolithic materials or simple conductive pathways subjected to complex strain coupling. Consequently, a critical capability gap remains regarding the accurate, real-time monitoring of dynamically superimposed strain states.

Here, we demonstrate a highly stretchable tri-channel fiber incorporating concentric and double-helical microchannel architectures, integrated with gallium-based liquid metal to construct a dual-strain fiber sensor capable of decoupling composite motions involving both tensile and torsional deformations. The formation of helical microchannels induces a spatially ordered, three-dimensional helical orientation of the polymer chains within the fiber. This specific alignment significantly enhances the intermolecular interactions and improves energy dissipation under mechanical deformation^38,39^, thereby endowing the fiber with an elongation at break exceeding 1900%. Concurrently, the sensor demonstrates exceptional durability, sustaining 75,000 loading cycles under 200% tensile strain and 425,000 cycles under ±1000 rad/m torsional deformation. By leveraging the architecture of multiple embedded microchannels, the sensor demonstrates a highly linear response to tensile strain and bidirectional sensitivity to torsional strain. Through the integration of hybrid resistive and capacitive sensing mechanisms within the liquid metal-filled microchannels, simultaneous monitoring and discrimination of superimposed tensile and torsional strain states are achieved. A real-time visualization system based on the dual-strain fiber sensor was developed, capable of simultaneously quantifying both stretching magnitude and twisting angle. This dual-strain fiber sensor exhibits significant potential for integration into intelligent sensing platforms, offering broad prospects for real-time health monitoring, high-resolution motion tracking, and complex deformation analysis.

## Results

### Tri-channel fiber design and fabrication

**Fig. 1:**
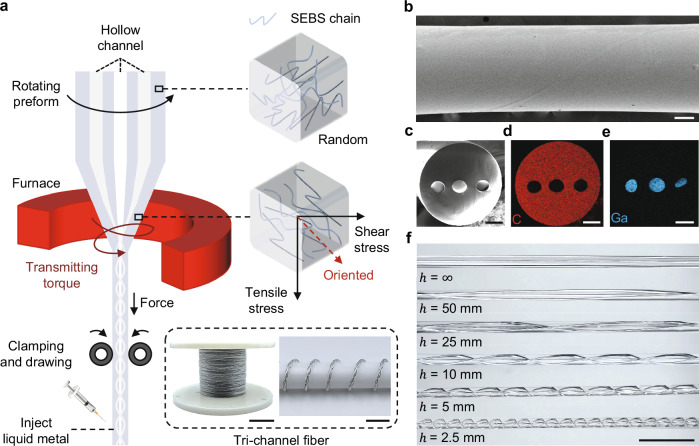
Fabrication and morphology of the tri-channel fiber. **a** Schematic illustration of the fabrication process of the tri-channel fiber and the construction of conductive liquid metal-filled microchannels. The inset shows the fabricated 50 m-long tri-channel fiber and its excellent flexibility (left scale bar, 5 cm; right scale bar, 5 mm). **b** Scanning electron microscope (SEM) image of the side surface of the fiber (scale bar, 200 μm). **c–e** SEM image of the fiber cross-section integrated with liquid metal, corresponding energy-dispersive spectroscopy (EDS) elemental mapping of C and Ga, representing poly(styrene-b-(ethylene-co-butylene)-b-styrene) (SEBS) and liquid metal, respectively (scale bar, 200 μm). The micrographs shown in (**b–e)** are representative of three independent experiments that yielded similar results. **f** Photograph of tri-channel fibers with different $h$ (scale bar, 5 mm).

The fabrication of the tri-channel fiber is achieved using a preform-to-fiber thermal drawing technique, in which a thermoplastic polymer is initially processed into a macroscopic preform featuring a precisely engineered cross-sectional architecture (Supplementary Fig. 1a–h). This preform is subsequently heated above its glass transition temperature and continuously drawn into a microscale fiber while preserving geometric fidelity to the original architecture of the preform^40^ (Supplementary Fig. 2a–d). In this process, the thermoplastic elastomer poly(styrene-b-(ethylene-co-butylene)-b-styrene) (SEBS) is employed to fabricate cylindrical preforms containing three straight channels. One of these channels is positioned concentrically within the preform, while the other two are symmetrically placed on either side of the central channel. During thermal drawing, rotation of the preform results in the concentric channel remaining straight, whereas the two eccentric channels undergo helical coiling due to torque transmission, thus forming a double-helical microchannel architecture within the stretchable fiber (Fig. 1a). Stable fiber formation is critically dependent on the rheological properties and thermal stability of SEBS (Supplementary Fig. 3a–c). As a consequence of the Weissenberg effect and shear-thinning behavior of viscoelastic materials (Supplementary Fig. 3b), when the drawing and rotation speeds exceed a certain threshold, material within the preform flows inward from the periphery towards the center^41,42^. This inward migration can lead to deformation and eventual closure of the microchannels (Supplementary Fig. 4a-c). Hence, precise regulation of both the drawing and rotational speeds is essential to maintain the structural integrity and uniformity of the microchannels (see Supplementary Note 1 for further details). During thermal drawing, the temperature surpasses the glass transition temperature of SEBS, transforming it into the viscous flow regime with the enhanced thermal motion and disentanglement of polymer chains^43^. Consequently, the SEBS chains become aligned with the direction of the applied axial force^44^. In addition, rotational motion of the preform imposes shear forces, which induce SEBS chains orientation and extension at an angle relative to the fiber axis. By adjusting both the drawing and rotation speeds, the tilt angle of chain alignment can be effectively regulated.

Through thermal drawing, the tri-channel fiber exhibiting exceptional flexibility and stretchability can be fabricated in continuous lengths. Subsequently, a highly conductive and fluid gallium-based liquid metal was infused into the microchannels to establish stretchable conductive pathways throughout the fiber. As shown in Fig. 1b–e, the tri-channel fiber features a smooth cylindrical outer surface with a diameter of approximately 1 mm, containing three parallel microchannels, each approximately 160 μm in diameter and filled with liquid metal. The longitudinal cross-section more clearly reveals the internal structural configuration, comprising one concentric and two helical microchannels (Supplementary Fig. 4d). By adjusting the process parameters during thermal drawing, fibers with different helical periods ($h$) can be fabricated, as illustrated in Fig. 1f.

### Regulation of polymer chain orientation enhances fiber mechanical properties

**Fig. 2:**
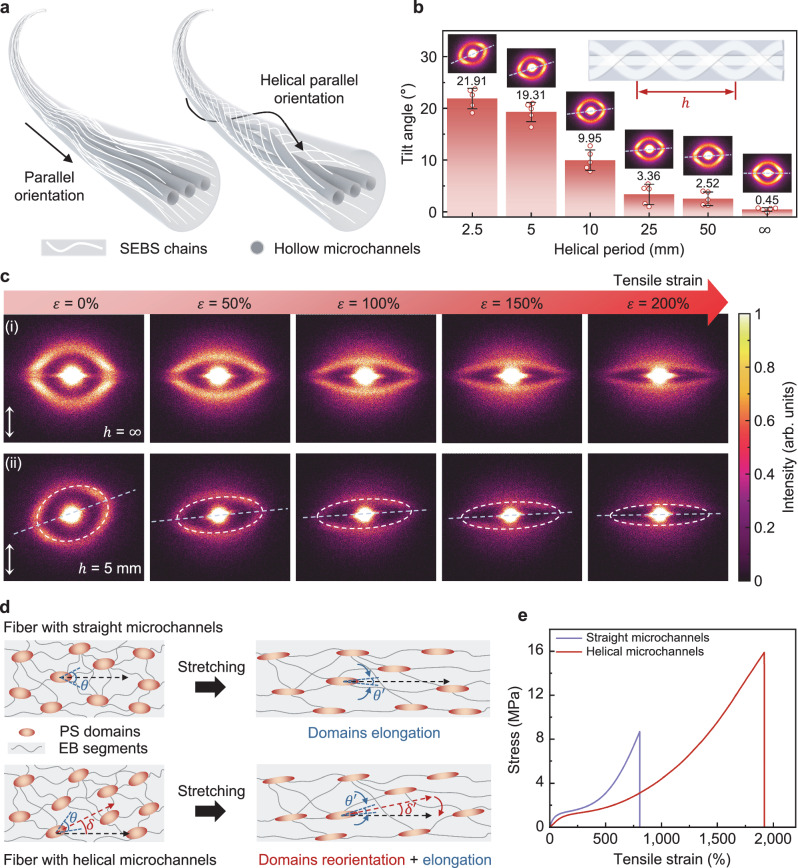
Regulation of polymer chain orientation enhances fiber mechanical properties. **a** Schematic diagram of polymer chain orientation in fibers with straight and helical microchannels (SEBS, poly(styrene-b-(ethylene-co-butylene)-b-styrene)). **b** Polymer chain tilt angles in tri-channel fibers with different $h$. Values above the error bars represent the mean, and error bars indicate the standard deviation (*n* = 5 independent samples). The inset shows the corresponding 2D SAXS patterns and a schematic of a fiber with a helical period of $h$. **c** 2D SAXS patterns of the fiber with straight microchannels ($h$ = $\infty$, i) and the fiber with helical microchannels ($h$ = 5 mm, ii) under different tensile strains. **d** Schematic diagram illustrating the evolution of PS domains and polymer chain orientation in fibers with straight and helical microchannels during stretching (PS, polystyrene; EB, ethylene-butylene). **e** Stress-strain curves for fibers with straight ($h$ = $\infty$, purple) and helical ($h$ = 5 mm, red) microchannels. Source data are provided as a Source Data file.

To elucidate the impact of preform rotation during thermal drawing on polymer chains orientation and mechanical properties of the tri-channel fiber, a systematic series of material characterization and mechanical testing protocols was conducted. Figure 2a depicts polymer chains orientation in two distinct types of tri-channel fibers fabricated via thermal drawing under either static or rotational preform conditions. In the absence of preform rotation, the microchannels remain in a straight configuration (corresponding to $h$ = $\infty$), and SEBS chains are predominantly aligned along the axial direction of the fiber. In contrast, when the preform undergoes rotation during the thermal drawing, the superimposed tensile and shear stresses induce SEBS chains elongation and alignment into a helical orientation along the fiber’s axial direction, resembling a series of intertwined springs. By adjusting fabrication parameters, the $h$ of the microchannels can be controlled, thereby modulating the periodicity of SEBS chains orientation along the fiber. The SEBS chains consist of alternating hard polystyrene (PS) segments and soft ethylene-butylene (EB) copolymer segments. Due to the thermodynamically incompatible of PS and EB, microphase-separated domains are formed within SEBS^43^. Small- and wide-angle X-ray scattering (SAXS/WAXS) measurements were conducted to analyze microphase-separated structure and the orientation of polymer chains in the original preform and tri-channel fibers with varying $h$. In intact fibers with helical microchannels, helically aligned polymer chains yield a symmetric, elliptical SAXS pattern arising from the superposition of scattering signals. To unambiguously resolve the polymer chain orientation relative to the fiber axis, the fibers were longitudinally sectioned into semi-cylindrical specimens prior to SAXS characterization (see Supplementary Note 2 and Supplementary Fig. 5a–d for details). Compared to the preform, the 2D SAXS pattern of the fiber with straight microchannels shows a transition from a centrally symmetrical circular scattering profile to an elliptical ring (Supplementary Fig. 6a, b), indicating the transformation of PS domains from spherical to ellipsoidal shapes (Supplementary Fig. 7a) and the axial alignment of polymer chains after thermal drawing. In fibers featuring helical microchannels, the 2D SAXS patterns reveal that as the $h$ decreases, the angle between the polymer chain orientation and the fiber axis increases, reflecting an enhanced degree of helical alignment (Fig. 2b). The polymer chain tilt angles derived from the SAXS patterns closely match the helix angles of corresponding microchannels, conforming that preform rotation during thermal drawing simultaneously regulates both the macroscopic fiber architecture and the microscopic domains and polymer chains orientation.

SAXS measurements were performed on fibers featuring two distinct microchannel architectures under varying tensile strains ($\varepsilon$) to elucidate alterations in PS domains morphology and polymer chain orientation during fiber elongation. As illustrated in Fig. 2c(i), the 2D SAXS patterns of the fiber with straight microchannels display a progressively flattened elliptical scattering ring with increasing tensile strain, indicating gradual alignment of the PS domains and polymer chains along the stretching direction. In contrast, the fiber with helical microchannels ($h$ = 5 mm) exhibits a tilted elliptical pattern in the relaxed state, as shown in Fig. 2c(ii). As tensile strain increases, the elliptical scattering ring becomes more flattened, with its major axis gradually aligning horizontally. This indicates that the initially tilted polymer chains reorient towards the axial direction as the fiber is stretched. Furthermore, a comparison of the 2D SAXS patterns under identical tensile strain conditions reveals that the scattering ring of the fiber with straight microchannels is more flattened than that of the helical counterpart, implying that its internal polymer chains experience a greater degree of alignment and stretching.

As shown in Fig. 2d, the two types of fibers with distinct architectures exhibit different behaviors in terms of PS domains and polymer chains variation during the stretching process. In the relaxed state, ellipsoidal PS domains within the fiber with straight microchannels are distributed with a large orientation angle ($\theta$) along the fiber’s axial direction. Upon stretching, both PS domains and EB segments undergo elongation and axial alignment, resulting in an enhanced orientation and flattening of the domains, which subsequently cause a reduction in $\theta$ (Supplementary Fig. 7b). In contrast, in the fiber with helical microchannels, the PS domains are initially inclined relative to the fiber axis, forming an inclination angle ($\delta$). As tensile strain increases, in addition to undergoing similar elongation and flattening as in the fiber with straight microchannels, the PS domains progressively reorient towards the axial direction (Supplementary Fig. 7c). Consequently, $\delta$ exhibits a progressive decline as the helical polymer chains align axially, analogous to the straightening of a series of springs (Supplementary Note 2, Supplementary Fig. 8a, b). The divergent evolution patterns in PS domains and polymer chains orientation under tensile strain contribute to notable disparities in mechanical performance between the two fiber types. As depicted in Fig. 2e, the fiber with helical microchannels exhibits a substantial enhancement in both elongation at break (from 805% to 1918%) and breaking strength (from 8.68 MPa to 15.91 MPa), relative to its counterpart with straight microchannel. This enhanced mechanical behavior is primarily attributed to the specific chain orientation regulated by rotational thermal drawing, which strengthens the intermolecular interactions and the material’s capacity for energy dissipation during deformation^38^ (Supplementary Fig. 9, and see Supplementary Note 3 for details). Tri-channel fibers with helical periods varying from 2.5 to 50 mm consistently exhibit enhanced fracture elongation and breaking strength compared to their counterparts with *h* = ∞ (Supplementary Fig. 10a). During initial strain stage, the fiber with straight microchannels demonstrates a higher yield stress, attributable to the initial polymer chains alignment parallel to the loading axis. By contrast, the helically oriented chains in the fiber with helical microchannels alleviate the stress required to initial deformation, resulting in a lower initial modulus. In addition, the effect of liquid metal infusion into the microchannels on the mechanical properties of the fibers was examined (Supplementary Fig. 10b). The results indicate that the high fluidity of the liquid metal allows it to accommodate fiber deformation through flow within the microchannels, thus exerting only a small influence on the intrinsic mechanical properties of the fiber matrix. The mechanical hysteresis behavior of the tri-channel fiber was further investigated across various tensile strain levels and under cyclic loading, demonstrating excellent elastic recovery and durability (Supplementary Figs. 10–12).

### Simultaneous monitoring and discrimination of tensile and torsional strain

**Fig. 3:**
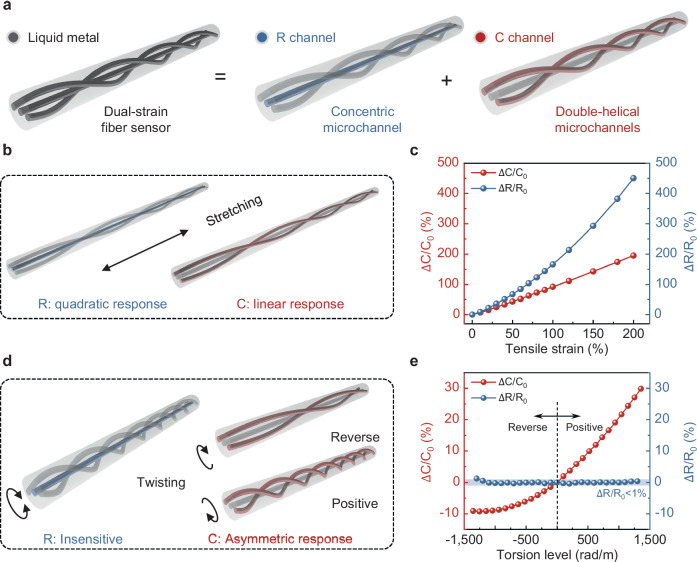
Working mechanism of dual-strain fiber sensor for simultaneous monitoring and discrimination of tensile and torsional strains. **a** Schematic diagram of the electrical signal outputs from the fiber sensor. The concentric microchannel outputs resistive signals, while the double-helical microchannels output capacitive signals. **b**, **c** Schematic diagrams and corresponding response curves showing the variation in resistance and capacitance of the fiber sensor ($h$=5 mm) under tensile strain. **d**, **e** Schematic diagrams and corresponding response curves showing the variation in resistance and capacitance of the fiber sensor ($h$=5 mm) under torsional strain. Source data are provided as a Source Data file.

The structural uniqueness of the tri-channel fiber imparts distinct architectural responses in its internal concentric and helical microchannels under applied tensile and torsional strains. These structural variations enable the tri-channel fiber to integrate with liquid metal (Supplementary Fig. 13), thereby constructing a dual-strain fiber sensor capable of simultaneously monitoring and discriminating tensile and torsional strains, ultimately enabling the decoupling of composite motions. For the subsequent analysis, a dual-strain fiber sensor with $h$=5 mm is utilized as a representative example. As depicted in Fig. 3a, the concentric and double-helical conductive microchannels within the sensor serve as signal pathways for resistive and capacitive outputs, respectively. Figures 3b, c show that resistance and capacitance exhibit different response characteristics under pure tensile strain. C_0_ and R_0_ denote the initial capacitance and resistance of the fiber in its unstrained state, respectively. ΔC and ΔR represent the absolute changes in capacitance and resistance from their initial values following the application of strains. The corresponding relative changes are defined as ΔC/C_0_ and ΔR/R_0_. The ΔR/R_0_ exhibits a quadratic dependence on tensile strain, whereas the ΔC/C_0_ displays a linear relationship (see Supplementary Note 4 for details). Although the gauge factor (GF) of the capacitance response is lower than that of the resistance response, the capacitive response exhibits superior linearity over an extensive range. This linear characteristic is sustained until severe structural deformations of microchannels disrupt liquid metal conductive pathways under extreme mechanical strain. Such high linearity is crucial for improving the accuracy in high-strain regimes. As shown in Fig. 3d, e, the resistance remains largely unaffected by pure torsional strain (ΔR/R_0_ < 1% across a broad torsional strain range from −1000 to 1000 rad/m), owing to the deformation of the concentric microchannel is negligible under axial torsion (Supplementary Fig. 14a–c). Notably, when torsional strain exceeds a critical threshold, excessive torsion may induce additional tensile deformation alongside the primary torsional deformation. This deformation alters the geometry of the concentric resistance channel, resulting in the ΔR/R_0_ being unable to remain within 1%. As evidenced in Fig. 3e, the resistance signal exhibits a slight increase under high magnitudes of reverse torsional strain. More critically, this can lead to snarling (Supplementary Fig. 15a–c), which severely compromises the accurate perception of strains. In contrast, the inherent asymmetry of the double-helical configuration induces dimensional and axial $h$ variations in both microchannels under pure torsional strain, leading to measurable changes in capacitance. Furthermore, the effects of positive and reverse torsion on the helical microchannels structure are oppositional, enabling the determination of torsion direction based on the positive and negative relative changes in capacitive signal (see Supplementary Note 5 and Supplementary Fig. 16a–d for details).

When tensile and torsional strains are applied concurrently, the resistive signal responds exclusively to tensile strain while remaining insensitive to torsion. Conversely, variations in the capacitive signal arise from the synergistic influence of both strain types. Consequently, decoupling these combined loading conditions is unachievable by monitoring either the resistive or capacitive signal in isolation. This necessitates a system-level integration strategy in which multiple microchannels are used to incorporate distinct sensing mechanisms within a single fiber, thereby enabling strain separation via analytical modeling. Specifically, this involves a sequential analytical approach: first quantifying tensile strain via the resistive signal, and subsequently utilizing this value alongside the capacitive response to decouple and resolve the torsional strain (see Supplementary Note 6 for details). Thus, the synergistic utilization of both electrical sensing mechanisms is essential to not only distinguish individual strains but also effectively decouple and monitor them under complex combined loading conditions.

### Electromechanical and composite motions decoupling performance of dual-strain fiber sensor

**Fig. 4:**
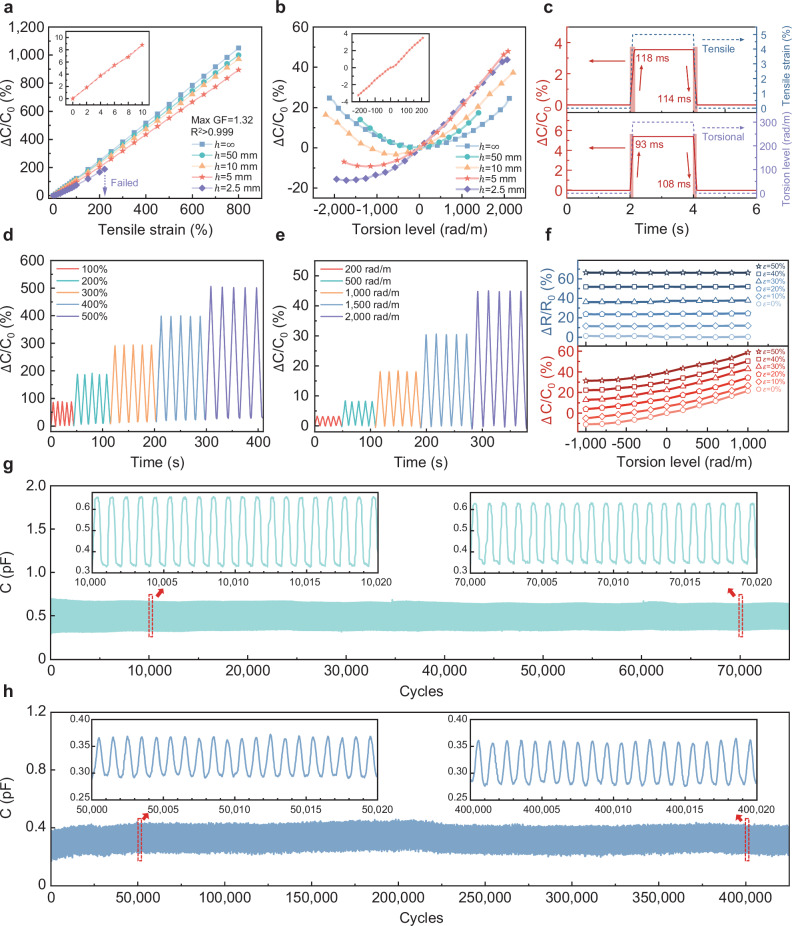
Electromechanical performance of dual-strain fiber sensor. **a** Capacitive response of sensors with different $h$ under tensile strain. The inset shows the response at small tensile strains (from 0% to 10%), with the same units as in the main plot. **b** Capacitive response of sensors with different $h$ under torsional strain. The inset shows the response at small torsional strains (from −200 rad/m to +200 rad/m), with the same units as in the main plot. **c** Response and recovery times of the sensor under 5% tensile and 300 rad/m torsional strains. **d** Capacitive response of the sensor under varying tensile strains (100–500%). **e** Capacitive response of the sensor under varying torsional strains (200–2000 rad/m). **f** Resistive and capacitive response of the sensor measured under torsional strain at various fixed tensile strains. **g** Capacitive response of the sensor under cyclic 200% tensile strain over 75,000 cycles. The inset shows the 10,000–10,020 cycles and 70,000–70,020 cycles. **h** Capacitive response of the sensor under cyclic $\pm$1000 rad/m torsional strain over 425,000 cycles. The inset shows the 50,000–50,020 cycles and 400,000–400,020 cycles. Source data are provided as a Source Data file.

The dual-strain fiber sensor demonstrates distinctive electromechanical performance, attributed to the helical orientation of the polymer chains and the multiple microchannel structure within the tri-channel fiber. Both the resistive (Supplementary Fig. 17a–d) and capacitive responses of the sensor were comprehensively characterized. In the case of a single deformation, strain can be accurately sensed solely through the capacitive response. As shown in Fig. 4a and Supplementary Fig. 18a–d, fiber sensors with different $h$ exhibit highly linear capacitive response across a tensile strain range of 0% to 800%, with coefficients of determination exceeding 0.999. Among these, the sensor with $h$ = $\infty$ achieves a maximum GF of 1.32. As $h$ decreases, the GF gradually declines. For $h$ = 2.5 mm, the electrical capacitive signal fails once the tensile strain surpasses 220%, even though the fiber remains physically intact. This is attributed to the elongation and cross-sectional contraction of the microchannels during stretching, which makes those with smaller $h$ more susceptible to collapse, thereby disrupting liquid metal continuity and leading to premature signal failure. Figure 4b presents the capacitive response of sensors with various $h$ under the torsional strain range from -2000 to 2000 rad/m. When $h$ = $\infty$, the sensor exhibits a symmetric response to both positive and reverse torsion. For sensors with $h$ ≠ $\infty$, the relative capacitive responses under reverse torsion initially decrease before subsequently increasing. As $h$ decreases, the sensitivity of the capacitive response increases, reaching a maximum of 0.022, whereas the torsion level corresponding to the minimum response value gradually decreases (Supplementary Fig. 19a–f). This minimum point corresponds to the state where the helical microchannels are twisted in reverse to such an extent that they become fully straightened. Consequently, the sensor with a smaller $h$ exhibits a broader sensing range of torsional strain.

To comprehensively optimize the electromechanical performance of the dual-strain fiber sensor under both tensile and torsional strains, the sensor with $h$ = 5 mm was selected for further evaluation and application. The sensor demonstrates a rapid dynamic response, with tensile/recovery times of 118 ms/114 ms and torsion/recovery times of 93 ms/108 ms, respectively (Fig. 4c). To assess its operational reliability, systematic testing was conducted to evaluate response stability under various strain conditions. Owing to the high stretchability and resilience of the tri-channel fiber, the sensor exhibits stable and distinguishable capacitive response under repeated tensile strains of 100%, 200%, 300%, 400%, and 500%, as well as repeated torsional strains of 200, 500, 1000, 1500, and 2000 rad/m (Fig. 4d, e). Furthermore, the electrical hysteresis behavior of the sensor was investigated (Supplementary Fig. 20a, b). Following multiple strain cycles, the electrical hysteresis curves for both tension and torsion exhibited substantial overlap between the loading and unloading phases. This overlap signifies excellent signal consistency and repeatability, confirming negligible electrical hysteresis during dynamic measurements.

To assess the potential of the dual-strain fiber sensor for complex motion monitoring applications, both resistive and capacitive responses were evaluated under combined tensile and torsional loading conditions. To ensure decoupling stability and long-term reliability under combined strain, the effective operational range of the sensor was confined to tensile strains ≤ 50% and torsional strains within ±1000 rad/m. As shown in Fig. 4f, the two sensing modalities demonstrate fundamentally distinct response characteristics under hybrid mechanical deformation. Specifically, the resistive response remains insensitive to torsional strain, regardless of the tensile strain level within the 0–50% range (Fig. 4f, top, and Supplementary Fig. 21a). In contrast, the capacitive response pattern to torsional strain remains consistent and predictable across different levels of applied tensile strain (Fig. 4f, bottom). Furthermore, under fixed torsional strain, the capacitive response exhibits a linear dependence on increasing tensile strain (Supplementary Fig. 21b). These differential response characteristics enable effective monitoring and decoupling of composite mechanical states through simultaneous acquisition and analysis of both signal outputs.

Remarkably, the sensor maintains a consistent capacitive response with over 90% signal retention after 75,000 cycles under 200% pure tensile strain and 425,000 cycles under $\pm$1,000 rad/m pure torsional strain, confirms its exceptional durability (Fig. 4g, h, and Supplementary Fig. 11). Attributable to the rapid elastic recovery of the elastomeric matrix and the inherent fluidity of the liquid metal, the sensor exhibits high signal consistency and real-time responsiveness across various loading rates (Supplementary Fig. 22a, b), thereby satisfying the demands of most quasi-static and low-frequency dynamic oscillatory sensing scenarios. A comprehensive comparative of key performance metrics between the dual-strain fiber sensor and previously reported strain sensors as shown in Supplementary Fig. 23 and Supplementary Table 1, presenting the superior performance of our fiber strain sensor across evaluated parameters.

**Fig. 5:**
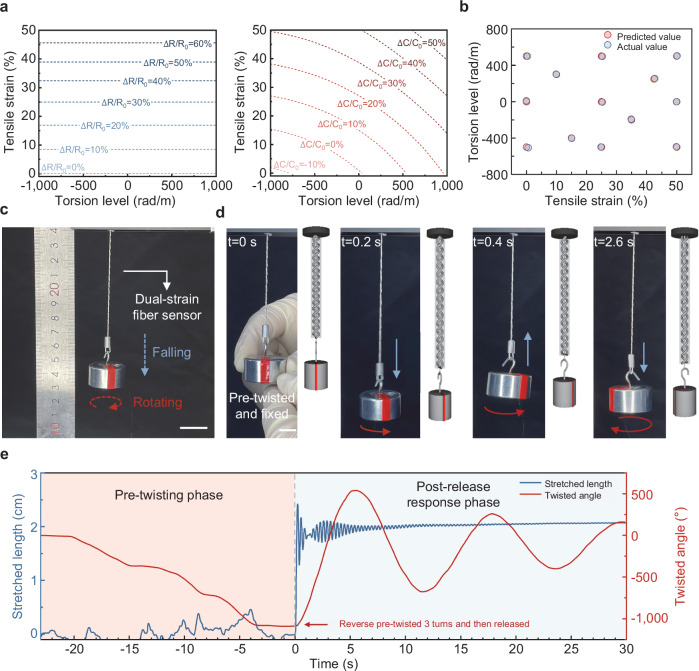
Composite motions decoupling performance of the dual-strain fiber sensor. **a** Numerical simulation of the dual electrical signals response under coupled tensile and torsional strain. **b** Prediction strain states of the sensor under different combinations of tensile strains and torsion levels. **c** Experimental platform for simultaneous monitoring and discrimination of tensile and torsional strains (scale bar, 2 cm). **d** Digital twin model reflecting the real-time motion states of the objects (scale bar, 1 cm). **e** Real-time response curves of the actual stretched length and twisted angle of the objects. Source data are provided as a Source Data file.

Calibration data quantifying the relative variations in resistance and capacitance under predefined tensile-torsional strain combinations (Fig. 4f) were employed to construct a numerical model. This model establishes the quantitative relationships between the sensor’s electrical responses and the two strain modes (Fig. 5a). Leveraging this numerical model and capitalizing on the exclusive sensitivity of the resistive signal to tensile strain, the tensile strain can be directly and first quantified from the relative resistance change (Fig. 5a, left). Subsequently, the torsional strain can be decoupled by incorporating the quantified tensile strain together with the relative capacitive variation (Fig. 5a, right). The model’s fidelity was validated under preset composite strain conditions to ensure reliability during dynamic monitoring. As shown in Fig. 5b and Supplementary Fig. 24, the system accurately predicts both strain states, with calculated values closely tracking the predefined parameters.

An experimental platform (Fig. 5c) was developed to evaluate the sensor’s real-time monitoring and decoupling capabilities under hybrid mechanical motions. Initially, a weight was suspended from the lower end of the dual-strain fiber sensor, maintaining the sensor in its original length state and pre-twisted. While the mass of the suspended weight governs the tensile response profile, the torsional response is determined by the interplay between the object’s moment of inertia and the initial pre-twist angle. Upon release, the suspended mass induced simultaneous axial elongation and torsional recovery, generating oscillatory deformation cycles. As shown in Fig. 5d, e, the deformation states of the dual-strain fiber sensor were tracked and decoupled in real-time through the combined analysis of capacitive and resistive response, enabling the reconstruction of motion states through digital modeling and interactive visualization (Supplementary Movie 1). Decoupling analysis revealed distinct temporal evolution behaviors for the tensile and torsional components during dynamic deformation. Under the influence of gravitational loading and fiber elastic restoration forces, tensile deformation exhibited higher-frequency, low-amplitude oscillations, while torsional deformation demonstrated lower-frequency oscillations with prolonged periodic. Furthermore, decoupling results obtained under varying load masses and pre-torsion turns are presented in Supplementary Figs. 25 and 26. These results highlight the sensor’s remarkable ability to monitor and decouple complex mechanical deformation states, providing valuable strategies and methodologies for applications demanding precise decoupling of composite motions.

## Discussion

In summary, we have demonstrated a highly stretchable tri-channel fiber with concentric and double-helical microchannels, integrated with liquid metal to construct a dual-strain fiber sensor capable of decoupling composite elongation and torsional motions. The helical structure aligns the polymer chains along the fiber axis, enabling reliable electromechanical performance under large strains and stability in dynamic sensing scenarios. By synergistically combining resistive and capacitive sensing mechanisms through the multiple microchannels architecture, the sensor achieves concurrent dual-strain monitoring with high linearity in tensile sensing and bidirectional torsional detection capability. A digital twin visualization system has been developed for real-time synchronous monitoring and decoupling of stretch-twist deformations, demonstrating the sensor’s practical applicability under complex motion conditions. This dual-strain fiber sensor resolves the persistent challenge of real-time decoupling for superimposed strains, holding significant promise for advanced intelligent systems, particularly in applications requiring real-time health monitoring, motion analysis, and high-precision motion capture.

## Methods

### Fabrication of the tri-channel fiber and the dual-strain fiber sensor

#### Preform Fabrication

SEBS pellets (Kraton, G1657) were dried in a vacuum oven (Shanghai Jingqi Instrument Co., Ltd., DZF-6020) at 60 °C for 24 hours to remove residual moisture. The dried pellets were then loaded into a custom-designed mold with semicircular grooves and hot-pressed using a hydraulic press (Hefei Kejing Materials Technology Co., Ltd., YLJ-HP300) at 130 °C and 5 MPa for 15 min, yielding two semicircular blocks, each 200 mm in length and 20 mm in diameter. Three semicircular grooves, each with a depth of 2 mm, a diameter of 4 mm, and a length of 200 mm were milled into the flat surfaces of the blocks using a carving machine (JingYan Instruments & Technology Co., Ltd., CNC4030). One groove was located concentrically, while the remaining two were symmetrically positioned 2 mm apart on either side of the concentric groove. The two grooved semicircular blocks were then aligned and assembled to form a cylinder preform containing three hollow circular channels, each with a diameter of 4 mm. Stainless-steel rods, each with a diameter of 4 mm, were inserted into each channel. This preform assembly was thermally consolidated in a tubular furnace (Hefei Kejing Materials Technology Co., Ltd., OTF-1200X) at 113 °C and 0.05 MPa for 30 min. Following consolidation, the stainless-steel rods were removed, resulting in the formation of a multicore preform containing three longitudinal hollow channels, prepared for subsequent thermal drawing.

#### Thermal drawing of the tri-channel fiber

Prior to thermal drawing, a 1 mm diameter radial hole was drilled 10 mm from the base of the preform. A stainless-steel wire was threaded through this hole and used to suspend a 100 g weight, providing tension during the initial drawing process. The upper end of the preform was attached to a servo motor and lifted into the heating furnace of a thermal drawing tower (Shanghai YuZhi Technology Co., Ltd.). The furnace was set to three distinct temperature zones: 185 °C (top), 80 °C (middle), and 30 °C (bottom). Once the preform head softened and dropped, the resulting fiber was guided into the traction system. The final diameter of the fiber was controlled by adjusting the feeding and drawing speeds, while the $h$ of the internal microchannels was determined by the rotation speed of the servo motor. For instance, when the feeding speed was set to 0.8 mm/min, the drawing speed to 300 mm/min, and the rotation speed to 60 rpm, a tri-channel fiber with a uniform outer diameter of approximately 1 mm and an internal helical microchannels period of 5 mm was successfully fabricated.

#### Assembly of the dual-strain fiber sensor

Gallium-based liquid metal (68.5 wt% Ga, 21.5 wt% In, and 10 wt% Sn, Sino Santech Materials Technology Co., Ltd.) was injected into the three microchannels of the fabricated fibers using a syringe. Copper wires with a diameter of 80 μm were inserted at both ends of the concentric microchannel and at one end of each of the double-helical microchannels to establish the resistive and capacitive signal pathways within the dual-strain fiber sensor. Finally, both ends of the sensor were sealed with epoxy resin to prevent leakage of the liquid metal.

### Materials characterization

The rheological properties of SEBS were measured using a rheometer (Haake, Mars40) under steady shear conditions. During testing, polymer plates (thickness: 1 mm) were heated at a rate of 3 °C/min, with the angular frequency maintained at 1 rad/s. Thermal stability was evaluated via thermogravimetric analysis (Netzsch, STA 2500 Regulus). Morphological characterization of the tri-channel fiber was performed using SEM (Zeiss, Gemini 300) at an acceleration voltage of 3 kV with an SE2 detector. Fiber samples for SEM were prepared by immersing them in liquid nitrogen for 1 min, followed by immediate cutting. The cut surfaces were sputter-coated with a 10 nm gold layer prior to characterization. Elemental analysis via EDS and mapping was conducted at an accelerating voltage of 10 kV. The evolution of microphase separation structure and polymer chains within the tri-channel fiber was investigated in situ using 2D SAXS and WAXS at the GeniX3D Cu beamline (Xenocs, Xeuss 3.0 UHR), operating at 50 kV and 600 μA with a wavelength λ = 1.54 Å. To examine the effect of preform rotation on the microphase separation structure and polymer chain orientation during thermal drawing, fiber samples were sectioned along the axial direction and placed on the sample stage. The sample-to-detector distances for SAXS and WAXS were set to 1,500 and 60 mm, respectively. Exposure time was set to 300 s. Data processing to extract the integrated diffracted intensity as a function of scattering vector ($q$) and azimuthal angle ($\varPsi$) was carried out using the Fit2D software. Detailed SAXS and WAXS data collection and processing parameters are provided in Supplementary Table 2. All photographic images presented in this article were captured using an original camera.

### Mechanical and electrical characterization

Mechanical tensile testing was performed using an electronic universal testing machine (MTS Systems (China) Co., Ltd., C45.105EY). For tensile electromechanical response measurement, the elongation of the fiber strain sensor was controlled using an electrically driven linear translation stage (Wuhan Red Star Yang Technology Co., Ltd., EPSA300G). Torsional response measurements were performed by adjusting the torsion level via a rotary motor coupled with a hollow rotational platform. Resistance and capacitance were recorded using a multifunctional electrical testing instrument (LZ-01ARC, Hangzhou LinkZill Technology Co., Ltd., China). Data processing and plotting were performed using Origin. Data acquisition was carried out using computer-controlled software developed in LabVIEW. The raw electrical output data were further processed in MATLAB, and the corresponding stretching length and twisting angle were visualized in real-time via a digital twin model and dynamic response curve.

### Reporting summary

Further information on research design is available in the Nature Portfolio Reporting Summary linked to this article.

## Supplementary information

Supplementary Information

Description of Additional Supplementary File

Supplementary Movie 1

Reporting Summary

Transparent Peer Review file

## Source data

Source Data

## Data Availability

All data generated in this study have been deposited in the Figshare repository under accession code [10.6084/m9.figshare.31898974]. All data supporting the findings of this study are available in the main manuscript, the Supplementary Information and the Source Data file. Source data are provided with this paper.

## Code availability

No custom code was generated in this study. All data processing and analyzes were carried out using standard commercial and open‑source software without any bespoke code.
