# Supplementary material for: A highly stretchable tri-channel fiber for composite motion decoupling
Source: Nat Commun. 2026 Jun 12;17:7465. doi: 10.1038/s41467-026-73959-4 (PMC13408763; doi:10.1038/s41467-026-73959-4)
Supplement: Supplementary file 1 — Supplementary Information [file 41467_2026_73959_MOESM1_ESM.pdf]

Supplementary Information for

A highly stretchable tri-channel fiber for composite motion decoupling

Zhangcheng Li[1], Wen Wang[1], Kangrui Ji[1], Can Wang[1], Pan Xiong[1], Zeguang Du[1], Zhi Liang[1], Yu He[1], Bijin Xiong[2] & Chong Hou[1,3,4]*

[1] State Key Laboratory of New Textile Materials and Advanced Processing, Research Center for Intelligent Fiber Devices and Equipment and School of Optical and Electronic Information, Huazhong University of Science and Technology, Wuhan 430074, China.

[2] Key Laboratory of Materials Chemistry for Energy Conversion and Storage of Ministry of Education, School of Chemistry and Chemical Engineering, Huazhong University of Science and Technology, Wuhan 430074, China.

[3] School of Intelligent Sports Engineering, Engineering Research Center of Sports Health Intelligent Equipment of Hubei Province, Key Laboratory of Sports Engineering of General Administration of Sports of China, Wuhan Sports University, Wuhan 430079, China.

[4] Shenzhen Huazhong University of Science and Technology Research Institute, Shenzhen 518057, China.

Table of contents

This file includes:

Supplementary Note 1-6

Supplementary Fig. 1-26

Supplementary Table 1-2

Supplementary Note 1 | Effect of drawing and rotation speed on the helical microchannels structure within the tri-channel fiber during thermal drawing.

Supplementary Note 2 | Analysis of microphase separation structure evolution and polymer chains orientation behavior in tri-channel fibers under tensile strain.

Supplementary Note 3 | Analysis of the mechanical enhancement and deformation characteristics of the tri-channel fiber with helically oriented polymer chains.

Supplementary Note 4 | Calculation of the resistive and capacitive response of the dual-strain fiber sensor under pure tensile strain.

Supplementary Note 5 | Calculation of the capacitive response of double helical microchannel within the dual-strain fiber sensor under pure torsional strain.

Supplementary Note 6 | Real-time monitoring and computational decoupling of simultaneous tensile and torsional strains using the dual-strain fiber sensor.

Supplementary Fig. 1 | Fabrication of the tri-channel fiber preform and photograph of the thermal drawing fiber.

Supplementary Fig. 2 | Thermal drawing tower and drawing parameters.

Supplementary Fig. 3 | Thermodynamic and rheological properties of SEBS.

Supplementary Fig. 4 | Effect of drawing and rotation speed on the helical microchannels structure within the fiber during thermal drawing.

Supplementary Fig. 5 | Schematic diagrams and SAXS results of samples with different morphologies.

Supplementary Fig. 6 | SAXS properties of the preform and tri-channel fibers.

Supplementary Fig. 7 | Schematic illustration of structural evolution in PS domains

during thermal drawing processing and mechanical loading.

Supplementary Fig. 8 | WAXS properties of the tri-channel fiber under various tensile strain.

Supplementary Fig. 9 | Comparative mechanical properties of microchanneled fibers and their solid counterparts fabricated via rotational versus non-rotational drawing. The helical period (h) of the rotationally drawn fibers is fixed at 5mm.

Supplementary Fig. 10 | Mechanical properties and mechanical cycle performance of the tri-channel fiber.

Supplementary Fig. 11 | Cyclic tensile durability of fibers with straight versus helical microchannels.

Supplementary Fig. 12 | Comparison of the maximum cycles of straight and helical microchannels under different tensile strains.

Supplementary Fig. 13 | Digital microscopic images of the dual-strain fiber sensor under stretching and bidirectional twisting.

Supplementary Fig. 14 | 3D XRM reconstructions and cross-sectional CT slices of the tri-channel fiber under different torsional strains.

Supplementary Fig. 15 | Changes in the internal helical microchannels of the dual-strain fiber sensor under tensile and torsional strain.

Supplementary Fig. 16 | Working mechanism of the dual-strain fiber sensor for tensile and torsional strain sensing.

Supplementary Fig. 17 | Resistive electromechanical response characteristics of dual-strain fiber sensors.

Supplementary Fig. 18 | Relative capacitance changes of the dual-strain fiber sensor with different h under tensile strain and theoretical calculation results.

Supplementary Fig. 19 | Relative capacitance changes of the dual-strain fiber sensor with different h under torsional strain and theoretical calculation results.

Supplementary Fig. 20 | Electrical hysteresis characteristics of fiber sensors.

Supplementary Fig. 21 | Resistive and capacitive responses of the sensor to tensile strain under various fixed torsional strains.

Supplementary Fig. 22 | Resistive and capacitive response curves of the dual-strain

fiber sensor under tensile and torsional strains at various loading rates.

Supplementary Fig. 23 | Comparison of the performance of dual-strain fiber sensor with previously reported strain sensors.

Supplementary Fig. 24 | Decoupling performance under composite strain combinations. Torsional strains are presented as absolute values. Values above the error bars represent the mean, and error bars indicate the standard deviation (n = 10 independent samples).

Supplementary Fig. 25 | Real-time decoupling performance under constant load and varying pre-twist turns.

Supplementary Fig. 26 | Real-time decoupling performance under constant pre-twist turns and varying loads.

Supplementary Table 1 | The comparison of the sensing performance of the dual-strain fiber sensor with the reported literature of flexible strain sensors.

Supplementary Table 2 | SAXS/WAXS sample details, data collection, data analysis, and software used.

Other Supplementary Materials for this manuscript include the following:

Movie S1 | Dual-strain fiber sensor for visual monitoring and decoupling composite motion states.

Supplementary Note 1 | Effect of drawing and rotation speed on the helical microchannels structure within the tri-channel fiber during thermal drawing.

The fabrication of tri-channel fiber through thermal drawing is critically dependent on several key processing parameters, including the processing temperature, drawing speed, and preform rotation speed. These parameters are pivotal in ensuring the formation of microchannels with a well-defined morphology and robust structural integrity in the resulting fibers. Supplementary Fig. 3a illustrates the storage modulus (G'), loss modulus (G"), and complex viscosity of SEBS as a function of temperature. During the fabrication of tri-channel fiber, the material must exhibit viscous flow, wherein the loss modulus predominates at the processing temperature, as well as enable flow at high viscosities (exceeding 10^3 Pa·s)[1]. Upon stretching, the internal polymer chains of the material mobilize and align along the direction of elongation, ultimately leading to fiber formation. High viscosity helps mitigate capillary rupture of thin layers in preform, promote the amalgamation of flowing materials during thermal drawing, and contribute to the preservation of intricate fiber microstructures and shapes[2]. Consequently, the processing temperature range was set at 140–180 °C. The dimensions of fibers fabricated via thermal drawing meet the following conditions[3]:

$$d_{fiber} = D_{preform}\sqrt{\frac{v_f}{v_d}} \tag{1}$$

where d_{fiber} is the diameter of the fiber, $D_{preform}$ is the diameter of the preform, v_f is the preform feeding speed, and v_d is the drawing speed. By adjusting the ratio of v_f to v_d, the fiber size can be effectively controlled.

When the preform with hollow channels, as shown in Supplementary Fig 1, is rotated during the thermal drawing process, the polymer chains in the material will extend in a helical orientation along the fiber axis due to the combined effects of stretching and rotation. Additionally, helical microchannels will be formed in the fiber, as illustrated in Fig. 1a. The helical period h of the microchannels can be controlled by the drawing speed v_d and the rotation speed v_r:

$$h = \frac{v_d}{v_r} \tag{2}$$

Supplementary Fig 2 shows the thermal drawing tower employed for the fabrication of tri-channel fiber, highlighting the regulation of various parameters throughout the thermal drawing process and the control over fiber diameter.

As shown in Supplementary Fig. 3b, SEBS exhibits shear thinning behavior in its viscous flow regime. This implies that excessive values of v_d and v_r during the thermal drawing can lead to a reduction in the material's viscosity. Furthermore, due to the Weissenberg effect[4], the material at the edges of the preform flows toward the center during its rotation, which hinders both fiber formation and the stability of the fiber and its internal structure. As shown in Supplementary Fig. 4, as v_r increases, the h of the microchannel in the fiber decreases, which also affects the microchannel structure. Specifically, when h is less than 1.7 mm, the helical microchannels structure severely damaged, which impedes the subsequent construction of the liquid metal conductive pathway and compromises the stability of the device. Therefore, during the thermal drawing process, v_r and v_d should be minimized to preserve the integrity of the fiber and its internal structure.

Supplementary Note 2 | Analysis of microphase separation structure evolution and polymer chains orientation behavior in tri-channel fibers under tensile strain.

To investigate the effect of rotating the preform during thermal drawing on the microphase separation structure, polymer chains orientation and mechanical properties of the resulting tri-channel fibers, a series of SAXS/WAXS and mechanical property tests were conducted on the preform, and different types of tri-channel fibers.

Specifically, in fibers with helically aligned polymer chains along the axial direction, the front and rear sides exhibit a symmetric orientation distribution during SAXS testing (as shown in Supplementary Fig. 5a). As a result, the scattering patterns originating from the front and rear sides are symmetric about the fiber axis. The superposition of these symmetric signals yields an elliptical scattering pattern (Supplementary Fig. 5b), with its long axis perpendicular to the fiber axis. To unambiguously resolve the polymer chain orientation, the fibers were longitudinally sectioned into semi-cylindrical specimens prior to SAXS characterization

(Supplementary Fig. 5c). This approach enables the direct and accurate determination of the orientation angle of the polymer chains relative to the fiber axis, as evidenced in Supplementary Fig. 5d.

Supplementary Fig. 6 shows 2D SAXS patterns of the preform, the fibers containing straight/helical microchannels in their initial relaxed states. The 2D SAXS pattern of the preform exhibits an isotropic circular scattered ring, indicating spherical PS domains and randomly oriented polymer chains. Supplementary Fig. 6d shows 1D SAXS profiles derived from 2D patterns of the preform and the fiber with h = 5 mm under incremental tensile strains. The preform SAXS profile feature a primary reflection peak at $1q$ = 0.254 nm^{-1}, corresponding to a PS domain d-spacing of $\approx$24.73 nm.

After thermal drawing, fiber SAXS patterns transition from circular to elliptical scattering rings (Supplementary Fig. 6b and 6c). The scattering vector component parallel to the drawing direction ($q_{\parallel}$) decreases, whereas the perpendicular component ($q_{\perp}$) increases. This evolution signifies elongation of PS domains along the drawing direction, forming ellipsoidal microdomains with major axes aligned parallel to this direction (Supplementary Fig. 7a). Concurrently, polymer chains reorient from random distribution to alignment perpendicular to the elliptical scattering ring's major axis. Fig. 2b demonstrates that increasing microchannel helicity progressively augments the inclination angle of the scattering ellipse's major axis. This confirms that angular forces induced by preform rotation during thermal drawing govern PS domain and polymer chain orientation within fibers. Furthermore, 1D SAXS profiles of relaxed fibers (Supplementary Fig. 6d) reveal broadening of the primary peak's full width at half maximum relative to the preform, and other reflection peaks almost vanish. This indicates thermal drawing disrupts weakly ordered PS domain structures in fibers.

Fig. 2c reveals progressive flattening of the scattering elliptical rings in 2D SAXS patterns for both straight and helical tri-channel fibers with increasing tensile strain. Correspondingly, q component parallel to the major axis of elliptical scattering ring decreases while the perpendicular component increases. This signifies increased d-spacing between PS domains along the tensile axis but decreased spacing radially,

implying polymer chain elongation parallel to the stretching direction. Concurrently, PS domains morphology evolves under strain as schematized in Supplementary Fig. 7b and 7c. For fiber with straight microchannels, the PS domains orientation angle θ—defined as the diagonal angle of its circumscribed rectangle—aligns with the fiber axis. Where a and b denote the semi-major and semi-minor axes, respectively. Under tensile strain, the PS domain's semi-major axis (a') increases while both the semi-minor axis (b') and orientation angle (θ') decrease progressively. Conversely, in fiber with helical microchannels, the PS domains orientation angle θ exhibits an inclination angle δ relative to the fiber axis. During stretching, δ decreases toward the fiber axis while θ deflects toward alignment with fiber axis. This dual reorientation indicates deflection and realignment of both PS domains and polymer chains along the tensile direction. Supplementary Fig. 6d demonstrates complete disappearance of the reflection peak in 1D SAXS profiles at >100% tensile strain, with curve stability upon further straining. This indicates full orientation of PS domains and polymer chains along the tensile axis.

Supplementary Fig. 8 shows 2D WAXS patterns and corresponding 1D scattering profiles of the fiber under incremental tensile strains, obtained from the azimuthal angle integration of the 2D patterns. As the tensile strain increases, the bright arcs perpendicular to the tensile direction progressively concentrate, indicating that the orientation of the molecular segments within the fiber is enhanced with the increasing tensile strain. No crystallization signal appears during the stretching process, and the anisotropic amorphous ring persists throughout the deformation process. The deformed WAXS diffraction pattern is interpreted as a superposition of the oriented molecular chain diffraction pattern and the residual amorphous halo[5].

Supplementary Note 3 | Analysis of the mechanical enhancement and deformation characteristics of the tri-channel fiber with helically oriented polymer chains.

Mechanical characterization demonstrates that the polymer chains orientation within fibers significantly influences their mechanical properties. The preform rotation during thermal drawing induces helical polymer chains orientation along the fiber axis,

establishing a spring-like three-dimensional network architecture. This enables efficient stress distribution and energy dissipation along the fiber axis, enhancing tensile strength and elongation at break relative to the raw material. To decouple the geometric influence of internal macroscopic channels on mechanical performance, solid SEBS fiber equivalents were fabricated using both rotational and non-rotational drawing protocols. These solid fibers were systematically benchmarked against their microchanneled counterparts to elucidate the contribution of helically oriented polymer chains to mechanical properties (Supplementary Fig. 9). Notably, the rotationally drawn solid fibers exhibited markedly enhanced mechanical performance relative to the non-rotational controls, with elongation at break increasing from 804.2% to 2063.2% and breaking strength rising from 9.21 MPa to 16.73 MPa.

Additionally, the non-rotationally drawn solid SEBS fiber shows similar elongation at break to the non-rotationally drawn SEBS fiber with straight microchannels, but the solid structure exhibits slightly higher breaking strength due to the absence of stress concentration caused by internal microchannels. Similarly, the rotationally drawn solid fiber demonstrates higher elongation at break and breaking strength than its counterpart with helical microchannels. This enhanced performance stems from the inherent structural integrity of the solid fiber, which circumvents potential local collapse or interfacial defects characteristic of microchannels during stretching. Consequently, the strengthening and toughening effects imparted by the helically oriented polymer chains are maximized. Furthermore, in the initial strain regime, non-rotationally drawn fibers exhibit a higher yield stress, attributable to the alignment of polymer chains parallel to the loading axis. In contrast, the helically oriented chains in rotationally drawn fibers lower the energy barrier for deformation onset, resulting in a reduced initial modulus.

Collectively, by eliminating the influence of macroscopic channel geometry, these findings substantiate that the helical chain orientation induced by rotational thermal drawing serves as the primary driver of the observed mechanical enhancement. Further investigation focused on the mechanical properties of fibers varying in helical polymer chain orientation and channel structure, as shown in Supplementary Fig. 10a.

Importantly, the ultimate elongation of the fibers does not follow a simple

negative correlation with the h. Specifically, the measured fracture strains are 805% (h=∞), 891% (h=50 mm), 1,076% (h=25 mm), 1,578% (h=10 mm), 1,918% (h=5 mm), 1,222% (h=2.5 mm). This phenomenon originates from the interplay between microscopic chain orientation and macroscopic structural features. At a moderate h, the tilt angle δ between the polymer chains and the fiber axis reaches an optimal value. This configuration facilitates effective energy dissipation through chain uncoiling during stretching, while maintaining relatively mild stress concentration within the helical channels, thereby collectively contributing to significantly enhanced toughness. However, when h is excessively small, the resulting large tilt angle δ reduces the axial load-bearing capacity. Concurrently, the high density of helical channels induces pronounced stress concentration, promoting localized deformation and accelerating defect propagation. These factors collectively lead to a reduction in the elongation at break. This behavior fundamentally reflects a trade-off between the toughening effect conferred by the helical orientation and the degradation in load-bearing capacity induced by an excessively small helical period.

To further elucidate the influence of the microchannel filler on mechanical performance, we characterized the tensile stress-strain response of the tri-channel fiber infused with liquid metal (Supplementary Fig. 10b). The results demonstrate a slight reduction in the fiber's elongation at break but a corresponding increase in its fracture strength upon liquid metal infusion. This change stems from two competing mechanisms: on one hand, the infiltration of liquid metal may accelerate the propagation of microcracks initiating within the fiber at high strains, leading to a premature fracture[6]; on the other hand, the liquid metal filler may enhance the fiber's load-bearing capacity through interfacial interaction and local stress redistribution, which manifests as the observed increase in fracture strength[7]. Collectively, this indicates that employing liquid metal as a filler material does not substantially alter the mechanical behavior of the fiber, underscoring that structural design and microscopic polymer chain orientation remain the dominant factors governing its tensile properties.

The mechanical hysteresis behaviors of the fiber sensor are presented in Supplementary Fig. 10c and 10d. As shown in Supplementary Fig. 10c, the elastic

performance of the fiber was evaluated under progressively increasing tensile strains ranging from 100% to 500%. Throughout this strain range, the resultant stress-strain curves exhibit characteristic hysteresis, and the area of the hysteresis loop increases proportionally with the applied tensile strain. Furthermore, under cyclic loading at a fixed 100% tensile strain (Supplementary Fig. 10d), the mechanical hysteresis loop area was maximized during the initial cycle. This phenomenon is primarily attributed to energy dissipation arising from chain slippage and internal friction during the straining process, combined with the reorganization and dissipation within the microscopic two-phase structure following the initial large deformation[8]. In subsequent cycles, the loop area gradually diminished and subsequently stabilized. This trend suggests that the elastic network progressively attained a dynamic equilibrium following repeated stretching-recovery cycles, resulting in a more reversible mechanical response.

Electrical signal stability testing during tensile cycling reveals that fibers with helical microchannels exhibit superior cycle life across strain ranges versus straight microchannels counterparts (Supplementary Fig. 11 and Supplementary Fig. 12).

Supplementary Fig. 13 illustrates the morphological evolution of the dual-strain fiber sensor under tensile and torsional strains. Under tensile strain, a reduction in fiber diameter is accompanied by an increase in the h of the internal helical microchannels. In contrast, under positive and reverse torsional strains, while the fiber diameter remains nearly constant, the h decreases and increases correspondingly. Supplementary Fig. 15a and 15b quantify the dependence of the helical angle on applied tensile and torsional strains. Excessive torsional strain induces fiber snarling (as shown in the inset of Supplementary Fig. 15c) due to axial stress accumulation, and higher tensile strains elevate the threshold torsional strain required to initiate snarling.

Supplementary Note 4 | Calculation of the resistive and capacitive response of the dual-strain fiber sensor under pure tensile strain.

To calculate the variation in internal resistance and capacitance of dual-strain fiber sensor under pure tensile strain, it is essential to analyze the structural changes of multiple liquid metal microchannels subjected to the applied strain. Within the fiber,

the concentric microchannel serves as the resistance output channel, while the double helical microchannel functions as the capacitance output channel, as shown in Fig. 3a and Supplementary Fig. 16a. The two electrical signals generate distinct response behaviors under tensile strain, which will be discussed separately in the following sections.

Initially, the resistance channel is aligned coaxially with the fiber. When the fiber is in its relaxed state, the initial resistance R_0 output by the resistance channel is[9]:

$$R_0 = \rho \frac{L_0}{S_0} = \rho \frac{L_0}{\pi {r_0}^2} \tag{3}$$

where ρ is the resistivity of the liquid metal, L_0 is the initial length of the resistance channel, which is also the initial length of the fiber, S_0 is the initial cross-sectional area of the resistance channel, and r_0 is the initial radius of the resistance channel, as shown in Supplementary Fig. 16a and b. Under tensile strain ε, the volume of the liquid metal in the resistance channel, $V = L_0 \times S_0 = L_0 \times \pi {r_0}^2$, remains constant. Consequently, the length $L(\varepsilon)$ of the fiber and the resistance channel, as well as the radius $r(\varepsilon)$ of the resistance channel, change as follows:

$$L(\varepsilon) = (1+\varepsilon)L_0 \tag{4}$$

$$r(\varepsilon) = \frac{r_0}{\sqrt{(1+\varepsilon)}} \tag{5}$$

Therefore, when subjected only to tensile strain ε, the resistance $R(\varepsilon)$ and its relative changes $\Delta R/R_0(\varepsilon)$ as measured by the resistance channel, are given by the following expressions:

$$R(\varepsilon) = \rho \frac{L}{\pi r^2} = \rho \frac{(1+\varepsilon)^2 L_0}{\pi {r_0}^2} \tag{6}$$

$$\frac{\Delta R}{R_0}(\varepsilon) = (\varepsilon+1)^2 - 1 \tag{7}$$

These results demonstrate quadratic dependence of resistance on tensile strain in the sensing channel (Fig. 3b and 3c). Supplementary Fig. 17 details additional electromechanical characteristics, including resistance stability during cyclic loading.

On the other hand, the double helical microchannel in the fiber functions as the capacitance output channel, which can be equivalent to the capacitance between two parallel wires. When the fiber is in its initial relaxed state, the radius of the helical

microchannel is r_0, the distance between the centers of the two helical channels is d_0, the helical period is h_0, and the angle between the helical microchannel and the fiber axis is α. We unscrew the helical channel, as shown in Supplementary Fig. 16a, and the length of one turn of the unscrewed helical l_{1-turn_0} is:

$$l_{1-turn_0} = \sqrt{{h_0}^2 + (\pi d_0)^2} \tag{8}$$

In a fiber with an initial length of L_0, the length l_0 of the helical microchannel after unscrewed is:

$$l_0 = L_0\sqrt{1 + (\pi d_0/h_0)^2} \tag{9}$$

Therefore, the initial capacitance C_0 output by the capacitance channel when the fiber is in the relaxed state can be calculated as[10]:

$$C_0 = \frac{\pi \epsilon l_0}{\ln\left(\frac{d_0}{2r_0} + \sqrt{\left(\frac{d_0}{2r_0}\right)^2 - 1}\right)} = \frac{\pi \epsilon L_0\sqrt{1 + (\pi d_0/h_0)^2}}{\ln\left(\frac{d_0}{2r_0} + \sqrt{\left(\frac{d_0}{2r_0}\right)^2 - 1}\right)} \tag{10}$$

where ϵ is the dielectric constant between the two helical microchannels. When the tensile strain is ε, the helical period $h(\varepsilon)$ of the helical microchannel and the center distance $d(\varepsilon)$ of the two helical microchannels will change as follows:

$$h(\varepsilon) = (1 + \varepsilon)h_0 \tag{11}$$

$$d(\varepsilon) = (1 + \varepsilon)^{-\nu} d_0 \tag{12}$$

where ν is the Poisson's ratio of SEBS. Since SEBS is a highly elastic material, its Poisson's ratio can be approximately equal to 0.5,[11] and the volume of the liquid metal in the microchannel $V = \pi {r_0}^2 l_0 = \pi r(\varepsilon)^2 l(\varepsilon)$, remains constant. Consequently, it can be calculated that when the tensile strain is ε, the length of a single turn of the unscrewed helix $l_{1-turn}(\varepsilon)$, the length $l(\varepsilon)$, and the radius $r(\varepsilon)$ of the helical microchannel are:

$$l_{1-turn}(\varepsilon) = \sqrt{h(\varepsilon)^2 + \left(\pi d(\varepsilon)\right)^2} = \sqrt{(1 + \varepsilon)^2 {h_0}^2 + \frac{(\pi d_0)^2}{1 + \varepsilon}} \tag{13}$$

$$l(\varepsilon) = \frac{L(\varepsilon)}{h(\varepsilon)} \times l_{1-turn}(\varepsilon) = L_0\sqrt{(1 + \varepsilon)^2 + \frac{(\pi d_0/h_0)^2}{1 + \varepsilon}} \tag{14}$$

$$r(\varepsilon) = r_0 \left(\frac{(1+\varepsilon)(1+(\pi d_0/h_0)^2)}{(1+\varepsilon)^3 + (\pi d_0/h_0)^2} \right)^{\frac{1}{4}} \tag{15}$$

Therefore, the capacitance $C(\varepsilon)$ and its relative change $\frac{\Delta C}{C_0}(\varepsilon)$, measured via the capacitance channel under an applied tensile strain ε, are described by the following relationships:

$$C(\varepsilon) = \frac{\pi \epsilon L_0 (1+\varepsilon) \sqrt{1 + \frac{\left(\frac{\pi d_0}{h_0}\right)^2}{(1+\varepsilon)^3}}}{\ln\left\{ \frac{d_0}{2r_0} \left(\frac{(1+\varepsilon)^3 + \left(\frac{\pi d_0}{h_0}\right)^2}{(1+\varepsilon)^3 \left(1 + \left(\frac{\pi d_0}{h_0}\right)^2\right)} \right)^{\frac{1}{4}} + \sqrt{\left(\frac{d_0}{2r_0}\right)^2 \sqrt{\frac{(1+\varepsilon)^3 + \left(\frac{\pi d_0}{h_0}\right)^2}{(1+\varepsilon)^3 \left(1 + \left(\frac{\pi d_0}{h_0}\right)^2\right)}} - 1} \right\}} \tag{16}$$

$$\frac{\Delta C}{C_0}(\varepsilon) = (1+\varepsilon) \sqrt{\frac{(1+\varepsilon)^3 + \left(\frac{\pi d_0}{h_0}\right)^2}{(1+\varepsilon)^3 \left(1 + \left(\frac{\pi d_0}{h_0}\right)^2\right)}} \times \frac{\ln\left(\frac{d_0}{2r_0} + \sqrt{\left(\frac{d_0}{2r_0}\right)^2 - 1} \right)}{\ln\left\{ \frac{d_0}{2r_0} \left(\frac{(1+\varepsilon)^3 + \left(\frac{\pi d_0}{h_0}\right)^2}{(1+\varepsilon)^3 \left(1 + \left(\frac{\pi d_0}{h_0}\right)^2\right)} \right)^{\frac{1}{4}} + \sqrt{\left(\frac{d_0}{2r_0}\right)^2 \left(\frac{(1+\varepsilon)^3 + \left(\frac{\pi d_0}{h_0}\right)^2}{(1+\varepsilon)^3 \left(1 + \left(\frac{\pi d_0}{h_0}\right)^2\right)} \right)^{\frac{1}{2}} - 1} \right\}} - 1$$

$$= (1+\varepsilon)\sqrt{Q} \times \frac{ln\left(A + \sqrt{A^2 - 1}\right)}{ln\left(AQ^{\frac{1}{4}} + \sqrt{A^2 Q^{\frac{1}{2}} - 1}\right)} - 1 \tag{17}$$

where $A = \frac{d_0}{2r_0}$ is a parameter related to the microchannel diameter, $B = \frac{\pi d_0}{h_0}$ is a parameter related to the helical period, and $Q = \frac{(1+\varepsilon)^3 + B^2}{(1+\varepsilon)^3 (1+B^2)}$.

As shown in Supplementary Fig. 17, the relative change in capacitance of double helical microchannel with different h during tensile strain exhibits a linear response to the applied strain. When $h_0 = \infty$, the gauge factor (GF) is 1, and h has minimal effect on the capacitive response and results. This capacitive linear response characteristic can effectively improve the stability of the sensor and reduce errors.

Supplementary Note 5 | Calculation of the capacitive response of double helical microchannel within the dual-strain fiber sensor under pure torsional strain.

The resistance exhibits negligible variation under pure torsional deformation, as the concentric microchannel for outputting the signal remains largely unaffected by

such mechanical strain, as evidenced in Supplementary Fig 14. Therefore, the pure torsional strain of the fiber can be monitored through the capacitance signal output by the double helical microchannel. As shown in Supplementary Fig. 16d, during the fiber torsion process, the length and cross-sectional area of the fiber remain constant, while the center distance between the two helical microchannels and the volume of the liquid metal in the channel $V = \pi {r_0}^2 l_0 = \pi r(\gamma)^2 l(\gamma)$, also remain constant. Consequently, it can be calculated that when the torsion level is γ, the helical period $h(\gamma)$ of the helical microchannel, the length $l_{1-turn}(\gamma)$ of the unscrewed helical one turn, the length $l(\gamma)$ of the unscrewed helical microchannel in the fiber, and the radius $r(\gamma)$ of the microchannel are respectively:

$$h(\gamma) = \frac{L_0}{\left(\frac{L_0}{h_0} + \frac{\gamma L_0}{2\pi}\right)} = \frac{2\pi h_0}{2\pi + h_0 \gamma} \tag{18}$$

$$l_{1-turn}(\gamma) = \sqrt{h(\gamma)^2 + (\pi d_0)^2} = \sqrt{\left(\frac{2\pi h_0}{2\pi + h_0 \gamma}\right)^2 + (\pi d_0)^2} \tag{19}$$

$$l(\gamma) = \frac{L_0}{h(\gamma)} \times l_{1-turn}(\gamma) = L_0 \sqrt{1 + \left(\frac{\pi d_0}{h_0} + \frac{\gamma d_0}{2}\right)^2} \tag{20}$$

$$r(\gamma) = r_0 \sqrt{\frac{l_0}{l(\gamma)}} = r_0 \left(\frac{1 + \left(\frac{\pi d_0}{h_0}\right)^2}{1 + \left(\frac{\pi d_0}{h_0} + \frac{\gamma d_0}{2}\right)^2}\right)^{\frac{1}{4}} \tag{21}$$

Hence, the capacitance $C(\gamma)$ and its relative change $\frac{\Delta C}{C_0}(\gamma)$, measured via the capacitance channel under an applied torsion level γ, are described by the following relationships:

$$C(\gamma) = \frac{\pi \epsilon L_0 \sqrt{1 + \left(\frac{\pi d_0}{h_0} + \frac{\gamma d_0}{2}\right)^2}}{ln\left(\frac{d_0}{2r_0}\left(\frac{1 + \left(\frac{\pi d_0}{h_0} + \frac{\gamma d_0}{2}\right)^2}{1 + \left(\frac{\pi d_0}{h_0}\right)^2}\right)^{\frac{1}{4}} + \sqrt{\left(\frac{d_0}{2r_0}\right)^2 \left(\frac{1 + \left(\frac{\pi d_0}{h_0} + \frac{\gamma d_0}{2}\right)^2}{1 + \left(\frac{\pi d_0}{h_0}\right)^2}\right)^{\frac{1}{2}} - 1}\right)} \tag{22}$$

$$\frac{\Delta C}{C_0}(\gamma) = \sqrt{\frac{1+\left(\frac{\pi d_0}{h_0}+\frac{\gamma d_0}{2}\right)^2}{1+\left(\frac{\pi d_0}{h_0}\right)^2}} \times \frac{\ln\left(\frac{d_0}{2r_0}+\sqrt{\left(\frac{d_0}{2r_0}\right)^2-1}\right)}{\ln\left(\frac{d_0}{2r_0}\left(\frac{1+\left(\frac{\pi d_0}{h_0}+\frac{\gamma d_0}{2}\right)^2}{1+\left(\frac{\pi d_0}{h_0}\right)^2}\right)^{\frac{1}{4}}+\sqrt{\left(\frac{d_0}{2r_0}\right)^2\left(\frac{1+\left(\frac{\pi d_0}{h_0}+\frac{\gamma d_0}{2}\right)^2}{1+\left(\frac{\pi d_0}{h_0}\right)^2}\right)^{\frac{1}{2}}-1}\right)} - 1$$

$$= \sqrt{P} \times \frac{\ln\left(A+\sqrt{A^2-1}\right)}{\ln\left(AP^{\frac{1}{4}}+\sqrt{A^2P^{\frac{1}{2}}-1}\right)} - 1 \tag{23}$$

Where $T = \frac{\gamma d_0}{2}$ is a parameter related to the torsion level, and $P = \frac{1+\left(\frac{\pi d_0}{h_0}+\frac{\gamma d_0}{2}\right)^2}{1+\left(\frac{\pi d_0}{h_0}\right)^2} = \frac{1+(B+T)^2}{1+B^2}$.

As shown in Fig. 4b and Supplementary Fig. 19, the dual-strain fiber sensors with different h exhibit significant differences in their response properties under torsional strain. As h decreases, the lowest point of capacitance reduction caused by reverse twisting becomes smaller, and the corresponding reverse torsion level increases. Additionally, the sensitivity to capacitive response during positive twisting also increases. Therefore, to achieve a larger torsional strain sensing range and increased torsional strain sensitivity, h should be minimized while ensuring that the dual-strain fiber sensor maintains good linearity and response under tensile strain.

Supplementary Note 6 | Real-time monitoring and computational decoupling of simultaneous tensile and torsional strains using the dual-strain fiber sensor.

When the fiber strain sensor is subjected to both tensile and torsional strains simultaneously, the resistive signal responds exclusively to tensile strain, remaining insensitive to torsion; in contrast, the capacitive response arises from the superposition of both stimuli. Consequently, the resistance $R(\varepsilon,\gamma)$, capacitance $C(\varepsilon,\gamma)$ and their relative change $\frac{\Delta R}{R_0}(\varepsilon,\gamma)$ and $\frac{\Delta C}{C_0}(\varepsilon,\gamma)$ under composite tensile strain ε and torsion level γ are described by the following relationships:

$$R(\varepsilon,\gamma) = \rho\frac{L}{\pi r^2} = \rho\frac{(1+\varepsilon)^2 L_0}{\pi {r_0}^2} \tag{24}$$

$$\frac{\Delta R}{R_0}(\varepsilon,\gamma) = (\varepsilon+1)^2 - 1 \tag{25}$$

$$C(\varepsilon,\gamma) = \frac{\pi\epsilon L_0 \sqrt{\frac{(1+\varepsilon)^3 + \left[\frac{\pi d_0}{h_0} + (1+\varepsilon)\frac{\gamma d_0}{2}\right]^2}{(1+\varepsilon)}}}{\ln\left\{\frac{d_0}{2r_0}\left\{\frac{(1+\varepsilon)^3 + \left[\frac{\pi d_0}{h_0} + (1+\varepsilon)\frac{\gamma d_0}{2}\right]^2}{(1+\varepsilon)^3\left[1+\left(\frac{\pi d_0}{h_0}\right)^2\right]}\right\}^{\frac{1}{4}} + \sqrt{\left(\frac{d_0}{2r_0}\right)^2\left\{\frac{(1+\varepsilon)^3 + \left[\frac{\pi d_0}{h_0} + (1+\varepsilon)\frac{\gamma d_0}{2}\right]^2}{(1+\varepsilon)^3\left[1+\left(\frac{\pi d_0}{h_0}\right)^2\right]}\right\}^{\frac{1}{2}} - 1}\right\}} \tag{26}$$

$$\frac{\Delta C}{C_0}(\varepsilon,\gamma) = \sqrt{\frac{(1+\varepsilon)^3 + \left[\frac{\pi d_0}{h_0} + (1+\varepsilon)\frac{\gamma d_0}{2}\right]^2}{(1+\varepsilon)\left[1+\left(\frac{\pi d_0}{h_0}\right)^2\right]}}\frac{\ln\left(\frac{d_0}{2r_0} + \sqrt{\left(\frac{d_0}{2r_0}\right)^2 - 1}\right)}{\ln\left\{\frac{d_0}{2r_0}\left\{\frac{(1+\varepsilon)^3 + \left[\frac{\pi d_0}{h_0} + (1+\varepsilon)\frac{\gamma d_0}{2}\right]^2}{(1+\varepsilon)^3\left[1+\left(\frac{\pi d_0}{h_0}\right)^2\right]}\right\}^{\frac{1}{4}} + \sqrt{\left(\frac{d_0}{2r_0}\right)^2\left\{\frac{(1+\varepsilon)^3 + \left[\frac{\pi d_0}{h_0} + (1+\varepsilon)\frac{\gamma d_0}{2}\right]^2}{(1+\varepsilon)^3\left[1+\left(\frac{\pi d_0}{h_0}\right)^2\right]}\right\}^{\frac{1}{2}} - 1}\right\}} - 1$$

$$= \lambda\sqrt{Z}\frac{\ln\left(A + \sqrt{A^2 - 1}\right)}{\ln\left\{AZ^{\frac{1}{4}} + \sqrt{A^2 Z^{\frac{1}{2}} - 1}\right\}} - 1 \tag{27}$$

Where $\lambda = (1+\varepsilon)$ is a parameter related to the tensile strain, $\beta = \frac{\gamma h_0}{2\pi}$ is a parameter related to the torsion level, and $Z = \frac{(1+\varepsilon)^3 + \left[\frac{\pi d_0}{h_0} + (1+\varepsilon)\frac{\gamma d_0}{2}\right]^2}{(1+\varepsilon)^3\left(1+\left(\frac{\pi d_0}{h_0}\right)^2\right)} = \frac{\lambda^3 + \left[\frac{\pi d_0}{h_0}(1+\lambda\beta)\right]^2}{\lambda^3\left[1+\left(\frac{\pi d_0}{h_0}\right)^2\right]}$.

The dual-strain decoupling algorithm proceeds in three sequential steps:

1. Calibration and modeling: The resistance and capacitance responses of the fiber sensor were measured under predetermined combinations of tensile and torsional strains (Fig. 4f). These datasets were used to calibrate the dual-strain sensor response, leading to a numerical model (Fig. 5a) established via nonlinear polynomial fitting and modeling analysis. The model was subsequently validated against preset composite strain conditions to ensure decoupling precision during real-time dynamic monitoring (Fig. 5b).

2. Tensile strain quantification: The tensile strain ε is first quantified directly from the relative resistance change $\frac{\Delta R}{R_0}$ using Equation (25), independent of torsion (Fig. 5a, left).

3. Torsional strain decoupling: The computed tensile strain ε is substituted into the Equation (27) alongside the measured relative capacitance variation $\frac{\Delta C}{C_0}$ to isolate and quantify the torsional strain (Fig. 5a, right).

Error analysis of composite strain sensing and decoupling: The sensor demonstrates a relative tensile strain error of <1%, attributed to its highly consistent resistive response to tension and negligible cross-sensitivity to torsion. For torsional decoupling based on capacitive signals, the relative error is maintained within 8%, where deviations principally arise from signal fluctuations induced by superimposed tensile loading. To guarantee sustained fidelity during long-term dynamic monitoring, a periodic recalibration protocol at the zero-strain baseline is implemented to mitigate potential drift.

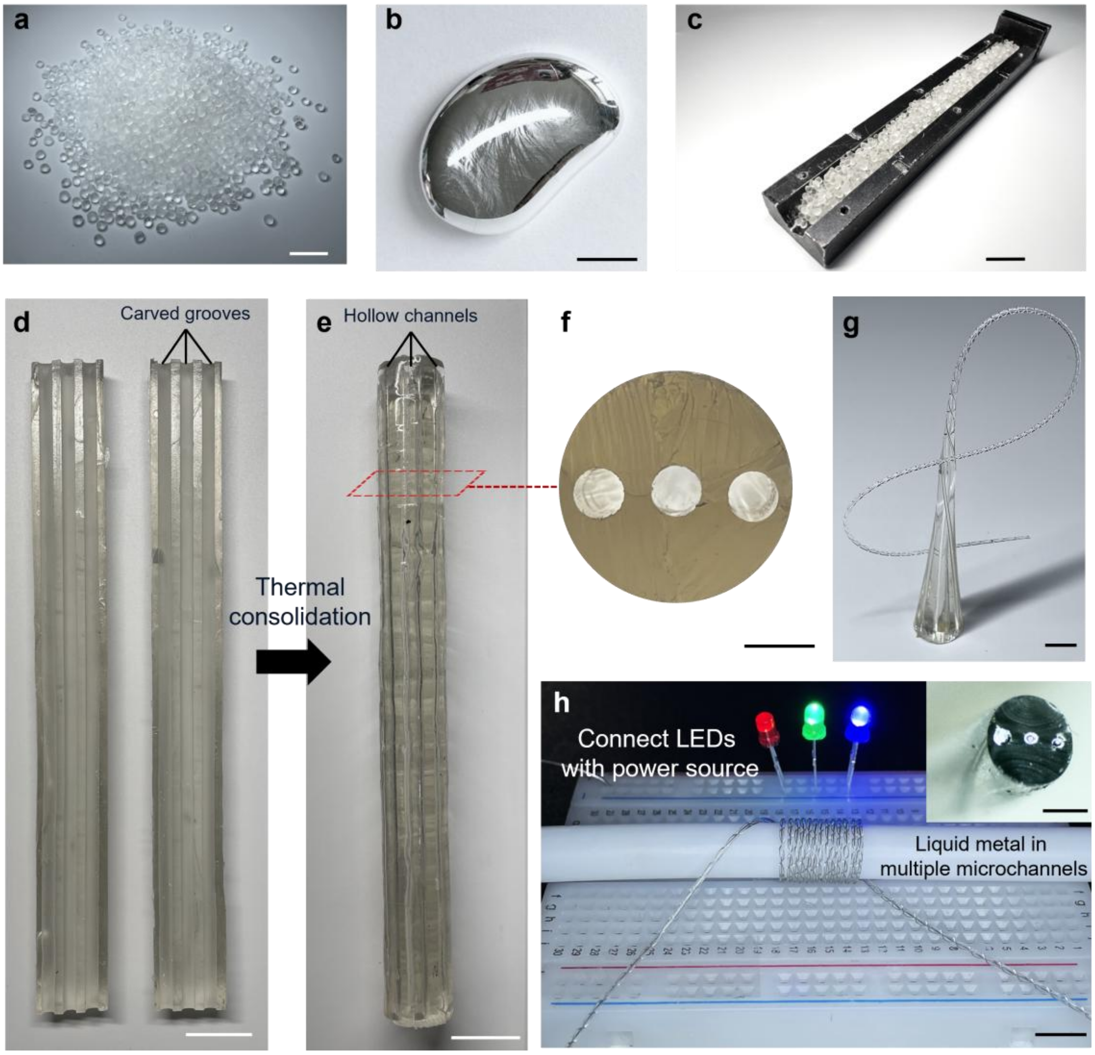

Supplementary Fig. 1 | Fabrication of the tri-channel fiber preform and photograph of the thermal drawing fiber. a, Photograph of SEBS particles (scale bar, 2 cm). **b,** Photograph of liquid metal (scale bar, 1 cm). **c,** The pellets are introduced into a custom-designed semicircular mold to produce semicircular blocks (scale bar, 2 cm). **d,e,** Grooves are carved into one side of the semicircular block, followed by thermal consolidation, resulting in the formation of a cylindrical preform containing three hollow channels (scale bar, 2 cm). **f,** Photograph of preform cross section (scale bar, 5 mm). **g,** Photograph of the tail end of the preform following thermal drawing (scale bar, 2 cm). **h,** By injecting liquid metal into the hollow microchannels of the thermally drawn fiber, conductive pathways are formed, enabling the fiber to illuminate LEDs (scale bar, 1cm). The insert shows the cross-section of stretchable fiber with multiple conductive microchannels.

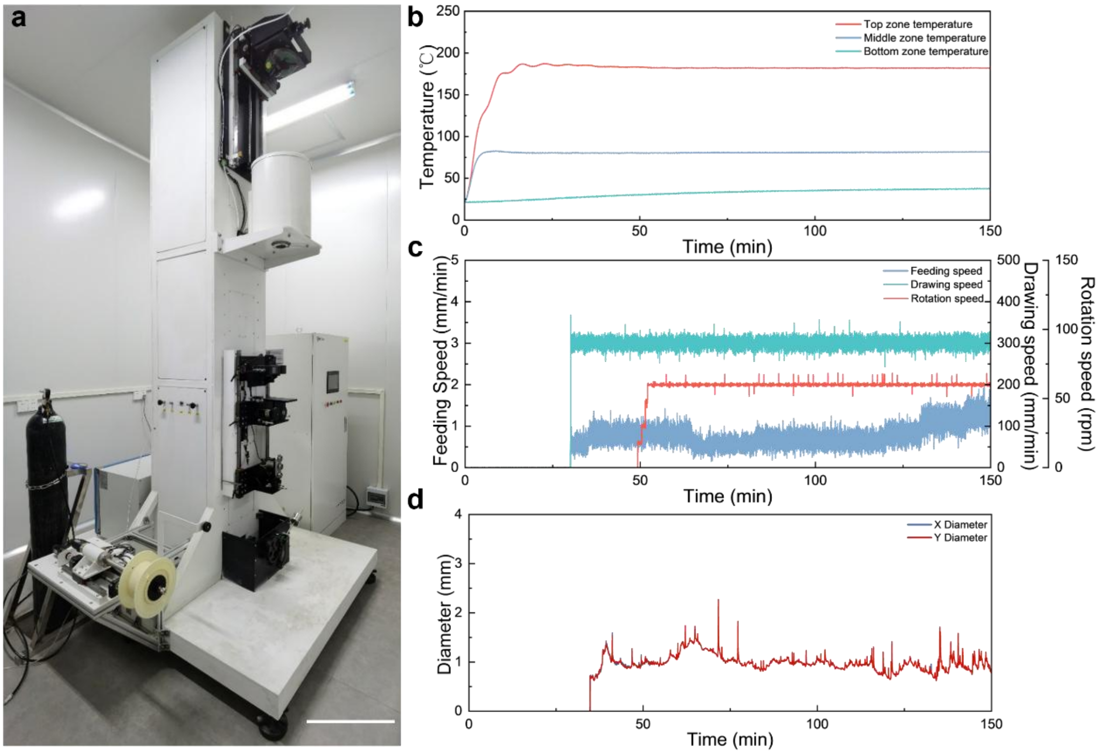

Supplementary Fig. 2 | Thermal drawing tower and drawing parameters. a, Photograph of thermal drawing tower (scale bar, 50 cm). **b,** Temperature settings employed during the thermal drawing process. **c,** Feeding speed, drawing speed and rotational speed settings during the thermal drawing process. **d,** Control of fiber diameter during the thermal drawing process. Source data are provided as a Source Data file.

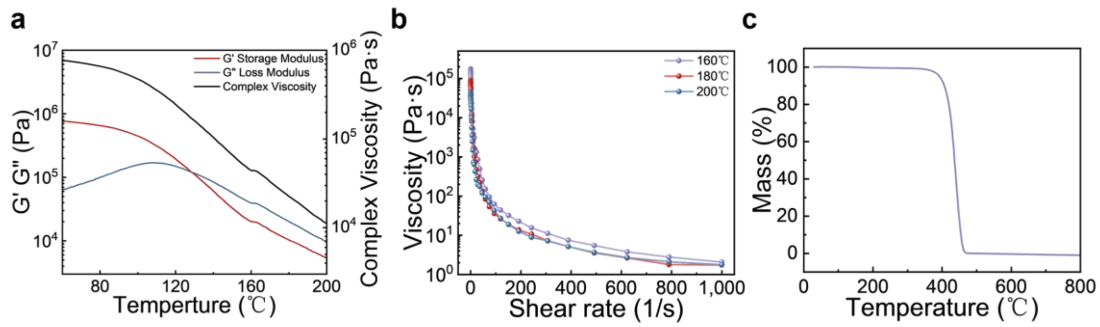

Supplementary Fig. 3 | Thermodynamic and rheological properties of SEBS. a, SEBS G1657 modulus & viscosity curve. **b,** Viscosity as a function of shear rate (1/s) for SEBS G1657 at different temperature. **c,** Thermogravimetric curves of SEBS. Source data are provided as a Source Data file.

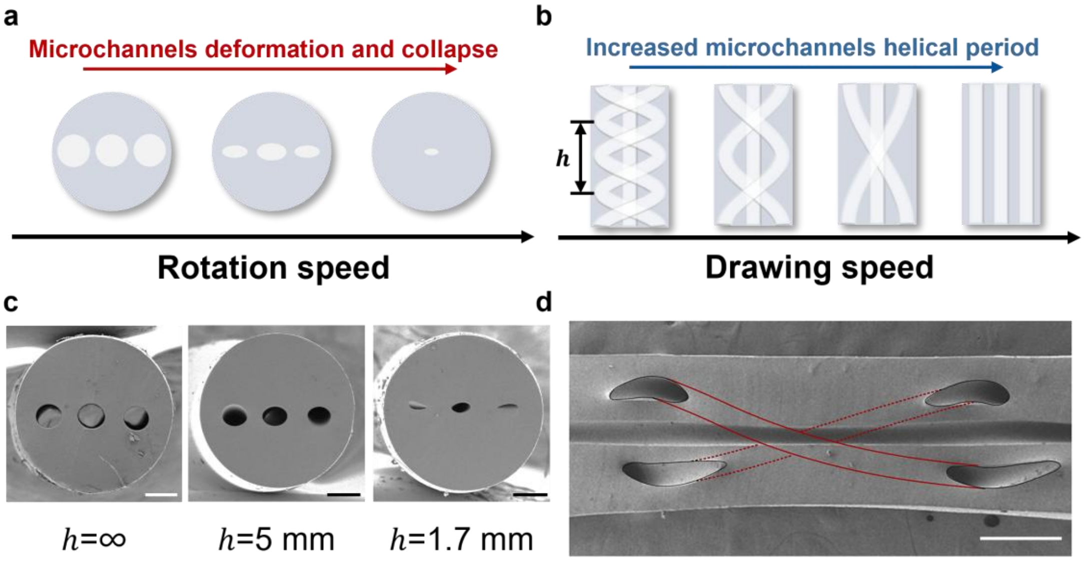

Supplementary Fig. 4 | Effect of drawing and rotation speed on the helical microchannels structure within the fiber during thermal drawing. a, The effect of rotation speed on microchannel structure. **b,** The effect of drawing speed on h of microchannels. **c,** SEM image of the cross-section of tri-channel fibers with varying h (scale bar, 200 μm). **d,** SEM image showing the longitudinal cross-section of the tri-channel fiber (scale bar, 500 μm). The micrographs shown in **c** and **d** are representative of three independent experiments that yielded similar results.

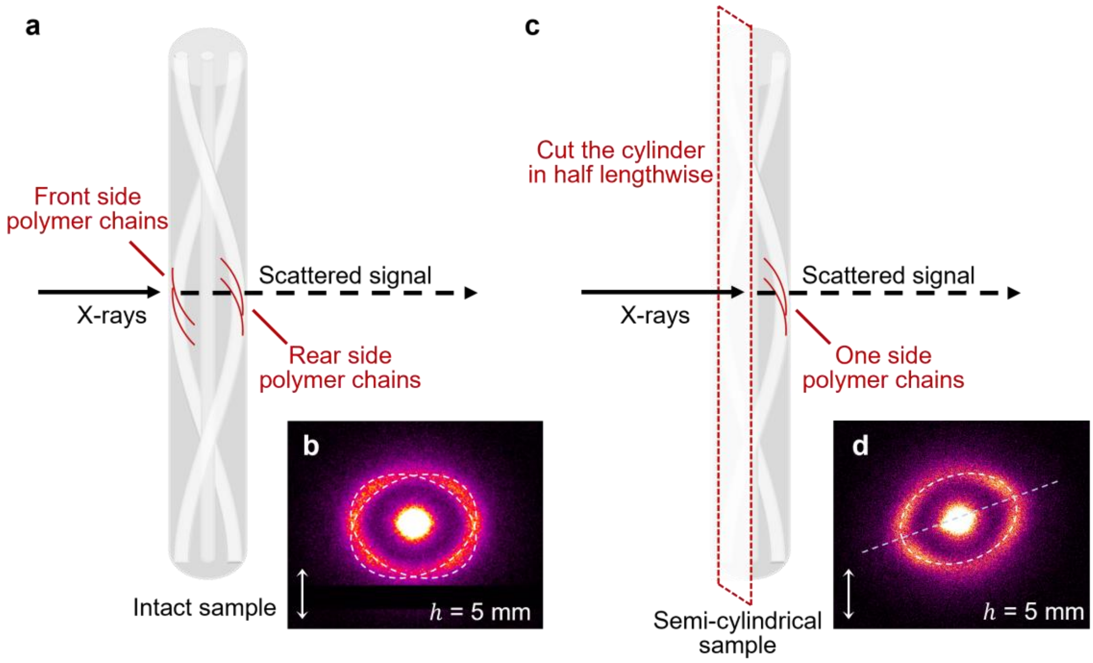

Supplementary Fig. 5 | Schematic diagrams and SAXS results of samples with different morphologies. a, SAXS measurements of the intact sample reveal a symmetric opposition in polymer chain orientation between the front and rear sides. **b,** The resulting SAXS pattern arises from the superposition of scattering signals originating from polymer chains on both the front and rear sides, with the arrow denoting the fiber axis direction. **c,** For the semi-cylindrical sample, SAXS interrogation is confined to polymer chains on one side. **d,** The SAXS pattern of the semi-cylindrical sample reflects only the orientation information of the polymer chains on one side.

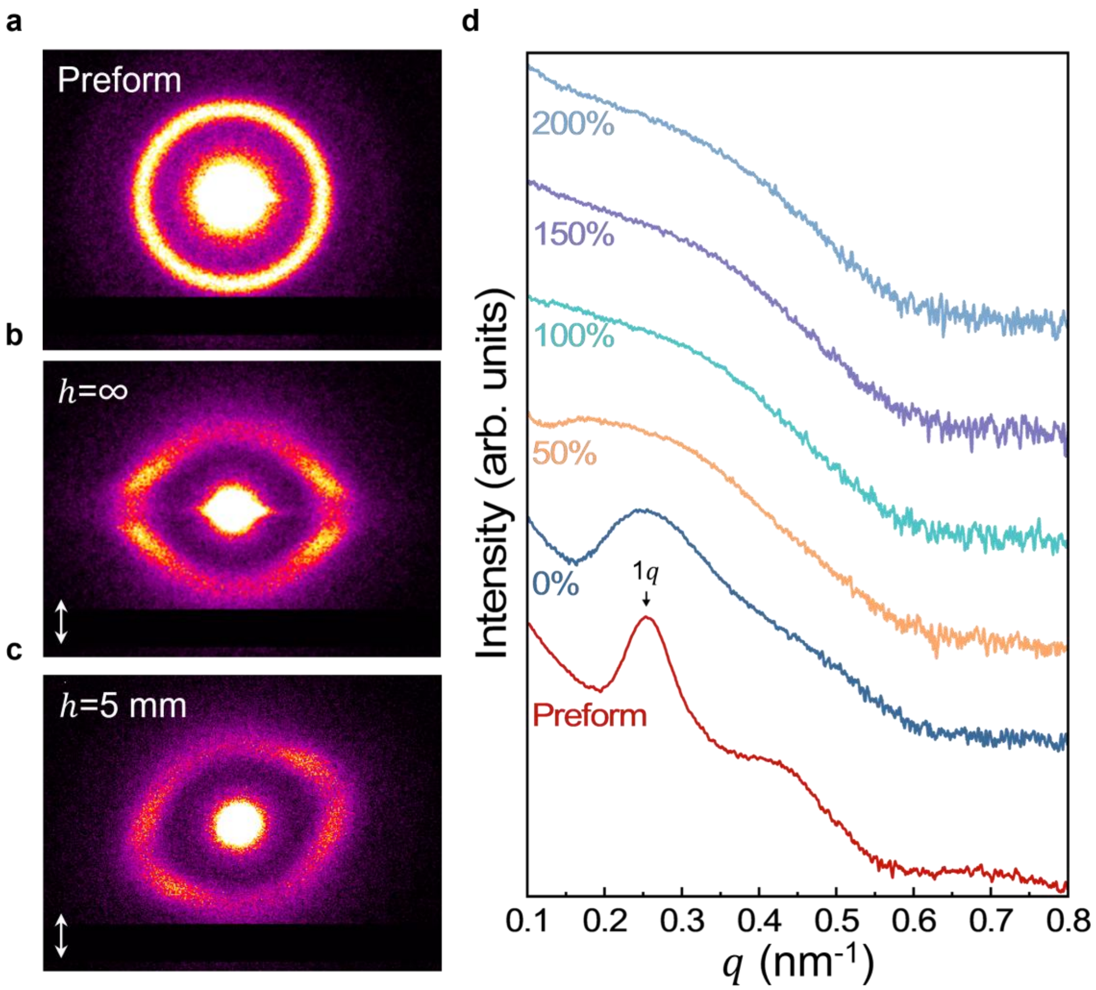

Supplementary Fig. 6 | SAXS properties of the preform and tri-channel fibers. a-c, The 2D SAXS patterns of preform (**a**), the tri-channel fiber with straight microchannels (**b**, corresponding to $h = \infty$), and helical microchannels (**c**, $h = 5$ mm), respectively. White arrows represent fiber length and stretching direction. **d,** 1D SAXS profiles of preform and fiber with helical microchannels ($h = 5$ mm) under different tensile strain. Source data are provided as a Source Data file.

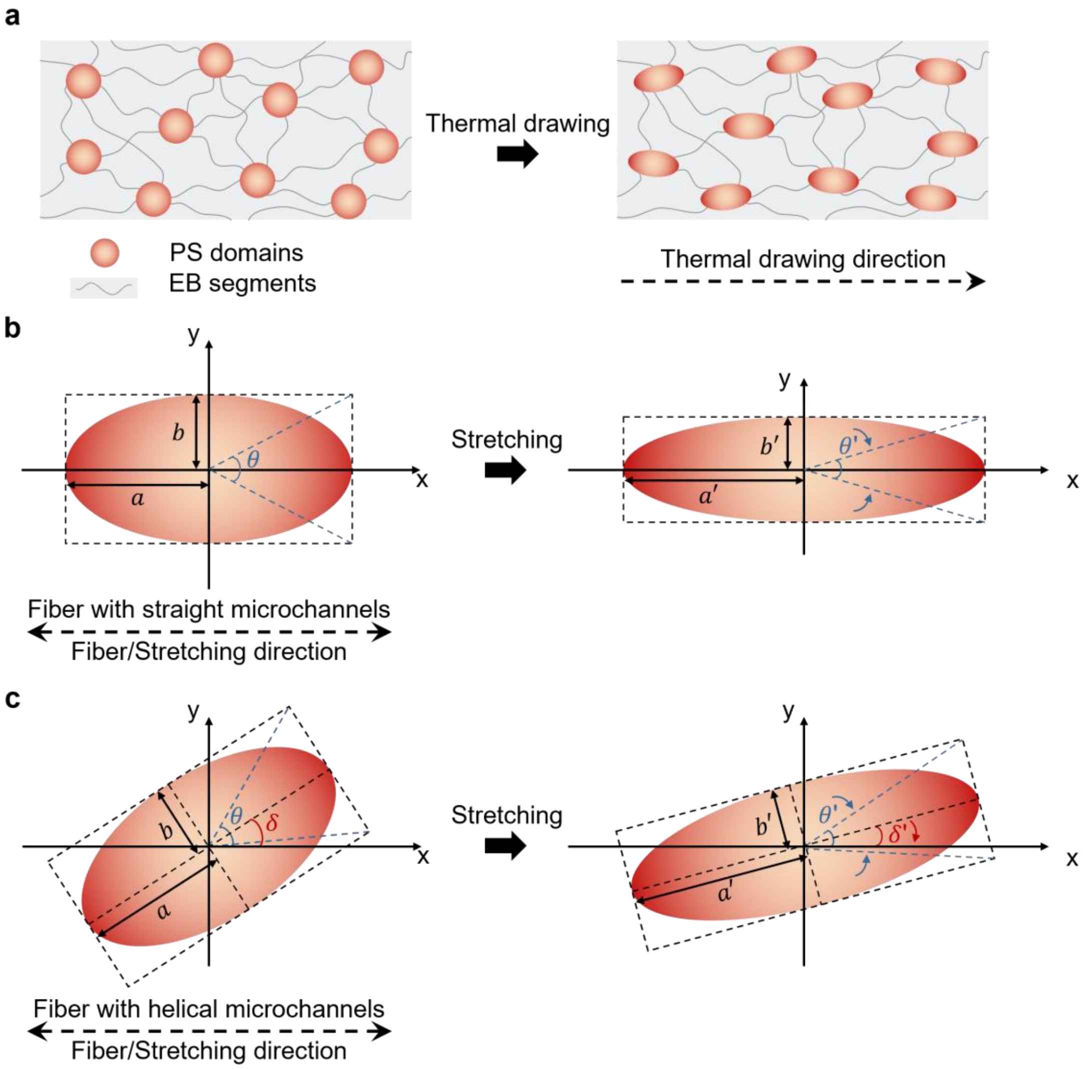

Supplementary Fig. 7 | Schematic illustration of structural evolution in PS domains during thermal drawing processing and mechanical loading. a, Morphology evolution of PS domains, illustrating the transition from spherical to ellipsoidal geometries during thermal drawing. **b,** Morphological changes of elliptical PS domains under tensile strain in the fiber with straight microchannels. **c,** Morphological changes of elliptical PS domains under tensile strain in the fiber with helical microchannels. where a and b represent the semi-major and semi-minor axes of the ellipse, respectively. θ denotes the orientation angle of the ellipse, and δ indicates the inclination angle between the major axis of the ellipse and the fiber axis. a', b', θ', δ' represent the semi-major axis, semi-minor axis, orientation angle, and inclination angle of the elliptical domain under fiber stretching, respectively.

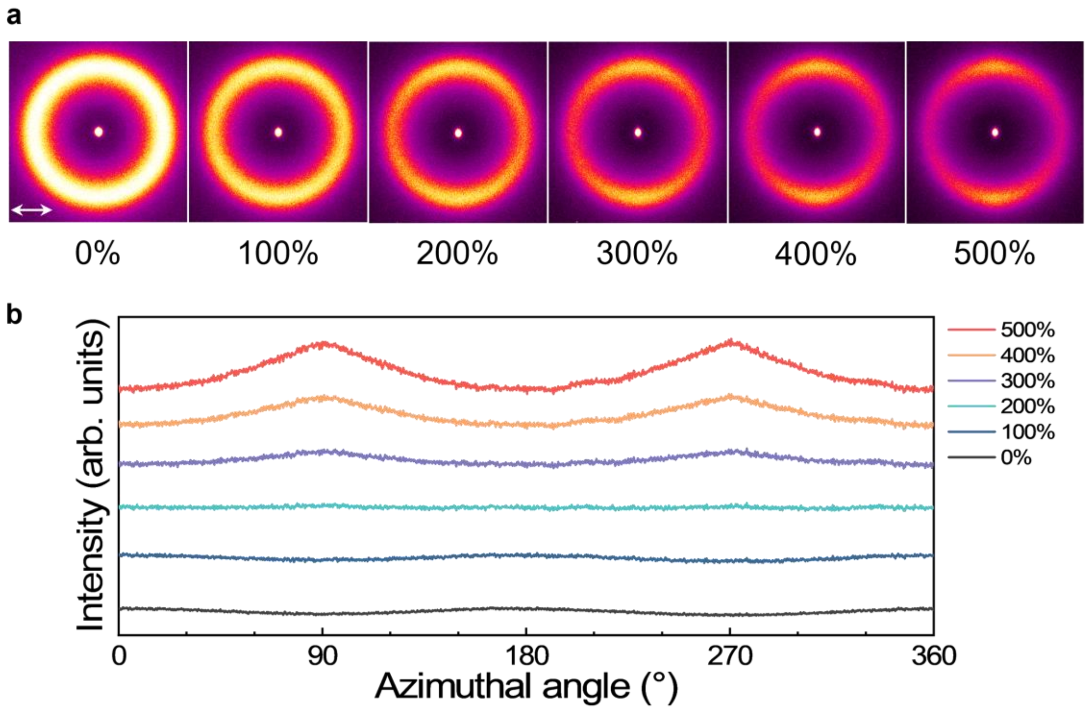

Supplementary Fig. 8 | WAXS properties of the tri-channel fiber under various tensile strain. a, 2D WAXS patterns of fiber under different tensile strain. The white arrow represents the fiber length and stretching direction. **b,** 1D scattering profiles obtained from the azimuthal angle integration of the 2D WAXS data under different tensile strain. Source data are provided as a Source Data file.

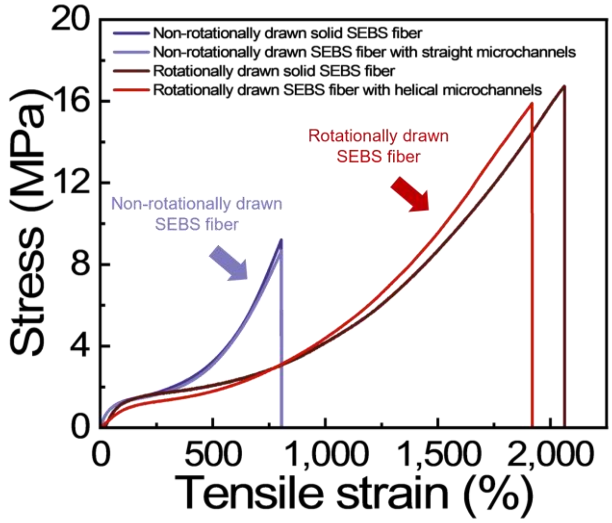

Supplementary Fig. 9 | Comparative mechanical properties of microchanneled fibers and their solid counterparts fabricated via rotational versus non-rotational drawing. The helical period (h) of the rotationally drawn fibers is fixed at 5mm. Source data are provided as a Source Data file.

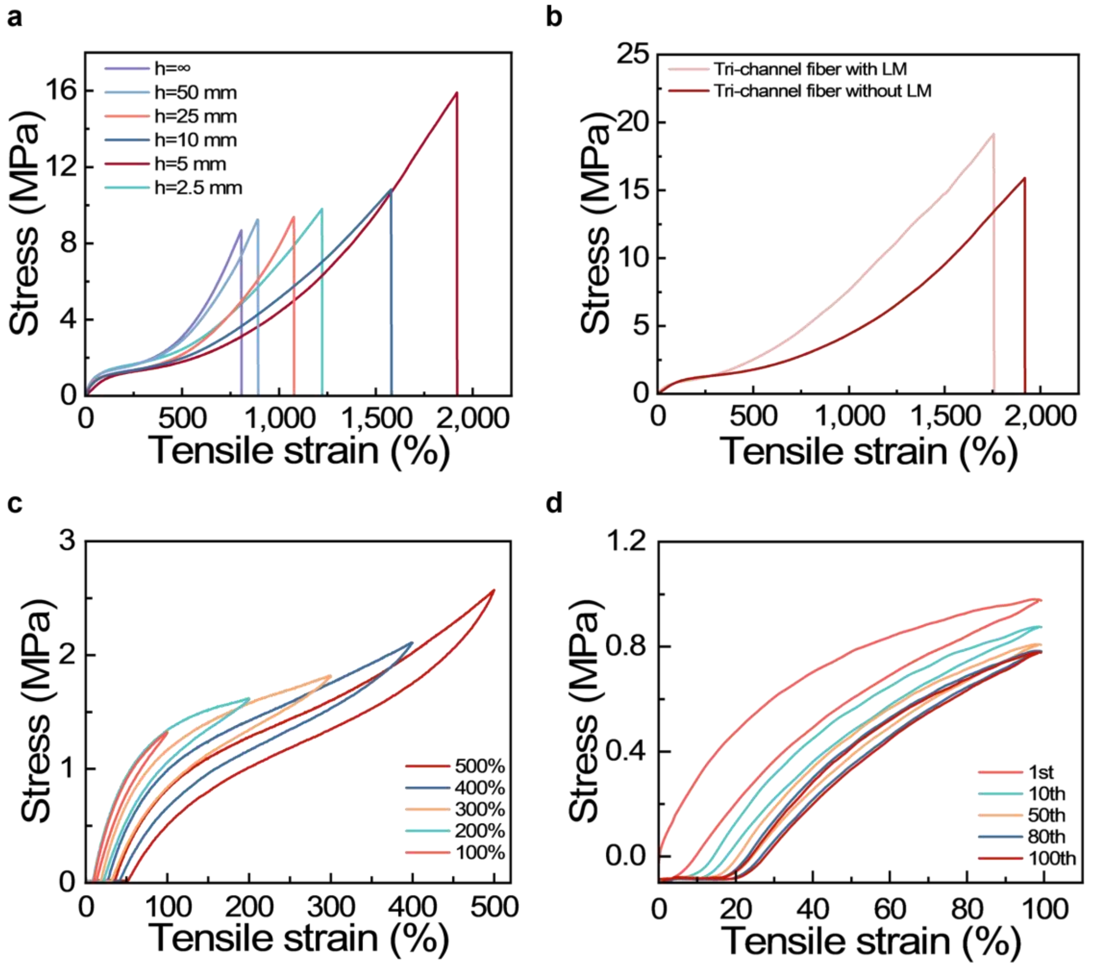

Supplementary Fig. 10 | Mechanical properties and mechanical cycle performance of the tri-channel fiber. a, Stress−strain curves for tri-channel fibers with different h. **b,** Comparison of stress–strain curves for tri-channel fibers (h=5 mm) with and without liquid metal filling the microchannels. **c,** Stress-strain curves of the fiber with helical microchannels (h = 5 mm) under various elongations. **d,** Cyclic loading-unloading curves of the fiber with helical microchannels (h = 5 mm) at a fixed strain of 100% over 100 cycles. Source data are provided as a Source Data file.

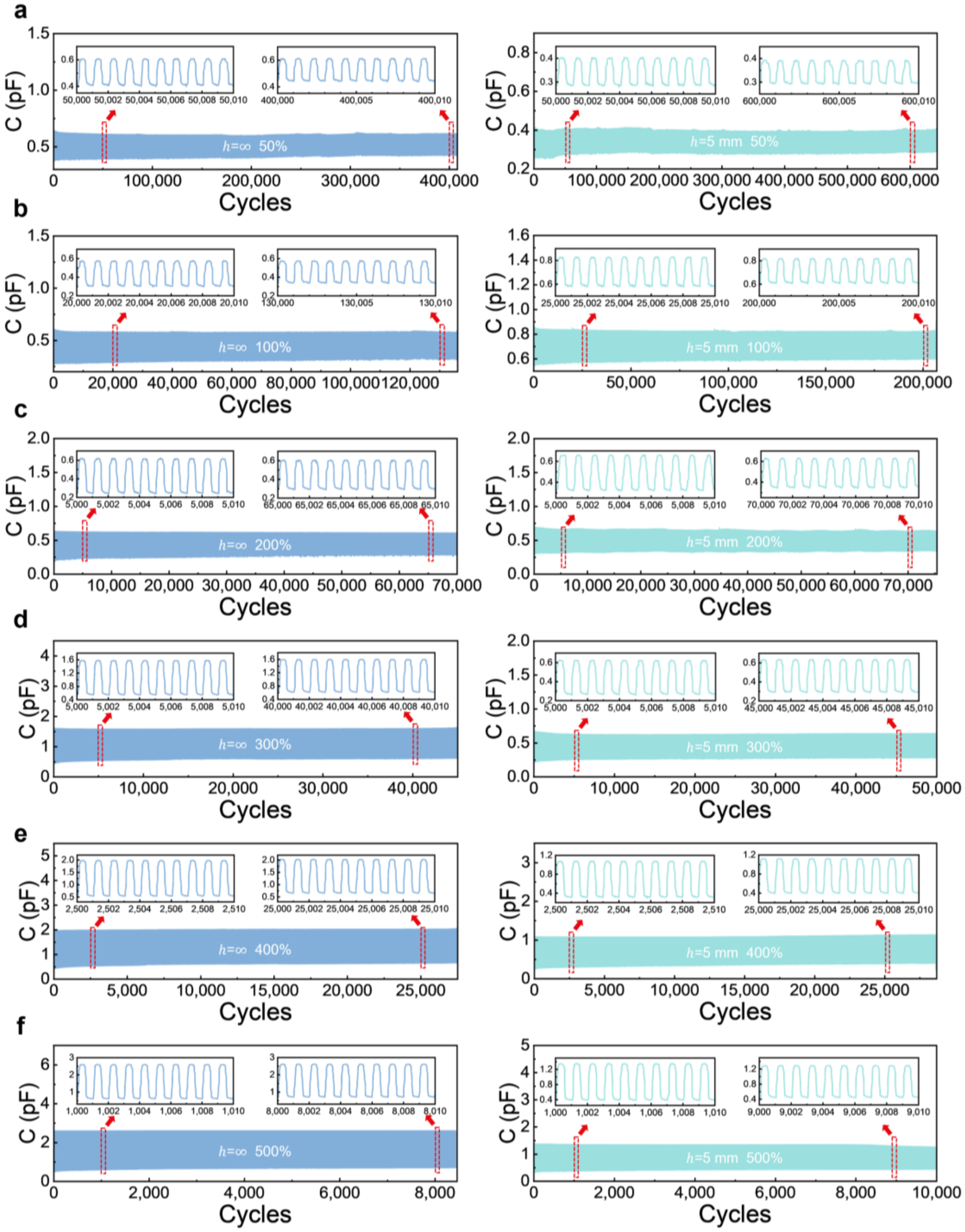

Supplementary Fig. 11 | Cyclic tensile durability of fibers with straight (*h*=∞, blue) versus helical (*h*=5 mm, cyan) microchannels. a, Under 50% cyclic tensile strain, maximum durability cycles reach 408,500 (*h*=∞) and 641,400 (*h*=5 mm). **b,** Under 100% cyclic tensile strain, maximum durability cycles reach 135,500 (*h*=∞) and 206,600 (*h*=5 mm). **c,** Under 200% cyclic tensile strain, maximum durability cycles reach 70,200 (*h*=∞) and 75,500 (*h*=5 mm). **d,** Under 300% cyclic tensile strain, maximum durability cycles reach 45,300 (*h*=∞) and 50,500 (*h*=5 mm). **e,** Under 400% cyclic tensile strain, maximum durability cycles reach 27,600 (*h*=∞) and 28,600 (*h*=5 mm). **f,** Under 500% cyclic tensile strain, maximum durability cycles reach 8,400 (*h*=∞) and 10,100 (*h*=5 mm). Source data are provided as a Source Data file.

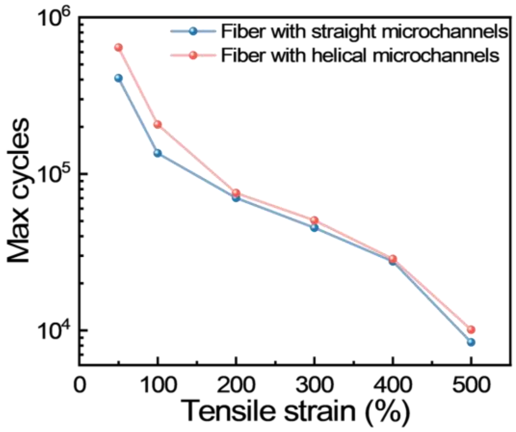

Supplementary Fig. 12 | Comparison of the maximum cycles of straight (h=∞, blue) and helical (h=5 mm, red) microchannels under different tensile strains. Source data are provided as a Source Data file.

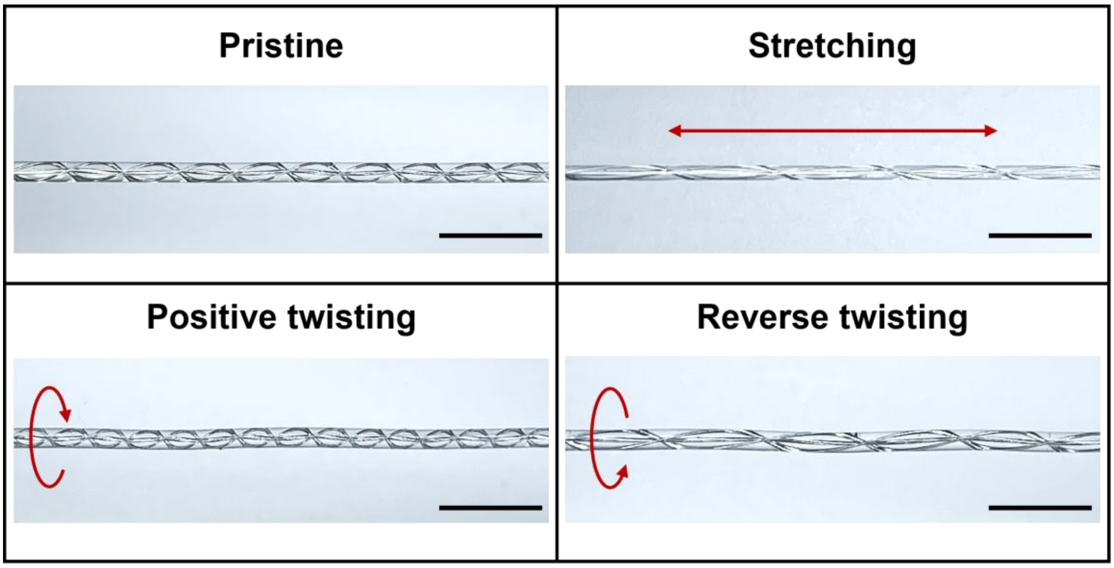

Supplementary Fig. 13 | Digital microscopic images of the dual-strain fiber sensor (h = 5 mm) under stretching and bidirectional twisting (Scale bar 5 mm). The digital microscopic images are representative of three independent experiments that yielded similar results.

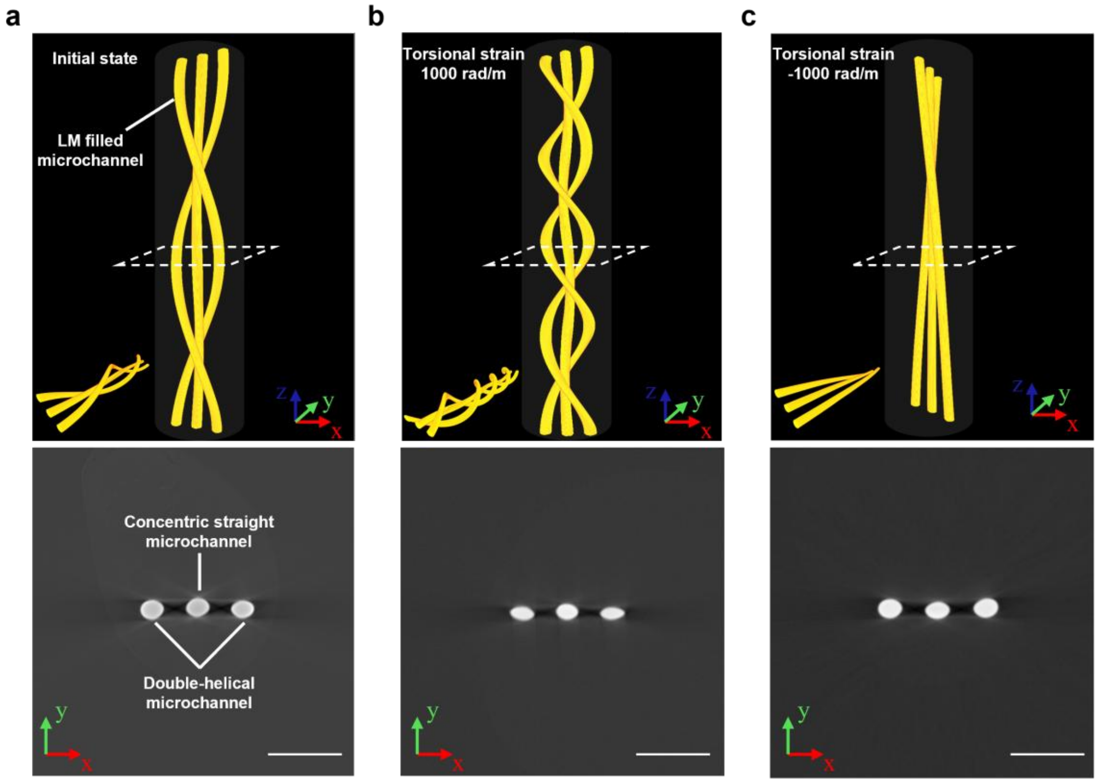

Supplementary Fig. 14 | 3D XRM reconstructions and cross-sectional CT slices of the tri-channel fiber under different torsional strains. a, 3D structural configuration and cross-sectional CT image of the fiber in its initial state. **b,** Structural configuration and cross-sectional CT image under a positive torsional strain of 1000 rad/m. **c,** Structural configuration and cross-sectional CT image under a reverse torsional strain of -1000 rad/m. Scale bar, 500 μm. The CT images are representative of three independent experiments that yielded similar results.

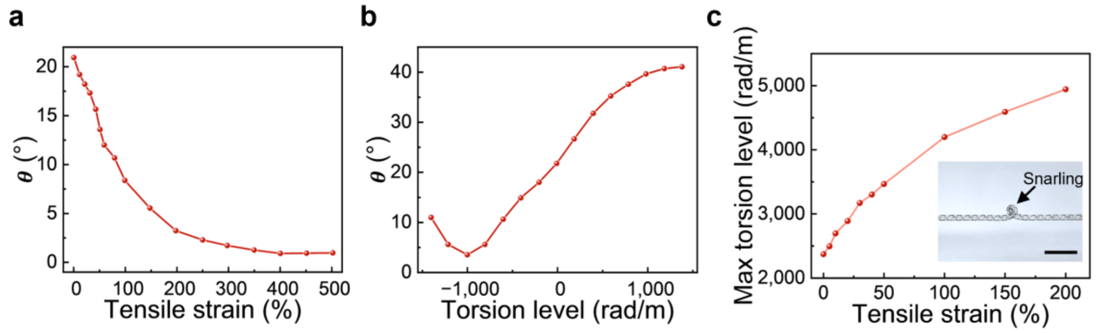

Supplementary Fig. 15 | Changes in the internal helical microchannels of the dual-strain fiber sensor under tensile and torsional strain. a, b, The response curve of the helix angle of the helical microchannel within fiber strain sensor (h = 5 mm) as a function of tensile strain and torsion level, respectively. **c,** The relationship between the maximum torsion level and the tensile strain of the fiber strain sensor. The insert shows a photograph of a highly twisted fiber strain sensor forming plectonemes at a snarling point (scale bar, 5 mm). Source data are provided as a Source Data file.

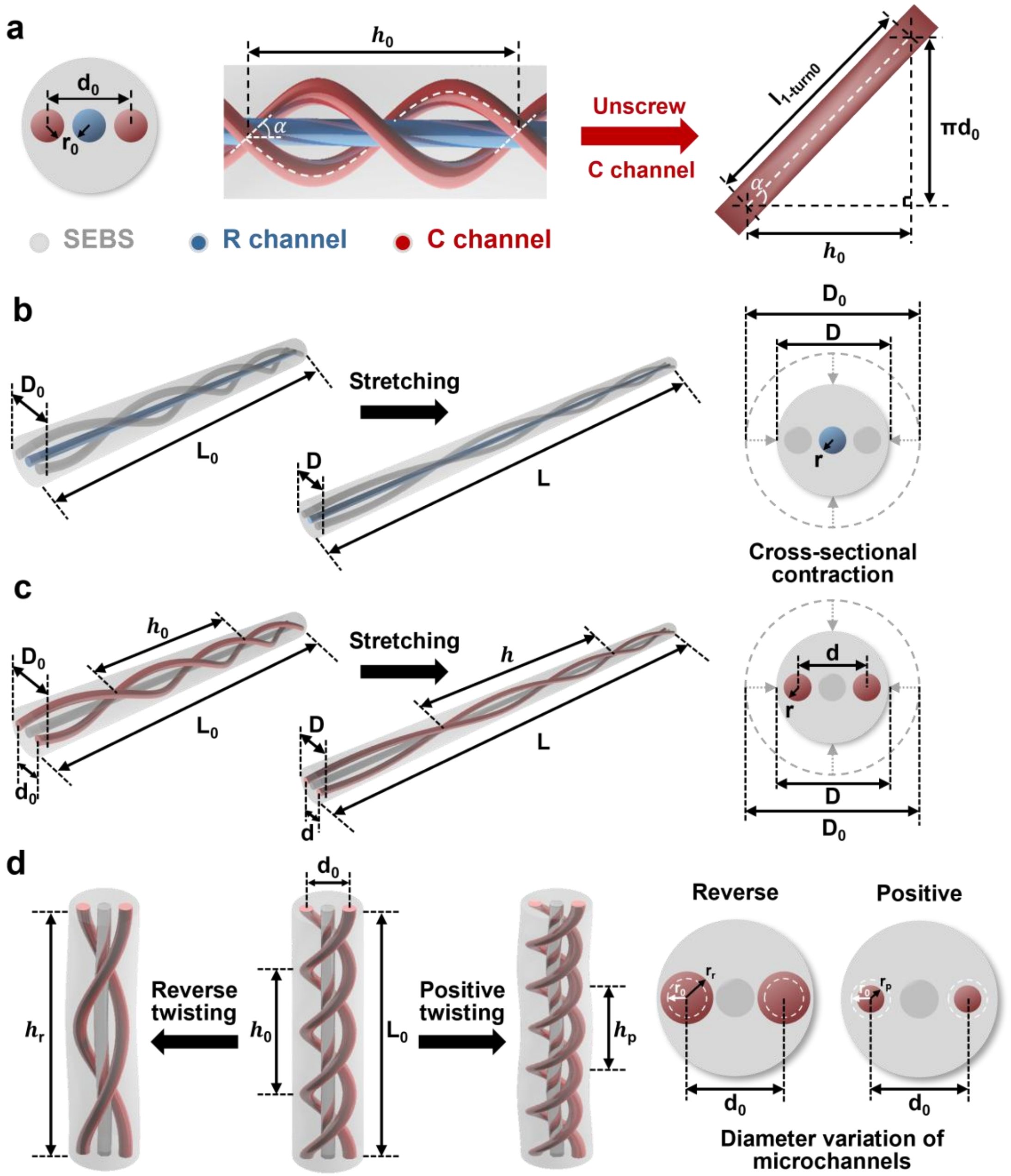

Supplementary Fig. 16 | Working mechanism of the dual-strain fiber sensor for tensile and torsional strain sensing. a, The cross-section and side diagrams of the fiber strain sensor. The white dashed in the helical capacitance channel within the fiber highlights a single period of the helical channel and its unscrew version. **b,** Schematic diagram of resistance channel variation under tensile strain. **c,** Schematic diagram of capacitance channel variation under tensile strain. **d,** Schematic diagram of capacitance channel under torsional strain.

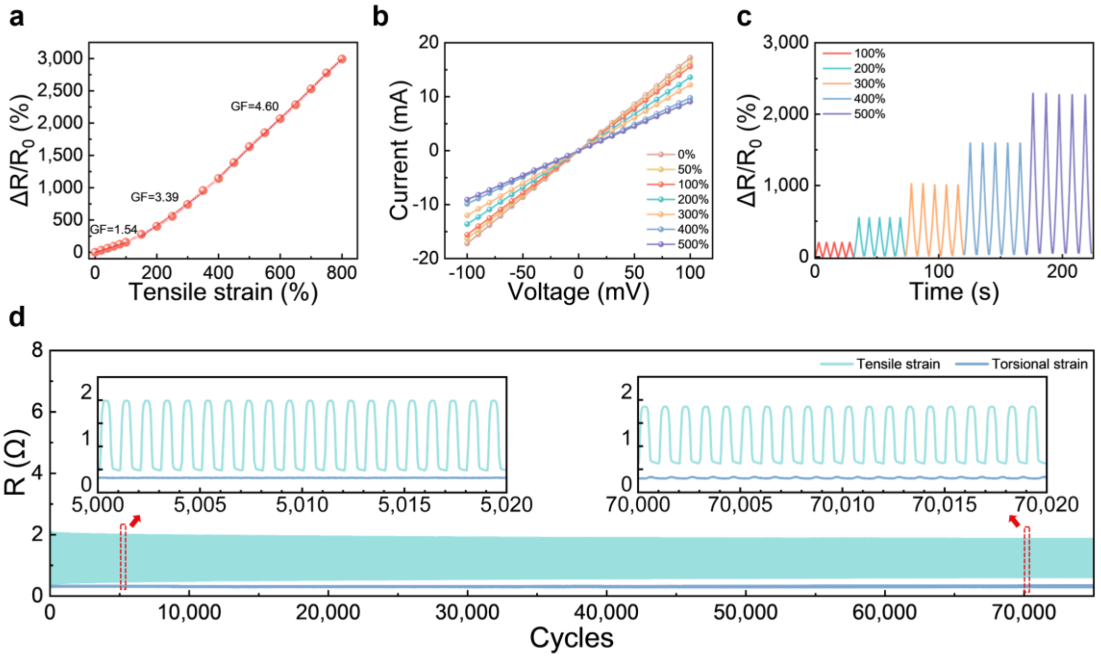

Supplementary Fig. 17 | Resistive electromechanical response characteristics of dual-strain fiber sensors. a, Resistive response of sensors under tensile strain. **b,** Volt-ampere characteristics curve of sensor under different tensile strains. **c,** Resistive response of the sensor under varying tensile strains (100-500%). **d,** Resistive response of the sensor under cyclic 200% tensile strain (cyan) and ±1,000 rad/m torsional strain (blue) over 75,000 cycles, respectively. The inset shows cycles from 5,000-5,020 and 70,000-70,020. Source data are provided as a Source Data file.

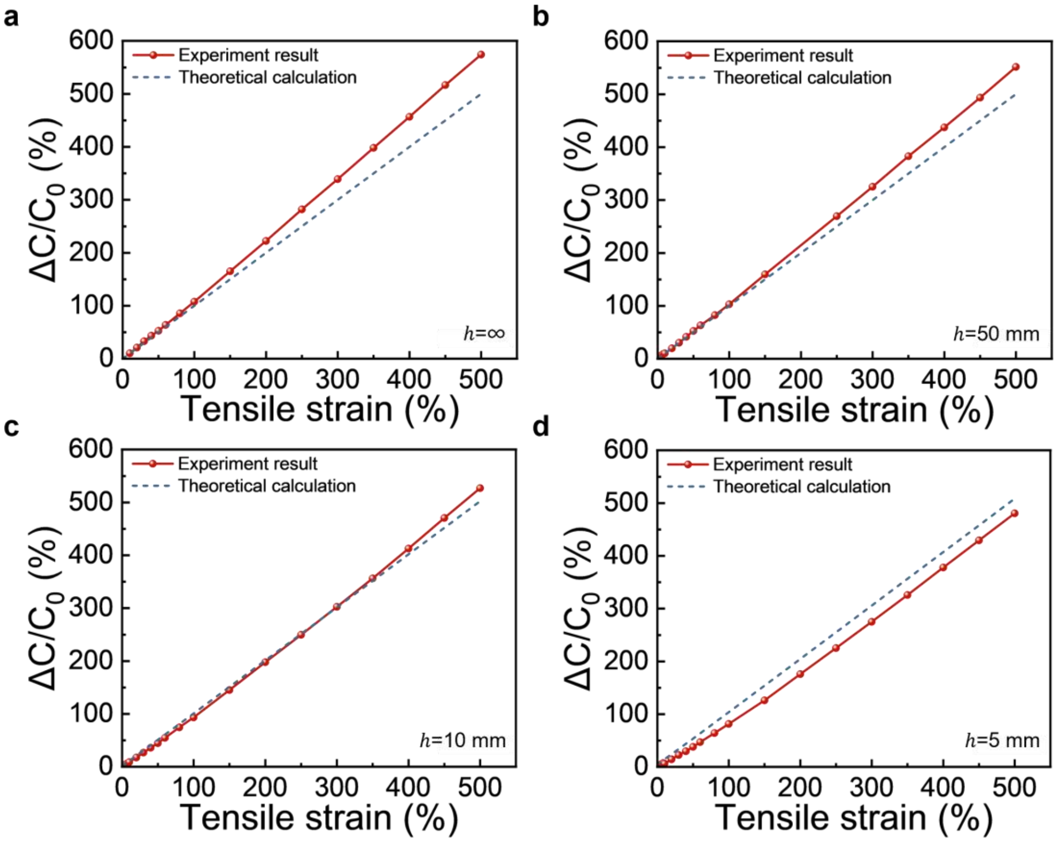

Supplementary Fig. 18 | Relative capacitance changes of the dual-strain fiber sensor with different h under tensile strain and theoretical calculation results. a, The stretchable fiber with straight microchannels. **b,** h = 50 mm. **c,** h = 10 mm. **d,** h = 5 mm. Source data are provided as a Source Data file.

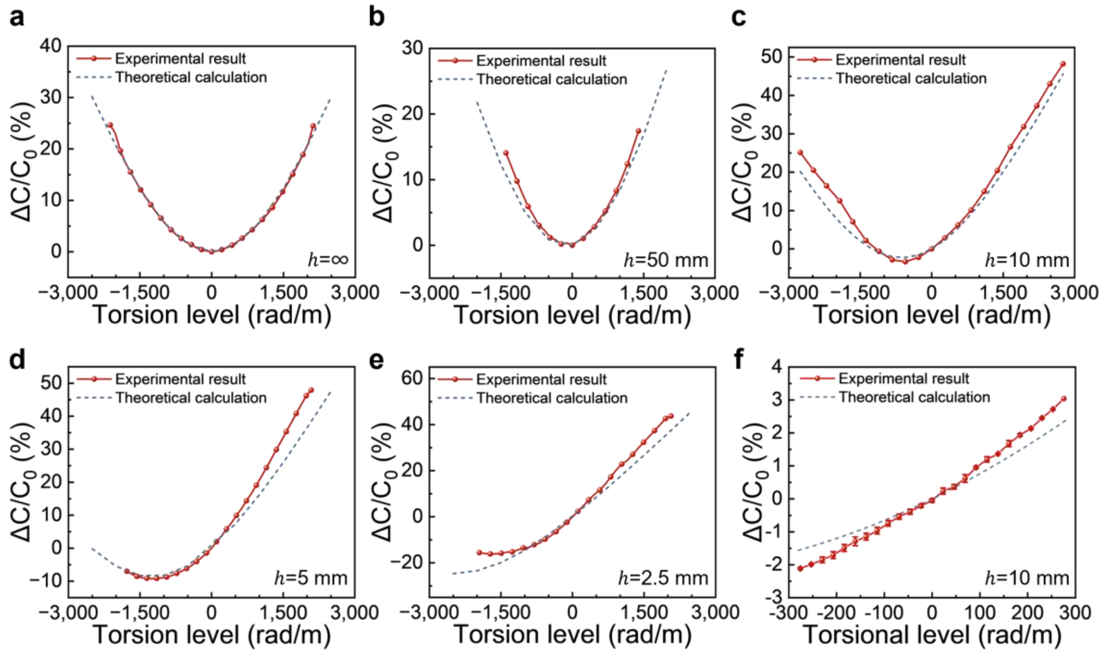

Supplementary Fig. 19 | Relative capacitance changes of the dual-strain fiber sensor with different h under torsional strain and theoretical calculation results. **a,** The stretchable fiber with straight microchannels. **b,** h = 50 mm. **c,** h = 10mm. **d,** h = 5 mm. **e,** h = 2.5 mm. **f,** Comparison between experimental result and theoretical calculation under ±300 rad/m (h = 10 mm). Source data are provided as a Source Data file.

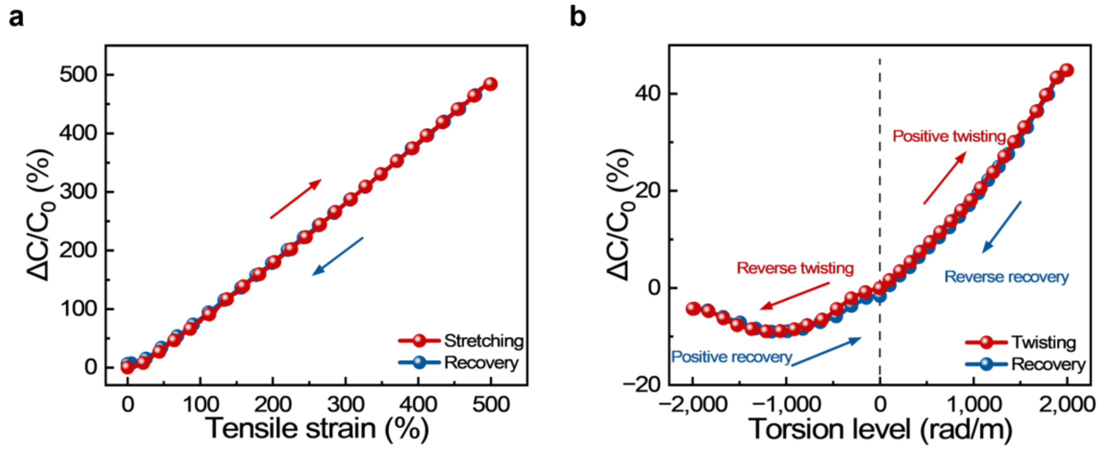

Supplementary Fig. 20 | Electrical hysteresis characteristics of fiber sensors. a, Electrical hysteresis of the fiber sensor under 500% tensile strain. **b,** Electrical hysteresis under torsional strain ranging from –2000 to 2000 rad/m. Source data are provided as a Source Data file.

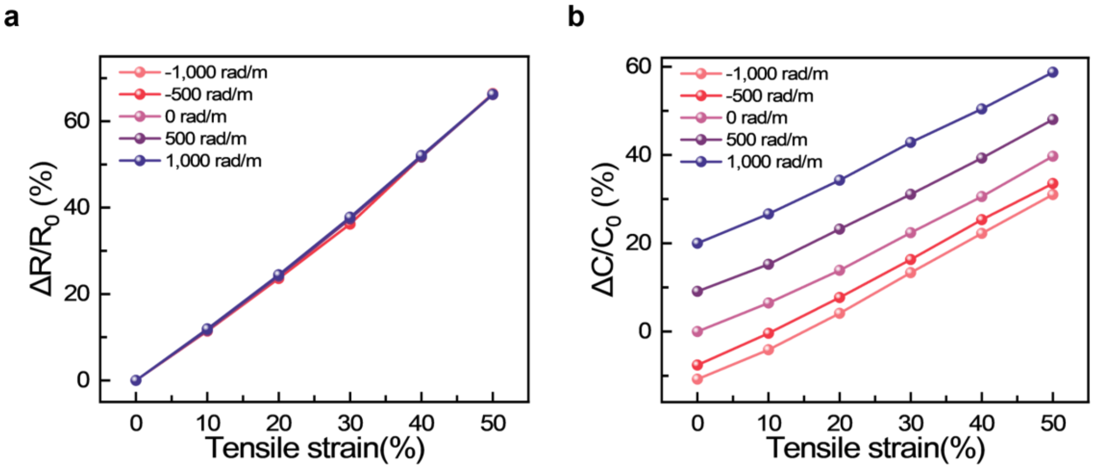

Supplementary Fig. 21 | Resistive and capacitive responses of the sensor to tensile strain under various fixed torsional strains. a, The sensor exhibits a consistent resistive response to tensile strain under various fixed torsional strain conditions. **b,** The sensor demonstrates a linear capacitive response to tensile strain under various fixed torsional strains. Source data are provided as a Source Data file.

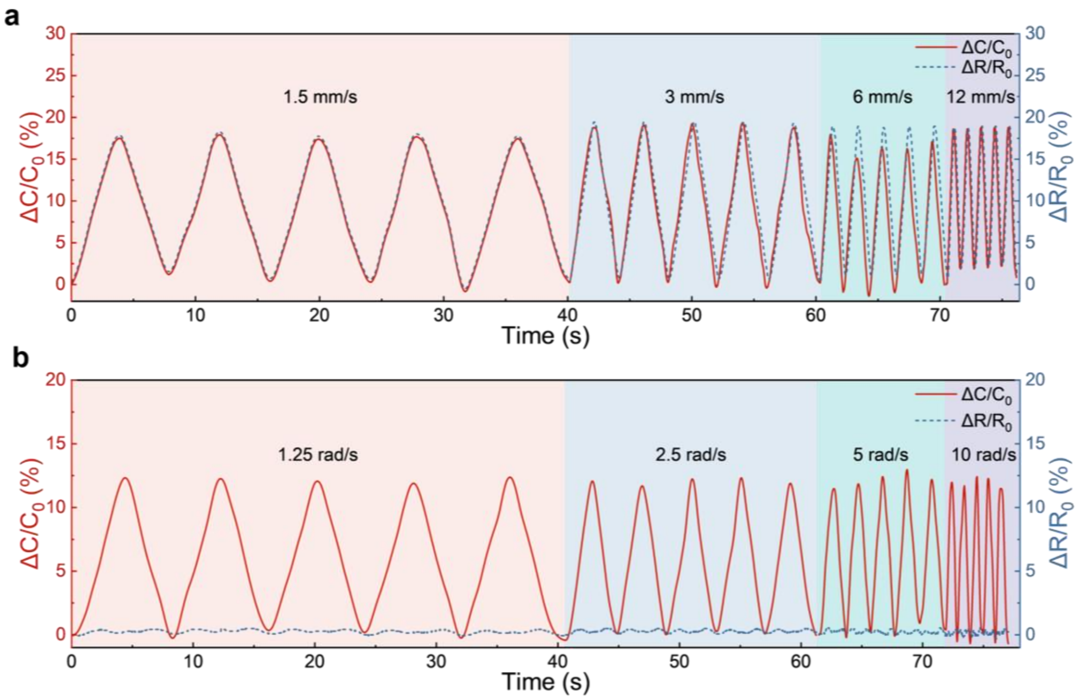

Supplementary Fig. 22 | Resistive and capacitive response curves of the dual-strain fiber sensor under tensile and torsional strains at various loading rates. a, Relative changes in resistance and capacitance of the fiber strain sensor under tensile strain at loading rates of 1.5, 3, 6, and 12 mm/s. **b,** Relative changes in resistance and capacitance of the fiber strain sensor under torsional strain at loading rates of 1.25, 2.5, 5, and 10 rad/s. Source data are provided as a Source Data file.

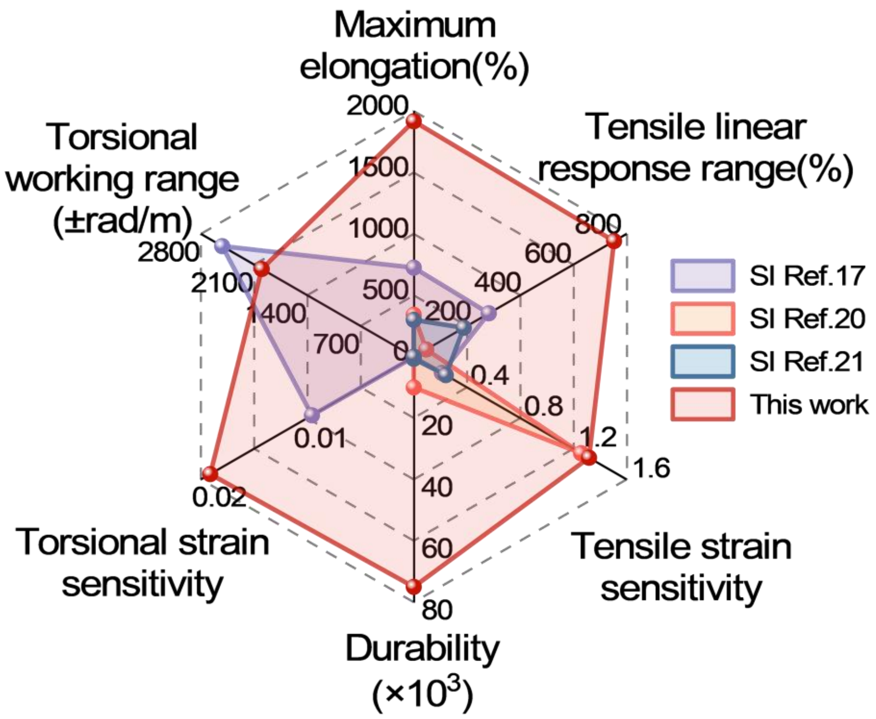

Supplementary Fig. 23 | Comparison of the performance of dual-strain fiber sensor with previously reported strain sensors. Source data are provided as a Source Data file.

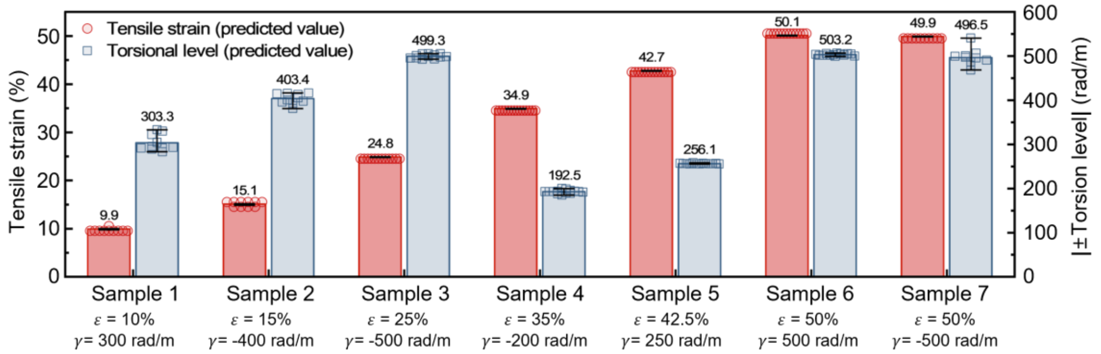

Supplementary Fig. 24 | Decoupling performance under composite strain combinations. Torsional strains are presented as absolute values. Values above the error bars represent the mean, and error bars indicate the standard deviation (n = 10 independent samples). Source data are provided as a Source Data file.

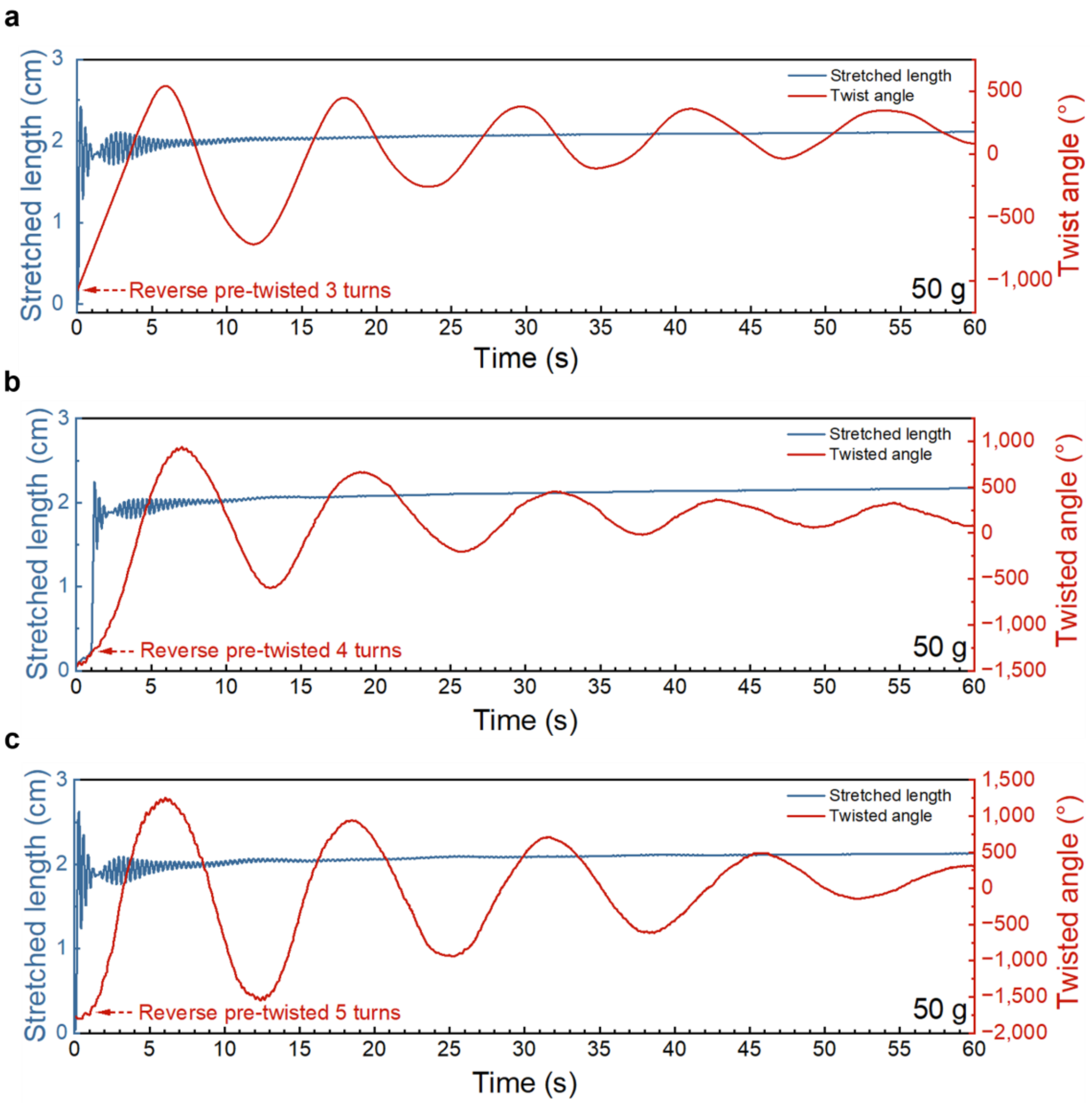

Supplementary Fig. 25 | Real-time decoupling performance under constant load and varying pre-twist turns. a-c, Decoupled information with pre-twist of 3 turns (**a**), 4 turns (**b**), and 5 turns (**c**). Source data are provided as a Source Data file.

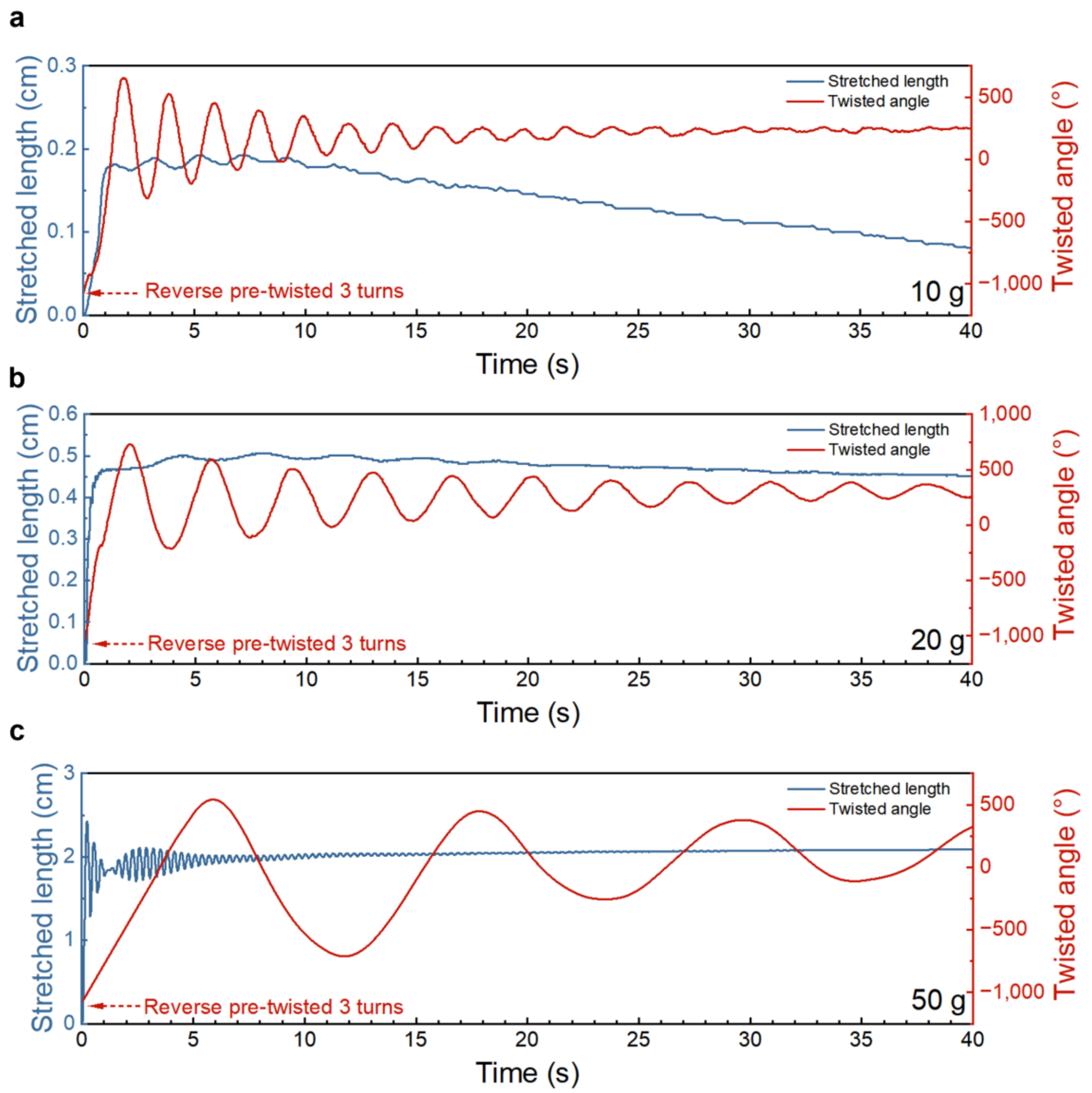

Supplementary Fig. 26 | Real-time decoupling performance under constant pre-twist turns and varying loads. a-c, Decoupled information under applied loads of 10 g (**a**), 20 g (**b**), and 50 g (**c**). Source data are provided as a Source Data file.

Supplementary Table 1 | The comparison of the sensing performance of the dual-strain fiber sensor with the reported literature of flexible strain sensors.

Ref.	Detectable strain types	Tensile linear response range	Tensile strain sensitivity	Maximum elongation	Torsional response range	Torsional strain sensitivity	Durability (Testing conditions)	Individual simultaneous monitoring ability
12	Tensile	15~27.5%	12	<50%	/	/	2,000 (ε=10%)	No
13	Tensile	0~50%	9.1×10^{6}	800%	/	/	2,000 (ε=40%)	No
14	Tensile/ Compression	0~20%	0.02	45%	/	/	10,000 (ε=15%)	Yes
15	Tensile/Torsion	0~80%	0.0207 mV/%	/	0~79 rad/m	0.0151 mV/%	>4,000 (ε=20%)	Yes
16	Tensile/Torsion	0~100%	0.66~0.82	/	0~10,887 rad/m	$(5.10 \pm 0.11)\times10^{-4}$ pF/cm^{2}	6 (γ=0~600 rad/m)	No
17	Tensile/Torsion /Compression	0~300%	0.22	720%	-2,520~2,520 rad/m	0.011 (CW)<br>0.004 (CCW)	5,300 (ε=200%)<br>5,100 (γ=2,520 rad/m)	No
18	Tensile/Torsion /Bending	0~1,000 $\mu\varepsilon$	-2.6 nm/mε	/	-180°~180°	183.85 nm/(rad/mm)	/	No
19	Tensile/Torsion /Bending	0~0.6%	-17.4	/	0~270°	/	1,000 (ε=0.8%)	No
20	Tensile/Bending /Compression	0~50%	1.26	341.7%	/	/	10,000 (ε=200%)	No
21	Tensile/ Torsion /Bending/Compression	0~200%	-0.25	/	/	/	700 (ε=100%)	No
**This work**	**Tensile/Torsion**	**0~800%**	**1.32**	**1,918%**	**-2,000~2,000 rad/m**	**0.022**	**75,000 (ε=200%)**<br>**425,000 (γ=±1,000 rad/m)**	**Yes**

ε: Tensile strain applied in cyclic testing. γ: Torsion level applied in cyclic testing. CW: Clockwise. CCW: Counterclockwise.

Supplementary Table 2 | SAXS/WAXS sample details, data collection, data analysis, and software used.

**(1) Sample details**	Sample 1 (Preform)	Sample 2 ($h = \infty$, straight)	Sample 3 (*h*=5 mm, helical)
**Source**	SEBS (Kraton G1657)	SEBS (Kraton G1657)	SEBS (Kraton G1657)
**Description**	SEBS preform	Tri-channel fiber with straight microchannels	Tri-channel fiber with helical microchannels (*h*=5 mm)

**(2) SAXS/WAXS data collection parameters**	
**Source, instrument**	GeniX3D Cu beamline (Xenocs, Xeuss 3.0 UHR)
**Wavelength (Å)**	1.54 Å (Cu Kα)
**Beam geometry**	50 kV, 600 μA; Sample-to-detector distance: SAXS=1,500 mm; WAXS=60 mm
***q* measurement range (nm^{-1})**	SAXS: 0.1-1 nm^{-1}; WAXS: 0.02-20nm^{-1}
**Exposure time**	300 s
**Sample configuration**	Fibers longitudinally sectioned into semi-cylindrical specimens for unambiguous orientation determination
**Sample temperature (°C)**	Room temperature

**(3) Software employed**	
**Data processing**	Fit2D converts 2D data to 1D patterns, yielding integrated intensity vs. scattering vector (q) and azimuthal angle (Ψ)

Supplementary References

1 Qu, Y., Nguyen-Dang, T., Page, A. G., Yan, W., Das Gupta, T., Rotaru, G. M., et al. Superelastic Multimaterial Electronic and Photonic Fibers and Devices via Thermal Drawing. *Advanced Materials* **30**, 1707251 (2018).

2 Xu, B., Ma, S., Xiang, Y., Zhang, J., Zhu, M., Wei, L., et al. In-Fiber Structured Particles and Filament Arrays from the Perspective of Fluid Instabilities. *Advanced Fiber Materials* **2**, 1-12 (2020).

3 Richard, I., Maurya, A. K., Shadman, S., Masquelier, E., Marthey, L. S., Neels, A., et al. Unraveling the Influence of Thermal Drawing Parameters on the Microstructure and Thermo–Mechanical Properties of Multimaterial Fibers. *Small* **18**, 2101392 (2021).

4 Weissenberg, K. A Continuum Theory of Rhelogical Phenomena. *Nature* **159**, 310-311 (1947).

5 Zhang, S., Yang, Q., Li, C., Fu, Y., Zhang, H., Ye, Z., et al. Solid-state cooling by elastocaloric polymer with uniform chain-lengths. *Nature Communications* **13**, 9 (2022).

6 Laperrousaz, S., Chen, X., Cleusix, M., Jourdan, L., Tribolet, L. & Sorin, F. Electronic fibres via the thermal drawing of liquid-metal-embedded elastomers. *Nature Electronics* **8**, 1072-1081 (2025).

7 Yu, R., Wu, L., Yang, Z., Wu, J., Chen, H., Pan, S., et al. Dynamic Liquid Metal–Microfiber Interlocking Enables Highly Conductive and Strain-insensitive Metastructured Fibers for Wearable Electronics. *Advanced Materials* **37**, 2415268 (2024).

8 Guo, S., Zhu, S., Qiao, Y., Feng, S., Yang, X., Kang, B., et al. Ultra-Low Hysteresis Under Large Deformation Enabled by Fast Chains Relaxation in Highly Competitive Dynamic Hydrogen Bond Networks. *Advanced Science* **12**, e05417 (2025).

9 Halliday, D., Resnick, R. & Walker, J. *Fundamentals of physics*. (John Wiley & Sons, 2013).

10 Lin, S., Yang, W., Zhu, X., Lan, Y., Li, K., Zhang, Q., et al. Triboelectric micro-flexure-sensitive fiber electronics. *Nature Communications* **15**, 2374 (2024).

11 Greaves, G. N., Greer, A. L., Lakes, R. S. & Rouxel, T. Poisson's ratio and modern materials. *Nature Materials* **10**, 823-837 (2011).

12 Lee, J., Ihle, S. J., Pellegrino, G. S., Kim, H., Yea, J., Jeon, C.-Y., et al. Stretchable and suturable fibre sensors for wireless monitoring of connective tissue strain. *Nature Electronics* **4**, 291-301 (2021).

13 Xue, F., Peng, Q., Ding, R., Li, P., Zhao, X., Zheng, H., et al. Ultra-sensitive, highly linear, and hysteresis-free strain sensors enabled by gradient stiffness sliding strategy. *npj Flexible Electronics* **8**, 14 (2024).

14 Liu, Z., Hu, X., Bo, R., Yang, Y., Cheng, X., Pang, W., et al. A three-dimensionally architected electronic skin mimicking human mechanosensation. *Science* **384**, 987-994 (2024).

15 Lee, J.-H., Chee, P.-S., Lim, E.-H., Low, J.-H. & Nguyen, N.-T. A Stretchable Kirigami-Inspired Self-Powered Electroactive Sensor for Tensile Strain and Torsion Sensing. *Advanced Engineering Materials* **24**, 2100961 (2021).

16 Cooper, C. B., Arutselvan, K., Liu, Y., Armstrong, D., Lin, Y., Khan, M. R., et al. Stretchable Capacitive Sensors of Torsion, Strain, and Touch Using Double Helix Liquid Metal Fibers. *Advanced Functional Materials* **27**, 1605630 (2017).

17 Son, W., Lee, J. M., Choi, J. H., Kim, J., Noh, J., Oh, M., et al. Double-Helical Carbon Nanotube-Wrapped Elastomeric Mandrel for Electrical Shortage-Free, One-Body Multifunctional Fiber Systems. *Advanced Functional Materials* **34**, 2312033 (2024).

18 Ma, C., Wang, D., Wang, J., Zhu, L., Bao, X. & Yuan, L. A Compact Sensor Capable of Temperature, Strain, Torsion and Curvature Measuring. *Journal of Lightwave Technology* **40**, 4896-4902 (2022).

19 Bu, Y., Shen, T., Yang, W., Yang, S., Zhao, Y., Liu, H., et al. Ultrasensitive strain sensor based on superhydrophobic microcracked conductive Ti3C2T MXene/paper for human-motion monitoring and E-skin. *Science Bulletin* **66**, 1849-1857 (2021).

20 Cao, P., Wang, Y., Yang, J., Niu, S., Pan, X., Lu, W., et al. Scalable Layered Heterogeneous Hydrogel Fibers with Strain-Induced Crystallization for Tough, Resilient, and Highly Conductive Soft Bioelectronics. *Advanced Materials* **36**, 2409632 (2024).

21 Huang, X., Liu, L., Lin, Y. H., Feng, R., Shen, Y., Chang, Y., et al. High-stretchability and low-hysteresis strain sensors using origami-inspired 3D mesostructures. *Science Advances* **9**, eadh9799 (2023).
