# Supplementary material for: A highly stretchable tri-channel fiber for composite motion decoupling
Source: Nat Commun. 2026 Jun 12;17:7465. doi: 10.1038/s41467-026-73959-4 (PMC13408763; doi:10.1038/s41467-026-73959-4)
Supplement: Supplementary file 2 — Description of Additional Supplementary File [file 41467_2026_73959_MOESM2_ESM.pdf]

The Description of Additional Supplementary Files

Supplementary Movie 1: Dual-strain fiber sensor for visual monitoring and decoupling composite motion states.
