# Supplementary material for: A highly stretchable tri-channel fiber for composite motion decoupling
Source: Nat Commun. 2026 Jun 12;17:7465. doi: 10.1038/s41467-026-73959-4 (PMC13408763; doi:10.1038/s41467-026-73959-4)
Supplement: Supplementary file 5 — Transparent Peer Review file [file 41467_2026_73959_MOESM5_ESM.pdf]

nature portfolio

Peer Review File

A highly stretchable tri-channel fiber for composite motion decoupling

Corresponding Author: Professor Chong Hou

This file contains all reviewer reports in order by version, followed by all author rebuttals in order by version.

Version 0:

Reviewer comments:

Reviewer #1

(Remarks to the Author)
This work presents a method of creating a tri-channel hollow fiber that is injected with liquid metal that can be used as a strain sensor. It uses a combination of resistance and capacitance measurements to determine the axial and torsional strain state. It provides a thorough characterization of its electromechanical properties and demonstrates its use as a sensor. The manuscript is coherently written and is recommended for publication after the following revisions are made.

1. There has been prior work on fabrication and characterization of liquid metal injected SEBS fibers (https://doi.org/10.1002/adfm.201202405) Such fibers have been used to make helical structures for capacitive sensing including torsional measurements. I certainly think it is worth mentioning.

C. B. Cooper, K. Arutselvan, Y. Liu, D. Armstrong, Y. Lin, M. R. Khan, J. Genzer, M. D. Dickey, Adv. Funct. Mater. 2017, 27, 1605630.

2. In Figure 1a, the annotation indicates that the as drawn fibers are filled with liquid metal. Is it already filled or injected after the SEBS fibers are drawn?

3.In the fabrication section, can you specify which gallium-based liquid metal you are using?

4.I think there needs some clarification as to what C_0 or R_0 refers to in the axes of figures. Does it refer to when both axial and torsional strains are zero?

5. The following sentence could be misleading and needs to be rephrased as it is a crucial sentence that describes how the fiber can be used as a dual sensor.

“The capacitive signal exhibits a consistent response to torsional strain within the tensile strain range from 0% to 50%, where the response curves show linear offsets as tensile strain increases (Fig. 4f, top)”.

It could be understood as the capacitive signal being constant with respect to torsion where what you mean to say is that the amount of change with respect to torsion remains the same as axial strain changes.

6. Have you measured the resistance values for the C channels. How does it response to axial and torsional strain? Would it be possible to use just the resistance and capacitance values of the C channel only to acquire decoupled strain measurements?

7.Have you investigated the effect of channel diameter? Smaller diameters would mean less liquid metal can be used. Would smaller diameters make the fiber more or less sensitive to strain? Would it limit the range of torsional strain if the channels were smaller?

Reviewer #2

(Remarks to the Author)
The authors propose a dual-strain optical fiber sensor, and the principle is described with reasonable clarity. The measurement method is rather ingenious, but it lacks novelty. The specific comments are as follows.

1、 During the measurement process, I am concerned whether the central bore of the optical fiber might undergo compressive deformation due to stretching or twisting, as this directly affects the parameters reported by the gallium-based alloy in the core. In Figure 2c, the authors investigate tensile strains from 0% to 200%, yet in subsequent experiments, the applied strain reaches up to 800% (e.g., Figure 4a). Should the authors consider the consistency of variables across these experiments? Meanwhile, when the fiber is subjected to torsion, does the central bore face potential compressive deformation, and why is this issue not addressed in the experimental section?

2、 In the section “Electromechanical and composite motions decoupling performance of dual-strain fibre sensor,” the authors state, “This is attributed to the elongation and cross-sectional contraction of the microchannels during stretching, which makes those with smaller *h* more susceptible to collapse, thereby disrupting liquid metal continuity and leading to premature signal failure.” This statement appears to be inconsistent with the experimental results presented in Figure 2c.

3、 In the subsequent section, Simultaneous monitoring and discrimination of tensile and torsional strain, the authors claim that “the resistance remains largely unaffected by torsional strain, owing to the deformation of the concentric microchannel being negligible under axial torsion.” Is there any supporting evidence or theoretical justification for this statement?

4、 Why are there two channels for measuring capacitance in the dual-strain optical fiber sensor, and how is this number determined?

5、 Why do the experimental values presented in Figures 3C and 3E not mention the chosen value of n?

6、 What does $\Delta R/R_0 < 1\%$ in Figure 3E signify, and how is this value obtained? The manuscript does not mention it in the relevant section. If the authors intend to convey that $\Delta R/R_0$ remains below 1% under all conditions, how should the data presented in Figure 4F be interpreted?

7、 In the description of Figure 4F, a capacitance signal range of 0%–50% is mentioned. Where does this range come from? Shouldn’t the signal magnitude increase with increasing tensile strain? The same question applies to the resistance signal. Moreover, in the top panel of Figure 4F, the “tensile strain” axis lacks a horizontal scale.

8、 In the pre-torsion step of the experiment, does the dual-strain optical fiber sensor undergo automatic unwinding? Could the applied torque in this step interfere with the measurements?

9、 The principles underlying the numerical model presented in Figure 5A are not clearly explained, and the methodological process remains ambiguous. Meanwhile, when the motion is a composite one, even if a certain offset in $\Delta R/R_0$ is observed, can the corresponding tensile values be clearly defined? For instance, as shown in Figures 3C and 3E, $\Delta C/C_0$ follows an approximately linear relationship in Figure 3C and a roughly quadratic relationship in Figure 3E. When these two effects are coupled, the resulting behavior should not be represented as a simple linear offset

10、 The number of heavy-object samples tested in the experimental section is limited, and at a minimum, the relevant parameters of these objects should be reported. How do these parameters influence the test results?

Reviewer #3

(Remarks to the Author)
This manuscript reports a stretchable tri-channel fibre sensor with concentric and double-helical microchannels filled with gallium-based liquid metal, designed to decouple tensile and torsional deformations. The authors demonstrate enhanced stretchability and durability due to the helical chain orientation, and the sensor achieves dual resistive-capacitive sensing with good linearity and bidirectional torsional sensitivity. A digital twin platform is further developed for real-time monitoring and motion decoupling. However, in my opinion, the level of innovation is insufficient for publication in Nature Communications. Research on liquid metal-based fibre sensors is already extensive, and the present study mainly modifies the fibre structure without introducing a fundamentally new sensing mechanism or material concept. While the engineering optimization is carefully executed, the contribution does not substantially advance the state of the art.

1. The manuscript introduces a tri-channel fibre with two helical and one concentric conductive pathways. However, the necessity of incorporating three electrodes is not sufficiently justified. Since two electrodes could already accomplish capacitive sensing, the authors should clearly explain why three conductive channels are required and establish a detailed mathematical model of the double-helical capacitor.

2. In Fig. 4c, the response times are provided, but the corresponding applied stretching or torsional input times are not marked. The authors should indicate the precise timing of mechanical loading to ensure that the reported response times are clearly validated.

3. The maximum torsional angle that the fibre can withstand is not reported. The authors should specify the upper torsional limit and clarify at what angle the fibre undergoes transition from pure torsion to tensile deformation.

4. The mechanical and electrical hysteresis behaviour of the fibre sensor should be systematically characterised, as hysteresis is critical for evaluating reliability and long-term sensing performance.

5. A comparative study is needed on fibres with different helical periods, specifically regarding their maximum stretchable length. In addition, the authors should report the elongation at break before and after liquid metal filling, to clarify the influence of structural design and filler on the mechanical robustness.

Version 1:

Reviewer comments:

Reviewer #1

(Remarks to the Author)
Thank you for addressing the comments and providing the corresponding clarifications. The tri-channel fiber geometry appears to be an effective approach for decoupling strain and torsion, and I was particularly impressed by the close agreement between the theoretical predictions and the experimental results. I also find the approach of treating the change in resistance as a known quantity and incorporating it into the capacitance model to be a practical and well-justified strategy for real-world applications.

Out of curiosity, I had one question regarding the distinction between the equation presented on page 9 of the response and the change-in-resistance equation in Supplementary Note 4. Were these equations derived using different assumptions or methods, and if so, what prevents the equation in the supplementary information from being used in the proposed practical application strategy?

Reviewer #2

(Remarks to the Author)
In the previous response, the author has addressed the reviewer's comments, but some issues still remain to be resolved.

When addressing whether the pre-torsion step could affect the measurement results, the authors' statement that the sensor response is "independent of external forces" is overly absolute, since sensor strain is inherently induced by external loading and the decoupling relies on strain signals. Please clarify whether different loading rates (dynamic impact vs. quasi-static) could alter the response due to material viscoelasticity and liquid-metal inertia. The experiments currently cover only quasi-static and low-frequency oscillations, without validation under dynamic loading.
In addition, how is the pre-torsion level determined? Is the same pre-torsion angle applied across different load masses, or is it adjusted depending on the load?

In responding to the question about the necessity of the three channels, if the authors emphasize that noise/coupling can undermine the stability of a simultaneous solution, they should also provide an error or robustness assessment for the sequential method (even a simple sensitivity analysis or repeatability statistics). Otherwise, the argument is not fully convincing.

In response to the reviewer's questions on the model construction in Figure 5A and the identifiability of ε from $\Delta R/R0$ under combined motion, the authors mainly restate the decoupling procedure without sufficient detail for reproducibility. Please (1) specify how the mathematical/numerical model is built, and (2) provide an error bound for ε estimation and a method to handle linear drift/offset. Otherwise, the current description is difficult to reproduce and verify independently.

In the final experiment using a 50 g weight, the authors state that "tests were conducted with weights of different masses, and the 50 g weight is presented as a representative case." It is recommended that the authors summarize the tested load conditions in a table, or at least provide 2–3 additional representative cases, to support the claim that the results do not rely on a specific load.
The claim that the response is "largely independent of load parameters" may be inaccurate. Load mass/geometry changes inertia, which can modify the coupled tensile–torsional dynamics and thus the strain waveforms in dynamic conditions. Please discuss this dependence and/or provide comparative tests with different masses/shapes to demonstrate the robustness of the decoupling algorithm under dynamic loading.

Reviewer #3

(Remarks to the Author)
This manuscript presents a high-quality and well-executed engineering study with thorough characterization and convincing performance demonstrations. However, the central claims of novelty—particularly concerning rotational thermal stretching and dual-strain decoupling—require stronger mechanistic substantiation to rise to the level of conceptual innovation expected for publication in this venue.

1. The claimed novelty of rotational thermal stretching in enhancing mechanical properties is not convincingly demonstrated at the mechanistic level. Thermal drawing is already known to improve fiber mechanics by inducing polymer chain orientation. To establish true novelty, the authors would need to show that rotation leads to a unique structural or molecular outcome beyond conventional hot drawing (e.g., via direct comparison against non-rotational drawing or quantitative orientation analysis). At present, the manuscript mainly reports structural changes and improved performance, rather than a distinct mechanism attributable to rotation.

2. The tri-channel fiber architecture improves robustness and dual-mode sensing, but appears to reflect architectural optimization of existing LM-fiber strategies. The sensing concept and physical mechanisms do not depart substantially from prior works in multi-modal LM fibers.

3. The decoupling approach is achieved through additional channels and analytical inference, rather than through intrinsic self-decoupling. While functional, this represents a system-level integration strategy rather than a new sensing mechanism.

Version 2:

Reviewer comments:

Reviewer #1

(Remarks to the Author)
Thank you for your thorough and thoughtful responses to my comments. I appreciate the care you have taken to address each point clearly and comprehensively. I have no further comments and believe the manuscript is now suitable for publication.

Reviewer #2

(Remarks to the Author)

Reviewer #3

(Remarks to the Author)
The author has answered my questions. I recommend that this paper be published.

Open Access This Peer Review File is licensed under a Creative Commons Attribution 4.0 International License, which permits use, sharing, adaptation, distribution and reproduction in any medium or format, as long as you give appropriate credit to the original author(s) and the source, provide a link to the Creative Commons license, and indicate if changes were

made.
In cases where reviewers are anonymous, credit should be given to 'Anonymous Referee' and the source.
The images or other third party material in this Peer Review File are included in the article's Creative Commons license, unless indicated otherwise in a credit line to the material. If material is not included in the article's Creative Commons license and your intended use is not permitted by statutory regulation or exceeds the permitted use, you will need to obtain permission directly from the copyright holder.
To view a copy of this license, visit https://creativecommons.org/licenses/by/4.0/

Response Letter

Manuscript ID: NCOMMS-25-55171-T

Title: A highly stretchable tri-channel fibre for composite motions decoupling

Author: Zhangcheng Li, Wen Wang, Kangrui Ji, Can Wang, Pan Xiong, Zeguang Du, Zhi Liang, Yu He, Bijin Xiong, Guangming Tao, and Chong Hou

Formatting Note:

Responses to the reviewers' comments are provided in blue. Corresponding revisions in the manuscript are highlighted in red.

Response to Reviewer #1:

This work presents a method of creating a tri-channel hollow fiber that is injected with liquid metal that can be used as a strain sensor. It uses a combination of resistance and capacitance measurements to determine the axial and torsional strain state. It provides a thorough characterization of its electromechanical properties and demonstrates its use as a sensor. The manuscript is coherently written and is recommended for publication after the following revisions are made.

Response: We would like to express our sincere appreciation to the reviewer for the thoughtful comments and constructive suggestions provided on our manuscript. Your feedback has been very helpful in guiding our revisions and has contributed significantly to improving the quality of the paper. We have carefully considered all the points raised and have incorporated the corresponding changes into the revised version. We believe the revisions have substantially improved the manuscript and hope they meet with your approval.

1. There has been prior work on fabrication and characterization of liquid metal injected SEBS fibers (https://doi.org/10.1002/adfm.201202405) Such fibers have been used to make helical structures for capacitive sensing including torsional measurements. I certainly think it is worth mentioning.

C. B. Cooper, K. Arutselvan, Y. Liu, D. Armstrong, Y. Lin, M. R. Khan, J. Genzer, M. D. Dickey, Adv. Funct. Mater. 2017, 27, 1605630. https://doi.org/10.1002/adfm.201605630

Response: We thank the reviewer for highlighting important prior research regarding the integration of SEBS fibres with liquid metal and their applications in helical capacitive sensing and torsion measurement [*Adv. Funct. Mater.* 2013, 23, 2308; *Adv. Funct. Mater.* 2017, 27, 1605630]. We have also taken note of several pertinent works [*Nat. Commun.*

2021, 12, 1416; *Nat. Electron.* 2025, 8, 1072–1081], which provided valuable inspiration for our research. Relative to these related studies, our contributions in this work are as follows:

1. We introduce rotation of the preform during the thermal drawing process, which induces helical alignment of polymer chains along the fibre axis and dramatically enhances the mechanical properties of the fibre. This is evidenced by the increase in elongation at break from 805% to 1,918% and in tensile strength from 8.68 MPa to 15.91 MPa.
2. We fabricated a dual-strain fibre sensor that exhibits exceptional performance metrics, including: (1) a highly linear response to tensile strain, with $R^2 > 0.999$ over a broad range of 0-800%; (2) a wide bidirectional torsion sensing range of ±2000 rad/m; and (3) excellent durability exceeding 75,000 cycles at 200% tensile strain and 425,000 at ±1,000 rad/m torsional strain.
3. We realize a strategy that is capable of decoupling composite tensile-torsional deformations. This is achieved using a single fibre incorporating distinct microchannel architectures and a dedicated algorithm. This methodology overcomes the limitations of conventional sensors and provides an unprecedented solution for accurate multimodal sensing for complex deformation.

In response, we have incorporated these pertinent references into the revised manuscript, and explicitly cite them in the relevant sections to better contextualize our work and highlight its innovation within the existing literature.

[On page 2] Particularly in the context of the rapid growth of robotics and wearable technologies, monitoring and decoupling composite mechanical motions—such as elongation, bending, and torsion—have emerged as critical capabilities for executing complex tasks in dynamic operational environments. This imposes stringent requirements on the flexibility, durability, and strain-response performance of the devices[14-17].

[14] Zhu, S. et al. Ultrastretchable Fibers with Metallic Conductivity Using a Liquid Metal Alloy Core. *Advanced Functional Materials* **23**, 2308-2314 (2012).

[15] Cooper, C. B. et al. Stretchable Capacitive Sensors of Torsion, Strain, and Touch Using Double Helix Liquid Metal Fibers. *Advanced Functional Materials* **27** (2017).

[16] Chen, M. et al. Self-powered multifunctional sensing based on super-elastic fibers by soluble-core thermal drawing. *Nature Communications* **12** (2021).

[17] Laperrousaz, S. et al. Electronic fibres via the thermal drawing of liquid-metal-embedded elastomers. *Nature Electronics* **8**, 1072-1081 (2025).

2. In Figure 1a, the annotation indicates that the as drawn fibers are filled with liquid metal. Is it already filled or injected after the SEBS fibers are drawn?

Response: We appreciate the reviewer for this comment. The liquid metal is injected into the microchannels within the tri-channel fibre after the thermal drawing process. The relevant methodological details, initially presented on page 6 of the original manuscript, have been further elaborated in the revised version to enhance clarity and emphasis:

[On page 6] Through thermal drawing, the tri-channel fibre exhibiting exceptional flexibility and stretchability can be fabricated in continuous lengths. Subsequently, a highly conductive and fluid gallium-based liquid metal was infused into the microchannels to establish stretchable conductive pathways throughout the fibre.

3. In the fabrication section, can you specify which gallium-based liquid metal you are using?

Response: We thank the reviewer for raising this point. As indicated in the Methods section of our manuscript, the gallium-based liquid metal utilized in our fabrication process is a eutectic Ga-In-Sn alloy, with a composition of 68.5wt% Ga, 21.5wt% In, and 10wt% Sn.

[On page 25] *Assembly of the dual-strain fibre sensor.* Gallium-based liquid metal (68.5wt% Ga, 21.5wt% In, and 10wt% Sn, Sino Santech Materials Technology Co., Ltd.) was injected into the three microchannels of the fabricated fibres using a syringe.

4. I think there needs some clarification as to what C_0 or R_0 refers to in the axes of figures. Does it refer to when both axial and torsional strains are zero?

Response: We appreciate the reviewer's comment regarding the definition of key parameters. The terms C_0 and R_0 correspond to the initial capacitance and resistance of the fibre in its pristine, unstrained state. In the revised manuscript, we have provided clear definitions for C_0 and R_0, along with other key symbols in the figures and their corresponding legends.

[On page 13] C_0 and R_0 denote the initial capacitance and resistance of the fibre in its unstrained state, respectively. ΔC and ΔR represent the absolute changes in capacitance and resistance from their initial values following the application of strains. The corresponding relative changes are defined as $\Delta C/C_0$ and $\Delta R/R_0$.

5. The following sentence could be misleading and needs to be rephrased as it is a crucial sentence that describes how the fiber can be used as a dual sensor.

"The capacitive signal exhibits a consistent response to torsional strain within the tensile strain range from 0% to 50%, where the response curves show linear offsets as tensile strain increases (Fig. 4f, top)".

It could be understood as the capacitive signal being constant with respect to torsion where what you mean to say is that the amount of change with respect to torsion remains the same as axial strain changes.

Response: We gratefully acknowledge the reviewer for this insightful comment. The original wording was ambiguous and susceptible to misinterpretation. Our intended

meaning was not to indicate that the capacitive signal is constant, but rather that the sensor's capacitive response pattern to torsional strain remains consistent and predictable across different levels of applied tensile strain (Fig. R1a, bottom). Furthermore, under fixed torsional strains, the capacitance exhibits a linear response to increasing tensile strain, as demonstrated in Fig. R1b.

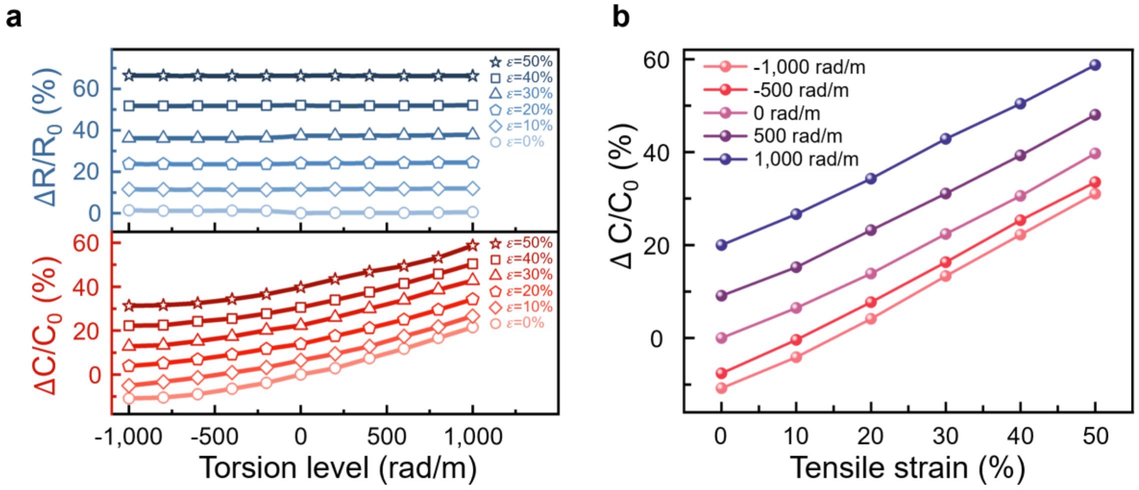

Fig. R1 | Resistive and capacitive response characteristics under composite strain. a, Resistive and capacitive response of the sensor measured under torsional strain at various fixed tensile strains. **b,** Linear capacitive response of the sensor to tensile strain under various fixed torsional strains.

In the revised manuscript, we have incorporated modifications and additions to provide a clearer illustration of the resistive and capacitive response characteristics of the dual-mode fibre sensor under concurrent tensile and torsional strain.

[On page 17] As shown in Fig. 4f, the two sensing modalities demonstrate fundamentally distinct response characteristics under hybrid mechanical deformation. Specifically, the resistive response remains insensitive to torsional strain, regardless of the tensile strain level within the 0 – 50% range (Fig. 4f, top, and Supplementary Fig. 19a). In contrast, the capacitive response pattern to torsional strain remains consistent and predictable across different levels of applied tensile strain (Fig. 4f, bottom). Furthermore, under fixed

torsional strain, the capacitive response exhibits a linear dependence on increasing tensile strain (Supplementary Fig. 19b).

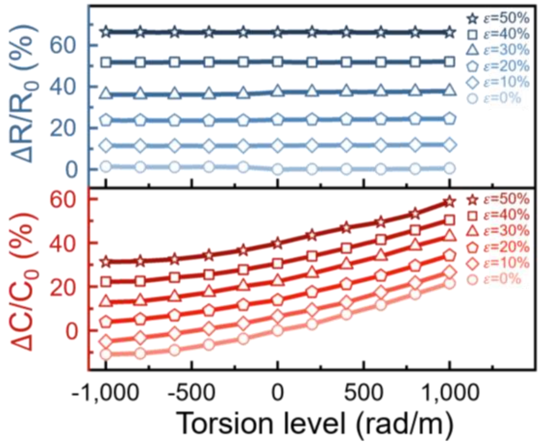

Fig. 4f, Resistive and capacitive response of the sensor measured under torsional strain at various fixed tensile strains.

6. Have you measured the resistance values for the C channels. How does it response to axial and torsional strain? Would it be possible to use just the resistance and capacitance values of the C channel only to acquire decoupled strain measurements?

Response: We appreciate the reviewer for this perceptive comment. The electromechanical response characteristics of the resistance in capacitance channel under tensile and torsional strain are shown in Fig. R2.

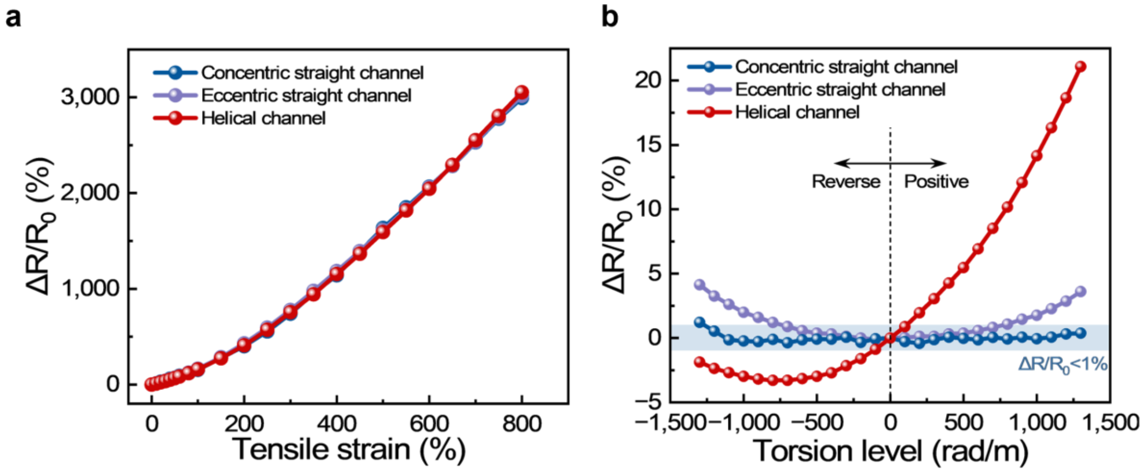

Fig. R2 | Strain-response characteristics of electrical resistance in capacitance channels with different structural configurations. a, Resistance response of concentric straight, eccentric straight, and helical channels under tensile strain. **b,** Resistance response of concentric straight, eccentric straight, and helical channels under torsional strain.

The term "eccentric straight channel" refers to a single electrode of the straight capacitance channel within the fibre (i.e., the helical period $h=\infty$). As shown in Fig. R2a, the resistance responses of the concentric straight, eccentric straight, and helical channels under tensile strain are largely indistinguishable, all following a quadratic dependence on the applied tensile strain.

In contrast, under torsional strain, the three channel architectures exhibit markedly distinct resistance responses, as presented in Fig. R2b. The resistance of the concentric straight channel remains virtually constant under torsional strain, exhibiting a relative change of less than 1%, which confirms its insensitivity to torsion. Under both positive and reverse torsion, the eccentric straight channel progressively deforms into a helical geometry as the torsion level increases. This deformation leads to a concomitant increase in resistance and a symmetric response, thereby precluding discrimination of the torsion direction. For the helical channel, positive torsion increases the resistance, while reverse torsion decreases it, reaching a minimum when the helix is fully unwound into a straight configuration. Consequently, the resistance of the helical channel demonstrates a distinct asymmetric response to torsional strain, similar to the response behaviour of capacitance in double helical channel under torsion.

Theoretically, tensile strain (ε) and torsional strain (γ) can be decoupled from composite deformation by modeling the simultaneous resistance and capacitance responses of the capacitive channel. Theoretical analysis reveals that the relative changes in resistance ($\Delta R/R_0$) and capacitance ($\Delta C/C_0$) can be expressed as functions of ε and γ, respectively:

$$\frac{\Delta R}{R_0}(\varepsilon,\gamma)=\frac{(1+\varepsilon)^3+\left[\frac{\pi d_0}{h_0}+(1+\varepsilon)\frac{\gamma d_0}{2}\right]^2}{(1+\varepsilon)\left[1+\left(\frac{\pi d_0}{h_0}\right)^2\right]}-1$$

$$\frac{\Delta C}{C_0}(\varepsilon,\gamma)=\sqrt{\frac{(1+\varepsilon)^3+\left[\frac{\pi d_0}{h_0}+(1+\varepsilon)\frac{\gamma d_0}{2}\right]^2}{(1+\varepsilon)\left[1+\left(\frac{\pi d_0}{h_0}\right)^2\right]}}\frac{\ln\left(\frac{d_0}{2r_0}+\sqrt{\left(\frac{d_0}{2r_0}\right)^2-1}\right)}{\ln\left\{\frac{d_0}{2r_0}\left\{\frac{(1+\varepsilon)^3+\left[\frac{\pi d_0}{h_0}+(1+\varepsilon)\frac{\gamma d_0}{2}\right]^2}{(1+\varepsilon)^3\left[1+\left(\frac{\pi d_0}{h_0}\right)^2\right]}\right\}^{1/4}+\sqrt{\left(\frac{d_0}{2r_0}\right)^2\left\{\frac{(1+\varepsilon)^3+\left[\frac{\pi d_0}{h_0}+(1+\varepsilon)\frac{\gamma d_0}{2}\right]^2}{(1+\varepsilon)^3\left[1+\left(\frac{\pi d_0}{h_0}\right)^2\right]}\right\}^{1/2}-1}\right\}}-1$$

where d_0 is the initial distance between the centers of the two helical channels, h_0 is the initial helical period, and r_0 is the initial radius of the channel. Although theoretically feasible to determine ε and γ by simultaneously measuring $\Delta R/R_0$ and $\Delta C/C_0$, this approach encounters significant practical challenges. First, the disparity in response characteristics between resistance and capacitance signals to different strain modes can introduce substantial errors during equation solving. Second, simultaneous acquisition of both signals from a single channel may suffer from measurement crosstalk, circuit coupling, and noise interference, thereby compromising signal independence and decoupling accuracy. Consequently, relying solely on the capacitive channel for decoupling remains unreliable in practical applications.

To address these limitations, we implemented a robust strategy leveraging the unique property of the concentric straight channel: its resistance is insensitive to torsional strain and responds exclusively to tensile strain within the operating range, as described by the following relationship:

$$\frac{\Delta R}{R_0}(\varepsilon,\gamma)=(\varepsilon+1)^2-1$$

Under composite strain conditions, this property allows the tensile strain component to be quantified independently and directly from the resistance change. Subsequently, the determined tensile strain is substituted into the capacitive response model as a known variable, enabling the accurate resolution of the corresponding torsional strain component.

This approach not only significantly reduces algorithmic complexity and real-time processing burdens, but also enhances system immunity to interference and overall

robustness in dynamic environments through signal isolation and sequential solving. This strategy thereby ensures stable and reliable real-time dual-strain monitoring, providing an effective solution for decoupling measurements in complex mechanical environments.

7. Have you investigated the effect of channel diameter? Smaller diameters would mean less liquid metal can be used. Would smaller diameters make the fiber more or less sensitive to strain? Would it limit the range of torsional strain if the channels were smaller?

Response: We thank the reviewer for this valuable comment regarding the critical parameter of channel diameter. Through combined theoretical analysis and supplementary experiments, we have systematically investigated the influence of channel diameter ($\boldsymbol{\phi}$) on both the sensitivity and operational range of the sensor. To address this point, we characterized the electromechanical response of a dual-strain fibre sensor with a 5 mm helical period, testing channel diameters of 80, 120, and 160 μm under both tensile and torsional strain. Cross-sectional images of the corresponding fibres are presented in Fig. R3a-c.

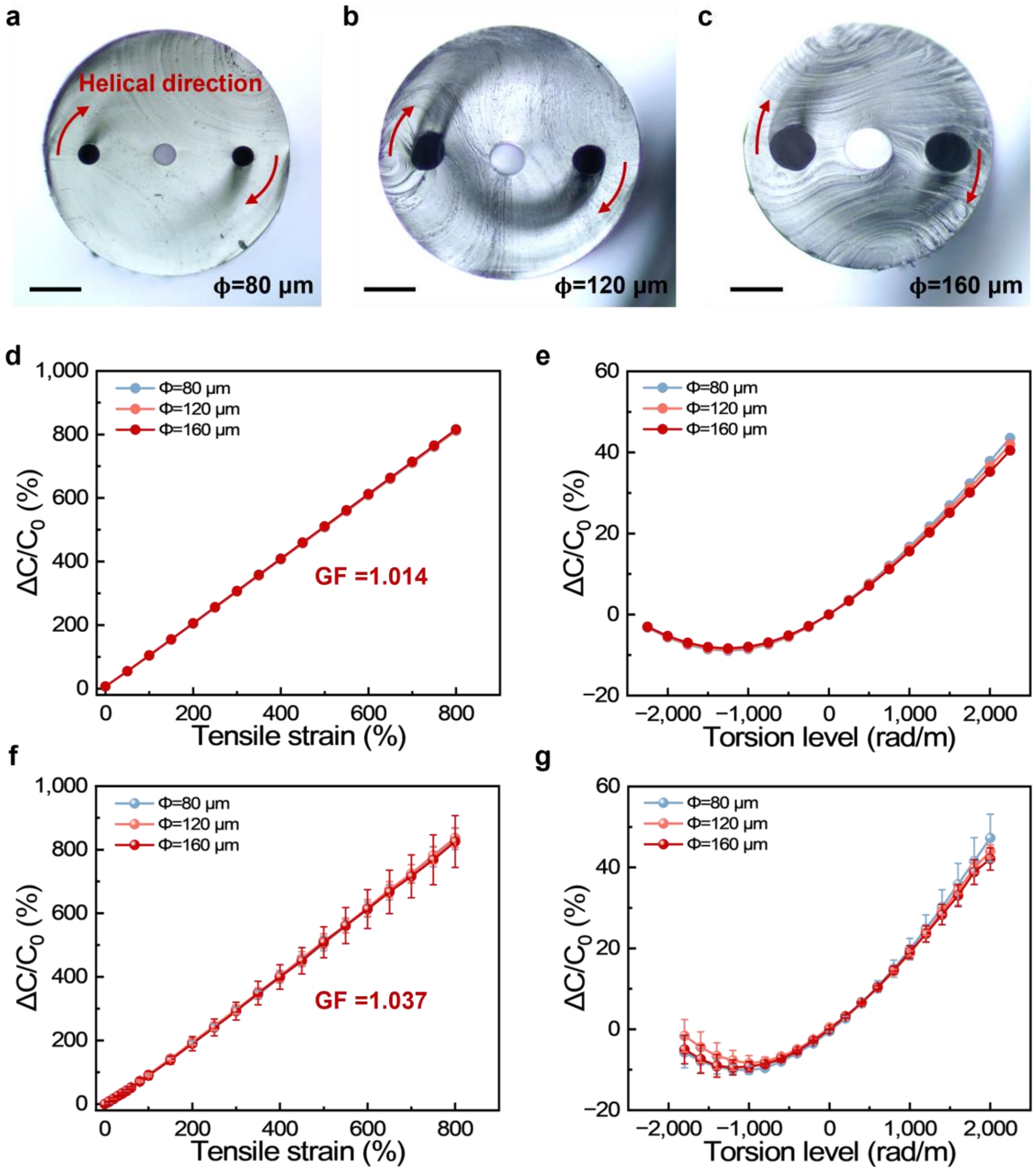

Fig. R3 | Electromechanical response of dual-strain fibre sensors (h = 5 mm) under tension and torsional strain with different microchannel diameter ϕ. a-c, Cross-sectional images of the tri-channel fibre with microchannel diameters of 80 μm (a), 120 μm (b), and 160 μm (c). Scale bar, 200 μm. **d, e,** Theoretically calculated relative changes in capacitance for the dual-strain fibre sensor with different ϕ under tensile strain (d) and torsional strain (e). **f, g,** Experimentally measured relative changes in capacitance for the dual-strain fibre sensor with different ϕ under tensile strain (f) and torsional strain (g).

The relative capacitive change ΔC/C0 under pure tensile strain ε can be expressed as (see Supplementary Note 4 for details):

$$\frac{\Delta C}{C_0}(\varepsilon) = (1+\varepsilon)\sqrt{Q} \times \frac{\ln\left(\mathrm{A}+\sqrt{A^2-1}\right)}{\ln\left(\mathrm{A}Q^{1/4}+\sqrt{A^2Q^{1/2}-1}\right)} - 1$$

where $A = \frac{d_0}{\boldsymbol{\phi}_0}$ is a parameter related to the microchannel diameter, $B = \frac{\pi d_0}{h_0}$ is a parameter related to the helical period, and $Q = \frac{(1+\varepsilon)^3+B^2}{(1+\varepsilon)^3(1+B^2)}$.

Similarly, the relative capacitive change ΔC/C0 under pure torsion level γ is (see Supplementary Note 5 for details):

$$\frac{\Delta C}{C_0}(\gamma) = \sqrt{P} \times \frac{\ln\left(\mathrm{A}+\sqrt{A^2-1}\right)}{\ln\left(\mathrm{A}P^{\frac{1}{4}}+\sqrt{A^2P^{\frac{1}{2}}-1}\right)} - 1$$

where $T = \frac{\gamma d_0}{2}$ is a parameter related to the torsion level, and $P = \frac{1+\left(\frac{\pi d_0}{h_0}+\frac{\gamma d_0}{2}\right)^2}{1+\left(\frac{\pi d_0}{h_0}\right)^2} = \frac{1+(B+T)^2}{1+B^2}$.

Based on the above equations, we calculated the theoretical capacitance response of fibre sensors with different $\boldsymbol{\phi}_0$ under both tensile and torsional strains, as shown in Fig. R3d and e. The results reveal that the electromechanical response characteristics are largely consistent across sensors with different $\boldsymbol{\phi}_0$ under both strains. This consistency arises primarily because the engineered helical period h_0 is significantly larger than the channel spacing d_0, leading to $B \ll 1$. Consequently, the influence of parameter A (and thus the channel diameter $\boldsymbol{\phi}_0$) on the response is significantly attenuated. As a result, the channel diameter has a negligible impact on the overall sensitivity of the sensor. Specifically, the sensitivity to tensile strain remains approximately constant at 1.01, whereas under high positive torsional strain, sensors with smaller channel diameters exhibit a marginally enhanced sensitivity. Notably, the experimental measurements demonstrate excellent agreement with the theoretical calculations, as evidenced in Fig. R3f and g.

In summary, the channel diameter has a negligible influence on the response sensitivity of the fibre sensor. While employing a smaller channel diameter effectively reduces the volume of liquid metal required, it does not compromise the strain measurement range. This understanding provides a valuable guideline for optimising sensor design in our future work.

Response to Reviewer #2:

The authors propose a dual-strain optical fiber sensor, and the principle is described with reasonable clarity. The measurement method is rather ingenious, but it lacks novelty. The specific comments are as follows.

Response: We are deeply grateful for your positive appraisal of our work and the highly constructive suggestions. These insightful recommendations are invaluable for improving the manuscript and guiding our future research endeavors. We wish to clarify that the dual-strain fibre sensor presented in this work operates based on the decoupling of composite strain using electrical signals within the tri-channel stretchable fibre, and it does not involve optical sensing mechanisms. Therefore, the term "dual-strain optical fibre sensor" does not accurately describe our device.

To more emphatically underscore the uniqueness and novelty of our work, the manuscript has been revised to provide a more emphatic elaboration of its key contributions. The novelty of our work is primarily demonstrated through three key aspects:

First, by combining theoretical analysis with experimental characterisation, we have validated for the first time that introducing a rotation process during fibre drawing process significantly enhances the mechanical properties of fibre. We find that rotating the preform during thermal drawing modulates the polymer chain orientation, inducing a helical alignment along the fibre axis, which yields increase from 805% to 1,918% in elongation at break and increase from 8.68 MPa to 15.91 MPa in tensile strength, thereby substantially bolstering the mechanical robustness of both the fibres and the devices. This approach establishes a novel paradigm for advancing the performance and durability of stretchable fibre electronics.

Second, we engineered a high-performance dual-strain fibre sensor that exhibits exceptional performance metrics, including: (i) highly linear response to tensile strain, with $R^2 > 0.999$ over a broad range of 0-800%; (ii) wide bidirectional torsion sensing range of

±2000 rad/m; and (iii) superior durability exceeding 75,000 cycles at 200% tensile strain and 425,000 at ±1,000 rad/m torsional strain. Compared with other state-of-the-art multimodal strain sensors, our dual-strain fibre sensor demonstrates outstanding performance across critical metrics.

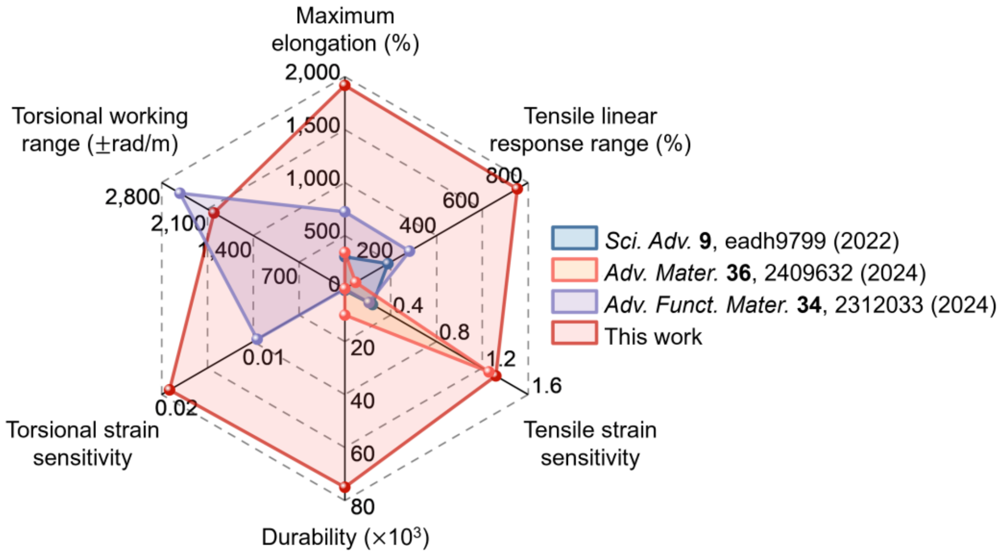

Fig. R4 | Comparison of the performance of the dual-strain fibre sensor with those state-of-the-art strain sensors in previous reports.

Third, we demonstrated a pioneering approach for the accurate, real-time decoupling and monitoring of composite tensile-torsional deformations using our dual-strain fibre sensor. This is achieved through a tri-channel fibre that integrates concentric straight and double-helical microchannel architectures. A dedicated algorithm leverages the differential electrical responses of these distinct microchannels under tensile and torsional loading to achieve real-time decoupling and concurrent measurement of both strain components. This strategy effectively overcomes a key limitation of conventional strain sensors, which typically struggle to decouple multiple strain components under complex loading conditions. More importantly, it provides an unprecedented paradigm for deformation sensing in future fibre electronics and wearable devices destined for authentic, complex

environments. This advancement significantly elevates the capabilities of fibre sensors in terms of multifunctional integration and robust signal decoupling.

All the points raised have been carefully addressed in the revised manuscript. We hope we clarify our work novelty and thank the reviewer again for the time and insightful comments, which have substantially strengthened the manuscript.

1. During the measurement process, I am concerned whether the central bore of the optical fiber might undergo compressive deformation due to stretching or twisting, as this directly affects the parameters reported by the gallium-based alloy in the core. In Figure 2c, the authors investigate tensile strains from 0% to 200%, yet in subsequent experiments, the applied strain reaches up to 800% (e.g., Figure 4a). Should the authors consider the consistency of variables across these experiments? Meanwhile, when the fiber is subjected to torsion, does the central bore face potential compressive deformation, and why is this issue not addressed in the experimental section?

Response: We gratefully acknowledge the reviewer for these comments. We address the raised points as follows:

(1) Mechanical integrity of the liquid metal microchannels under strain

The liquid metal exhibits high fluidity and excellent electrical conductivity (viscosity ≈ 2 mPa·s, $\sigma \approx 3.4\times10^6$ S/m), and is virtually incompressible when confined within the microchannel. Consequently, during tensile or torsional deformation of the fibre, the macroscopic strain is predominantly accommodated by the elastic polymer matrix, whereas the volume of the encapsulated liquid metal microchannel remains constant. Therefore, even under large applied strains, the liquid metal maintains its electrical connectivity, thereby preserving the continuity and geometric integrity of the conductive pathway, as evidenced in Fig. 4d and e. To experimentally validate this mechanism, we directly visualised the liquid metal pathway under torsional strain using X-ray microscopy (XRM).

The results, presented in Fig. R5, confirm that the structural integrity of the pathways is maintained. This property ensures the accuracy and consistency of the fibre sensor's signal output under applied tensile and torsional strains.

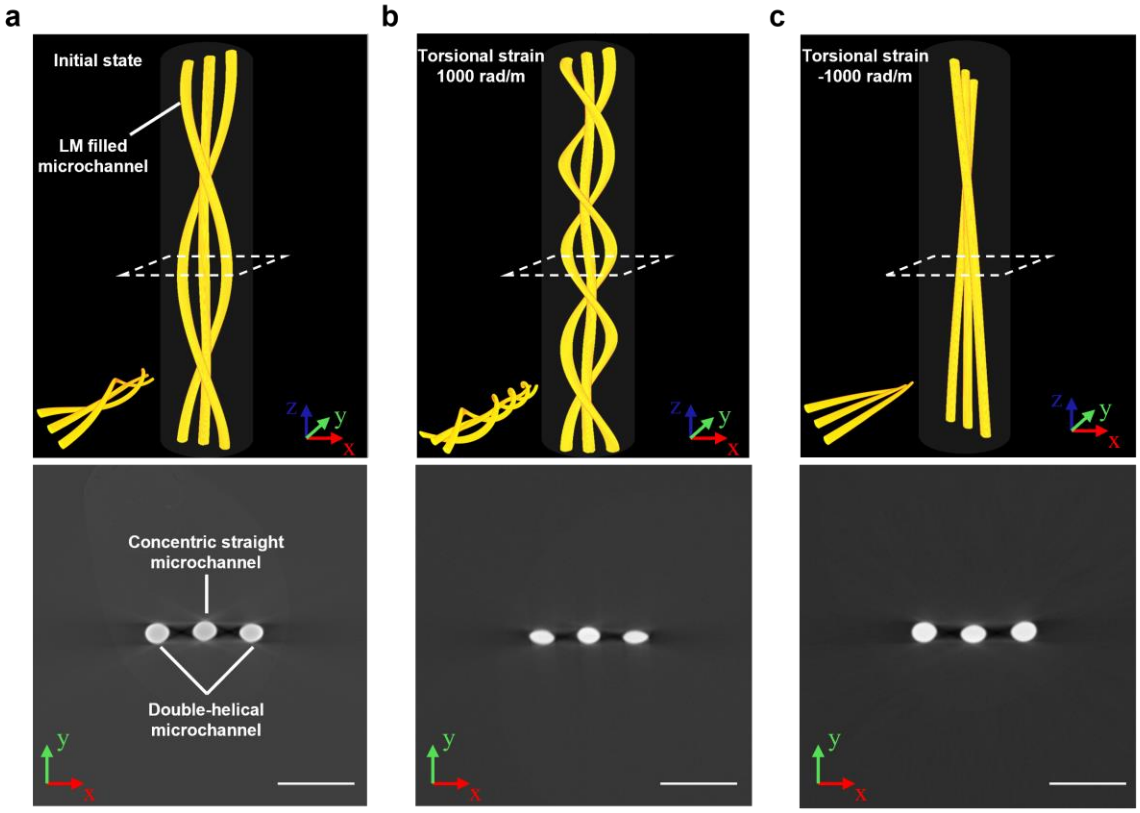

Fig. R5 | 3D XRM reconstructions and cross-sectional CT slices of the tri-channel fibre under different torsional strains. Scale bar, 500 µm. a, 3D structural configuration and cross-sectional CT image of the fibre in its initial state. **b,** Structural configuration and cross-sectional CT image under a positive torsional strain of 1000 rad/m. **c,** Structural configuration and cross-sectional CT image under a reverse torsional strain of -1000 rad/m.

(2) Rationale for the selected strain ranges in different experiments.

We thank the reviewer for this meticulous observation. Fig. 2c presents the SAXS patterns of stretchable fibres with distinct polymer chain orientations, acquired under tensile strains ranging from 0% to 200%. Analysis of these patterns enables the investigation of the microstructural evolution within the fibres during stretching, thereby

facilitating the interpretation of differences in their macroscopic mechanical behaviour.

Specifically, in fibres with helically aligned polymer chains along the axial direction, the front and rear sides exhibit a symmetric orientation distribution during SAXS testing (as shown in Fig. R6a). As a result, the scattering patterns originating from the front and rear sides are symmetric about the fibre axis. The superposition of these symmetric signals yields an elliptical scattering pattern (Fig. R6b), with its long axis perpendicular to the fibre axis. To unambiguously resolve the polymer chain orientation, the fibres were longitudinally sectioned into semi-cylindrical specimens prior to SAXS characterisation (as shown in Fig. R6c). This approach enables the direct and accurate determination of the orientation angle of the polymer chains relative to the fibre axis, as evidenced in Fig. R6d (corresponding to Fig. 2c(ii) at $\varepsilon = 0\%$ in the manuscript). It must be noted that this sample preparation process significantly compromises the structural integrity of the fibres, thereby severely affecting the mechanical properties of the sample.

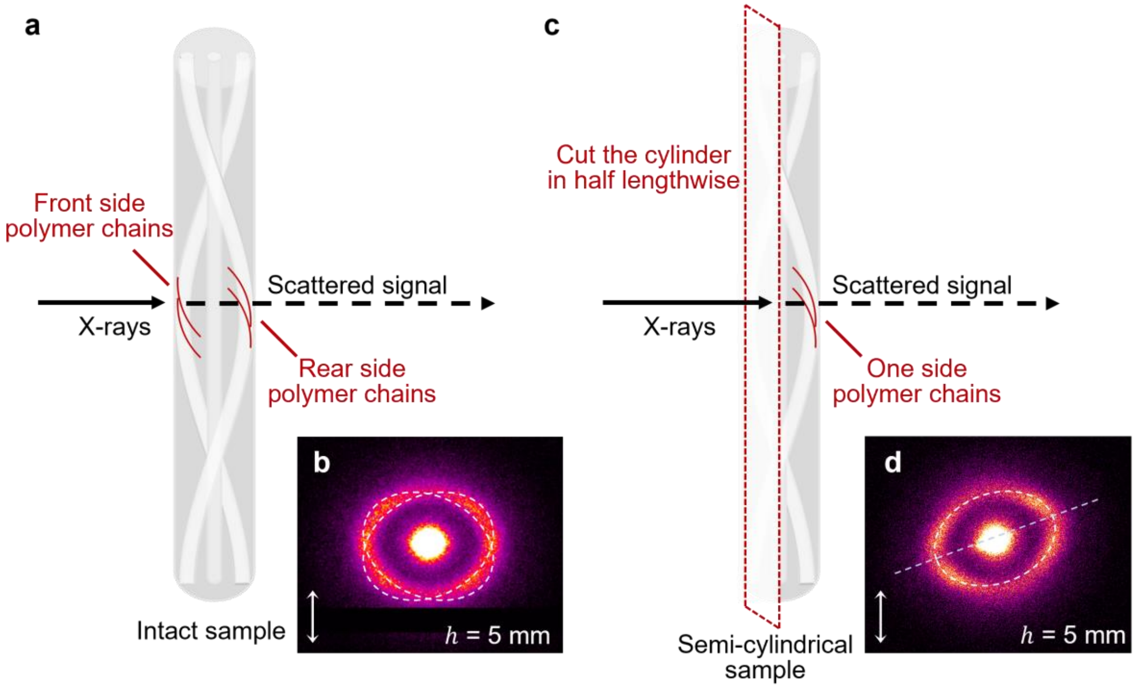

Fig. R6 | Schematic diagrams and SAXS results of samples with different morphologies. a, SAXS measurements of the intact sample reveal a symmetric opposition in polymer chain

orientation between the front and rear sides. **b,** The resulting SAXS pattern arises from the superposition of scattering signals originating from polymer chains on both the front and rear sides, with the arrow denoting the fibre axis direction. **c,** For the semi-cylindrical sample, SAXS interrogation is confined to polymer chains on one side. **d,** The SAXS pattern of the semi-cylindrical sample reflects only the orientation information of the polymer chains on one side.

Consequently, the mechanical performance of the semi-cylindrical samples is substantially inferior to that of the intact fibre. Since the 0-200% strain range is sufficient to effectively analyse the evolution of polymer chain orientation under stretching, this interval was selected for the SAXS characterisation. In contrast, to evaluate the linearity of the sensor response under large strains, the tests described in the section "Electromechanical and composite motions decoupling performance of dual-strain fibre sensor" were performed on the intact fibre structure up to 800% strain (Fig. 4a), which reflects the sensor's practical working range.

The differing strain ranges employed in these two experiments are thus dictated by their distinct objectives: the former aims to elucidate microstructural evolution, while the latter focuses on macroscopic sensing performance. The experimental conditions for each were specifically designed to meet these respective goals, thereby ensuring the validity and consistency of the conclusions. We have accordingly revised and supplemented the manuscript to provide a clearer exposition of the formation mechanism of the SAXS pattern for the fibre featuring helical microchannels.

[On page 8] For intact fibres featuring helical microchannels, the helically aligned polymer chains yield a symmetric, elliptical SAXS pattern, which arises from the superposition of scattering signals. To unambiguously resolve the polymer chain orientation relative to the fibre axis, the fibres were longitudinally sectioned into semi-cylindrical specimens prior to SAXS characterisation (see Supplementary Note 2 and Supplementary Fig. 5 for details).

2. In the section "Electromechanical and composite motions decoupling performance of dual-strain fibre sensor," the authors state, "This is attributed to the elongation and cross-sectional contraction of the microchannels during stretching, which makes those with smaller *h* more susceptible to collapse, thereby disrupting liquid metal continuity and leading to premature signal failure." This statement appears to be inconsistent with the experimental results presented in Figure 2c.

Response: We are grateful to the reviewer for this comment. Comparative analysis of the SAXS patterns in Fig. 2c reveals that under equivalent tensile strain, the scattering ellipse corresponding to the fibre with straight microchannels is flatter than that of its counterpart with helical microchannels. This discrepancy originates from their distinct microscopic orientation architectures. Specifically, in the fibre with helical microchannels, the polymer chains are not fully aligned with the fibre axis but rather extend helically along the axis at a tilt angle δ (Fig. 2a and 2b). Consequently, this three-dimensional helical architecture dictates that upon tensile deformation, both the microdomains and the constituent polymer chains undergo not only axial elongation but also a concurrent process of reorientation and straightening, progressively aligning with the fibre axis (Fig. 2d). Therefore, compared to the fibre with straight microchannels, where polymer chain orientation is parallel to the fibre axis, the fibre with helical microchannels exhibits lower orientation efficiency during stretching. This is evidenced by the comparatively plumper shape of its corresponding SAXS ellipse. This difference in orientation architecture directly affects their macroscopic mechanical properties: fibres with straight microchannels exhibits a higher axial modulus yet inferior toughness, whereas those with helical microchannels achieve superior fracture elongation and enhanced toughness.

As elucidated in the section "Electromechanical and composite motions decoupling performance of dual-strain fibre sensor," fibres with a smaller helical period (h) demonstrate a pronounced propensity for internal microchannel closure under tensile strain.

This behaviour is primarily attributed to a radial contraction of the internal microchannels, which accommodates the macroscopic axial deformation of the fibre (Fig. R7a and Supplementary Note 4). Such strain-induced radial contraction becomes increasingly pronounced with decreasing h.

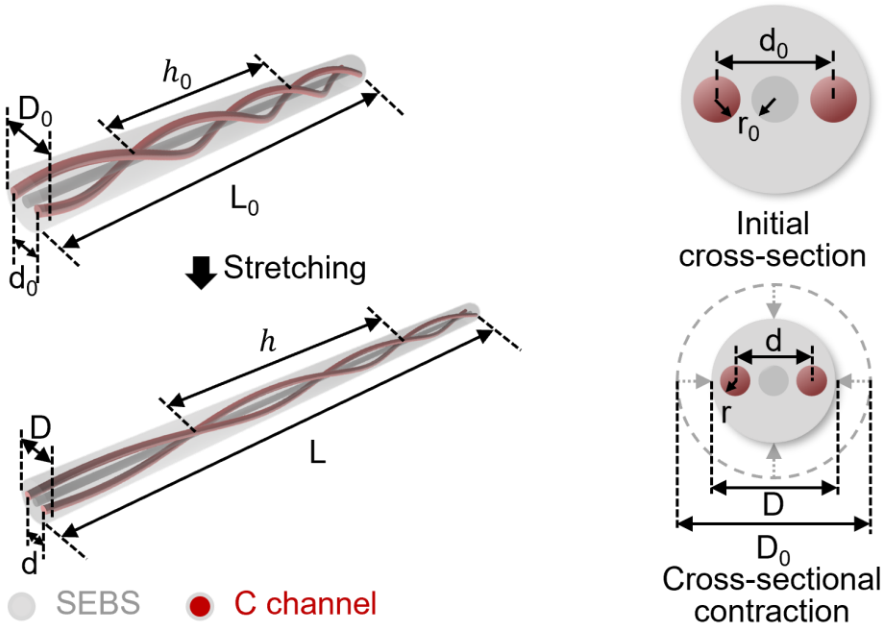

Fig. R7 | Schematic illustrating geometric transformations of the fibre and its internal microchannels under applied tensile strain.

As depicted in Fig. 4a, the capacitive signal of the fibre sensor with h= 2.5 mm vanishes once the applied tensile strain exceeds 220%, indicating the collapse and eventual closure of its internal microchannels. It is noteworthy that the fibre itself remains structurally intact at this strain level, given that its fracture elongation exceeds 1220% (Supplementary Fig. 9a). We primarily ascribe this phenomenon to the inherent geometric instability of the internal microchannels at the macroscopic scale. This mechanism is distinct from the influence of microscopic polymer chain orientation on the bulk mechanical properties of the fibres, as elucidated by SAXS analysis. The relevant sections of the manuscript have been revised and updated accordingly.

[On page 16] For h = 2.5 mm, the electrical capacitive signal fails once the tensile strain surpasses 220%, even though the fibre remains physically intact. This is attributed to the elongation and cross-sectional contraction of the microchannels during stretching, which makes those with smaller h more susceptible to collapse, thereby disrupting liquid metal continuity and leading to premature signal failure.

3. In the subsequent section, Simultaneous monitoring and discrimination of tensile and torsional strain, the authors claim that "the resistance remains largely unaffected by torsional strain, owing to the deformation of the concentric microchannel being negligible under axial torsion." Is there any supporting evidence or theoretical justification for this statement?

Response: We thank the reviewer for raising this point. In this work, a straight concentric microchannel was fabricated within the stretchable fibre to establish a coaxial conductive pathway utilizing liquid metal. From the perspective of elastic mechanics theory, applying pure torsion to a homogeneous, isotropic, linear-elastic cylinder does not induce axial strain. Torsional strain is primarily manifested as relative rotation between cross-sections, leaving the axial length of the fibre unchanged [Timoshenko, S. P., & Goodier, J. N. (1970). *Theory of elasticity* (3rd ed.)]. Furthermore, the shear deformation generated within a certain torsional range constitutes a volume-preserving deformation process. For channels with concentric circular cross-sections, such deformation does not induce significant radial displacement or cross-sectional contraction. Additionally, the confined liquid metal core provides mechanical support to the channel walls, thereby further enhancing the structural stability of the microchannel under torsional strain. Therefore, from a theoretical perspective, deformation of the concentric microchannels under axial torsion within a certain range is negligible.

In the section "Simultaneous monitoring and discrimination of tensile and torsional strain", the resistance response of the internal concentric channels to applied torsional strain was characterized, as presented in Fig 3e. The initial resistance of the fibre in its unstrained state is denoted as R_0. Over an ultra-wide torsional strain range from –1,000 to 1,000 rad/m, the relative resistance change ($\Delta R/R_0$) remained consistently below 1%. This indicates that the electrical resistance of the concentric straight microchannels remained essentially constant, thereby providing indirect confirmation that their deformation under axial torsion is negligible.

To obtain more direct verification, X-ray microscopy (XRM) was performed on the fibre sensor under torsional strain, with the results shown in Fig. R8. Analysis of the cross-sectional slices from the fibre sensor reveals that the concentric channels exhibit no evident irregular deformation, collapse, or closure. This provides direct evidence confirming that the deformation of the concentric microchannels under axial torsion is indeed negligible.

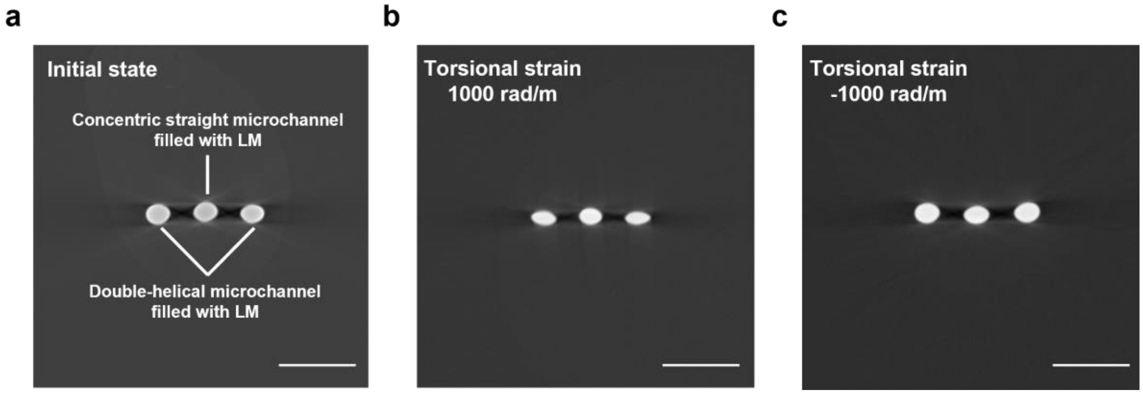

Fig. R8 | Cross-sectional CT slices of the tri-channel fibre under different torsional strains. a, Cross-sectional CT image of the fibre in its initial state. **b,** Cross-sectional CT image under a positive torsional strain of 1000 rad/m. **c,** Cross-sectional CT image under a reverse torsional strain of -1000 rad/m. Scale bar, 500 μm.

4. Why are there two channels for measuring capacitance in the dual-strain optical fiber sensor, and how is this number determined?

Response: We thank the reviewer for this comment. Capacitance is defined as the amount of charge stored per unit voltage between two conductors. As a fundamentally bipolar device, the electrical characteristics of a capacitor are determined exclusively by the configuration and interaction between its two conductive electrodes. Consequently, to transduce tensile strain into a measurable capacitive response, at least two discrete conductive microchannels must be embedded within the fibre. These two channels must be connected simultaneously to form a complete measurement circuit, thereby enabling the detection of capacitance variations in response to applied strain.

In this work, to enable simultaneous decoupling and monitoring of tensile and torsional strains in a single fibre, we designed and fabricated a stretchable fibre incorporating two distinct types of internal microchannels: a concentric straight microchannel and a double-helical microchannels. The concentric straight microchannel functions as a resistive sensor, whereas the double-helical microchannels act as a capacitive sensor (Fig. 3a). Under tensile strain, the resistance signal exhibits a quadratic dependence on strain, in contrast to the linear dependence exhibited by the capacitance signal (Fig. 3c). Under torsional strain, the geometric configuration of the concentric straight channel remains largely unaltered, rendering its resistance signal insensitive to torsion. In contrast, the helical period and length of the double-helical microchannel vary with the direction of torsion, resulting in an asymmetric capacitive output response (Fig. 3e). Leveraging these distinct response characteristics, we simultaneously acquire and analyse the resistance and capacitance signals, thereby achieving decoupling and real-time monitoring of the fibre under combined tensile and torsional strain.

5. Why do the experimental values presented in Figures 3C and 3E not mention the chosen value of n?

Response: We thank the reviewer for raising this comment. In the original manuscript, the symbol "n" was not employed to designate any specific parameter. We surmise that the reviewer may be referring to the helical period, denoted as "*h*", of the helical microchannel embedded within the fibre sensor. The core purpose of Fig. 3 is to clarify the operating principle of the dual-strain fibre sensor for simultaneous decoupling and monitoring under composite strain, for which we utilized a sensor with *h*=5 mm as a representative example. To preclude any ambiguity, we have explicitly annotated this parameter value in the revised caption of Fig. 3.

[On page 13] These structural variations enable the tri-channel fibre to integrate with liquid metal, thereby constructing a dual-strain fibre sensor capable of simultaneously monitoring and discrimination tensile and torsional strains, ultimately enabling the decoupling of composite motions. For the subsequent analysis, a dual-strain fibre sensor with *h*=5 mm is utilized as a representative example.

[Caption of Fig.3] Fig. 3 | Working mechanism of dual-strain fibre sensor for simultaneous monitoring and discrimination of tensile and torsional strains. a, Schematic diagram of the electrical signal outputs from the fibre sensor. The concentric microchannel outputs resistive signals, while the double-helical microchannels output capacitive signals. **b, c,** Schematic diagrams and corresponding response curves showing the variation in resistance and capacitance of the fibre sensor (*h*=5 mm) under tensile strain. **d, e,** Schematic diagrams and corresponding response curves showing the variation in resistance and capacitance of the fibre sensor (*h*=5 mm) under torsional strain.

6. What does $\Delta R/R_0 < 1\%$ in Figure 3E signify, and how is this value obtained? The manuscript does not mention it in the relevant section. If the authors intend to convey that $\Delta R/R_0$ remains below 1% under all conditions, how should the data presented in Figure 4F be interpreted?

Response: We are grateful to the reviewer for this comment. As shown in Fig. R9a (Fig. 3e in the manuscript), R_0 denotes the initial resistance of the concentric straight channel within the fibre in its unstrained state. The condition $\Delta R/R_0 < 1\%$ indicates that under pure torsional strain, the relative resistance change of this concentric straight channel remains below 1% even across a wide torsional strain range from −1,000 to 1,000 rad/m. This characteristic provides a vital foundation for dual-strain decoupling under composite loading: since the resistive channel is insensitive to torsional strain, its change in resistance when both tensile and torsional strains are present can be attributed solely to the tensile component. This allows it to serve as an independent reference for measuring the tensile component during the decoupling process, which significantly simplifies the decoupling model and enhances computational accuracy. This result is derived from statistical analysis of actual measurement data, reflecting the superior signal selectivity achieved through the channel structure design.

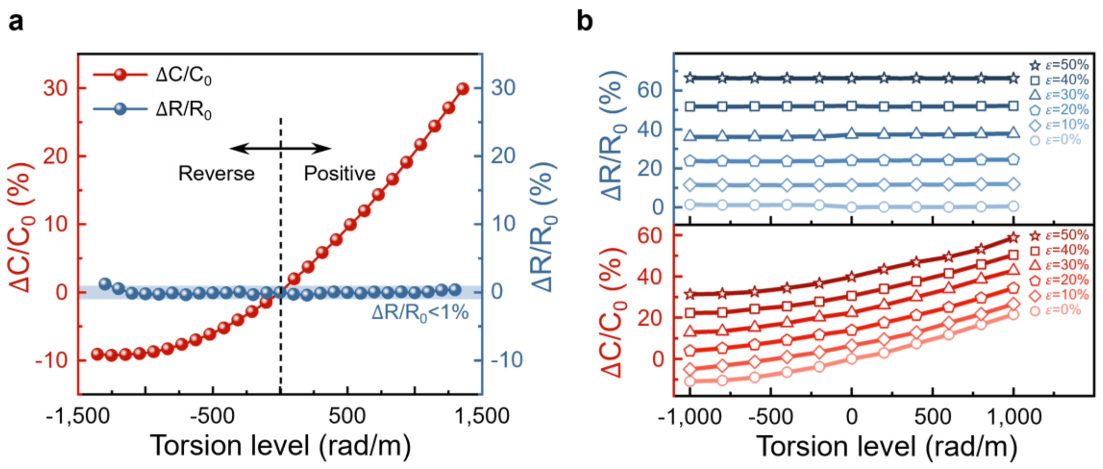

Fig. R9 | Resistance and capacitance responses of the fibre sensor under pure torsion and composed tension–torsion strain. a, Response curves showing the variation in resistance and capacitance of the fibre sensor (h=5 mm) under torsional strain. **b,** Resistive and capacitive response of the fibre sensor (h=5 mm) measured under torsional strain at various fixed tensile strains.

The upper panel of Fig. R9b (revised Fig. 4f in the manuscript) is not intended to demonstrate that $\Delta R/R_0$ remains below 1% under all strain conditions. Rather, it illustrates that under compose tensile and torsional strain, the resistance channel responds exclusively to the tensile component, maintaining its insensitivity to torsion. Each horizontal line corresponds to a fixed tensile strain (ranging from 0 to 50%), under which the relative resistance change remains constant regardless of variations in torsional strain. Numerical analysis of the experimentally acquired data yields the response relationship of the two electrical signals under combined tensile and torsional strains, as presented in revised Fig. 5a. Consequently, under coupled deformation, the tensile strain can be directly quantified from $\Delta R/R_0$ (Fig. 5a, left). Subsequently, by combining the quantified tensile strain with the relative capacitance change in the double-helical channel, the torsional strain can be decoupled and determined (Fig. 5a, right). Thus, this dual-strain fibre sensor enables the simultaneous decoupling and measurement of both tensile and torsional strains. The manuscript and associated figures have been revised accordingly to enhance the clarity and logical flow of the description.

[Revised Fig. 5a]

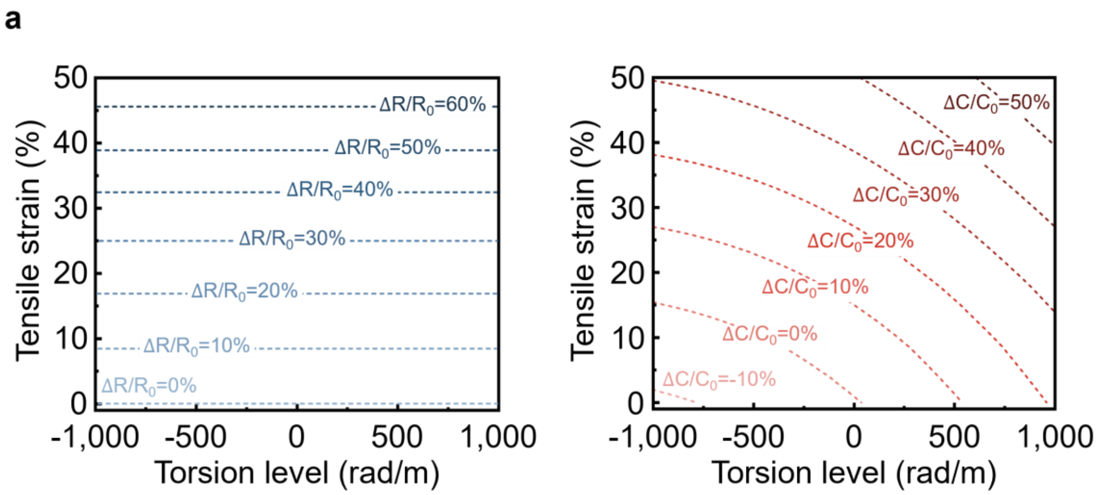

Fig. 5a, Numerical simulation of the dual electrical signals response under coupled tensile and torsional strain.

7. In the description of Figure 4F, a capacitance signal range of 0%–50% is mentioned. Where does this range come from? Shouldn't the signal magnitude increase with increasing tensile strain? The same question applies to the resistance signal. Moreover, in the top panel of Figure 4F, the "tensile strain" axis lacks a horizontal scale.

Response: We thank the reviewer for this comment. In the original version of Fig. 4f (Fig. R10a), the arrow labeled "0%–50%" specifically denotes the applied tensile strain interval and does not represent the relative change rate of the capacitance signal. To ensure sensor integrity during testing while capturing a representative performance range, the tensile strain was varied from 0% to 50% and the torsional strain from –1000 to 1000 rad/m for the dual-strain calibration. Furthermore, the horizontal axes in both panels of Fig. 4f correspond to the torsion level and share identical scale ranges. To maintain visual clarity and conciseness, axis scale markings are provided solely in the bottom panel. The manuscript and the associated figures have been revised to enhance the clarity and logical flow of the relevant descriptions.

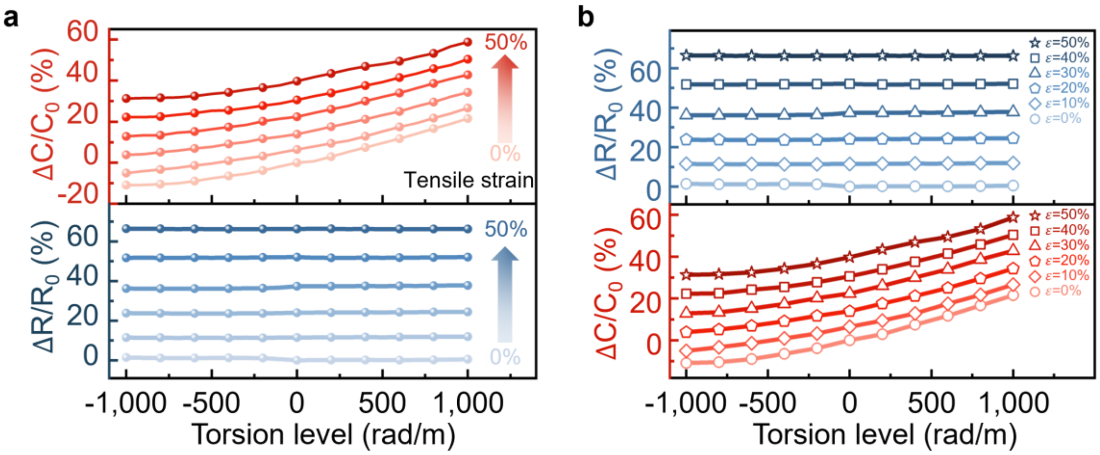

Fig. R10 | Revision of Fig. 4f from the original manuscript. a, Original version. **b,** Modified version.

Additionally, as clarified in our previous response, each curve in Fig. 4f corresponds to a fixed tensile strain, under which the response characteristics of both resistance and

capacitance as functions of torsional strain are depicted. Furthermore, under a constant torsional strain, the relative rates of change for both the resistance and capacitance signals increase progressively with the higher applied tensile strain.

8. In the pre-torsion step of the experiment, does the dual-strain optical fiber sensor undergo automatic unwinding? Could the applied torque in this step interfere with the measurements?

Response: We thank the reviewer for this comment. In all experiments—including both calibration and dual-strain decoupling measurements—the fibre sensor was mounted with one end fixed and the opposite end free to twist (Fig. R11a). This approach inherently prevents the automatic unwinding effect. Therefore, during the pre-twisting phase, the dual-strain fibre sensor does not undergo automatic unwinding.

It is important to note that the dual-strain fibre sensor decouples and measures the inherent deformation state of the fibre itself, independent of the magnitude of external forces. Regardless of whether the deformation is induced by external loading or the fibre's own dynamic recovery following the removal of the external force, tensile and torsional strains can be decoupled and monitored in real time solely by analyzing the internal electrical signals. Before performing the composite strain decoupling experiments, we performed a systematic calibration of the dual-strain fibre sensor. Throughout the pre-twisting phase, the applied torque and the corresponding strain state had been decoupled and monitored in real time using our integrated resistive-capacitive dual-mode sensing mechanism (Fig. R11c). Following the pre-twisting, the suspended weight was released. The sensor then continued to decouple and monitor in real time the subsequently superimposed tensile and torsional strains. A complete visual record of this process is provided in Supplementary Movie 1.

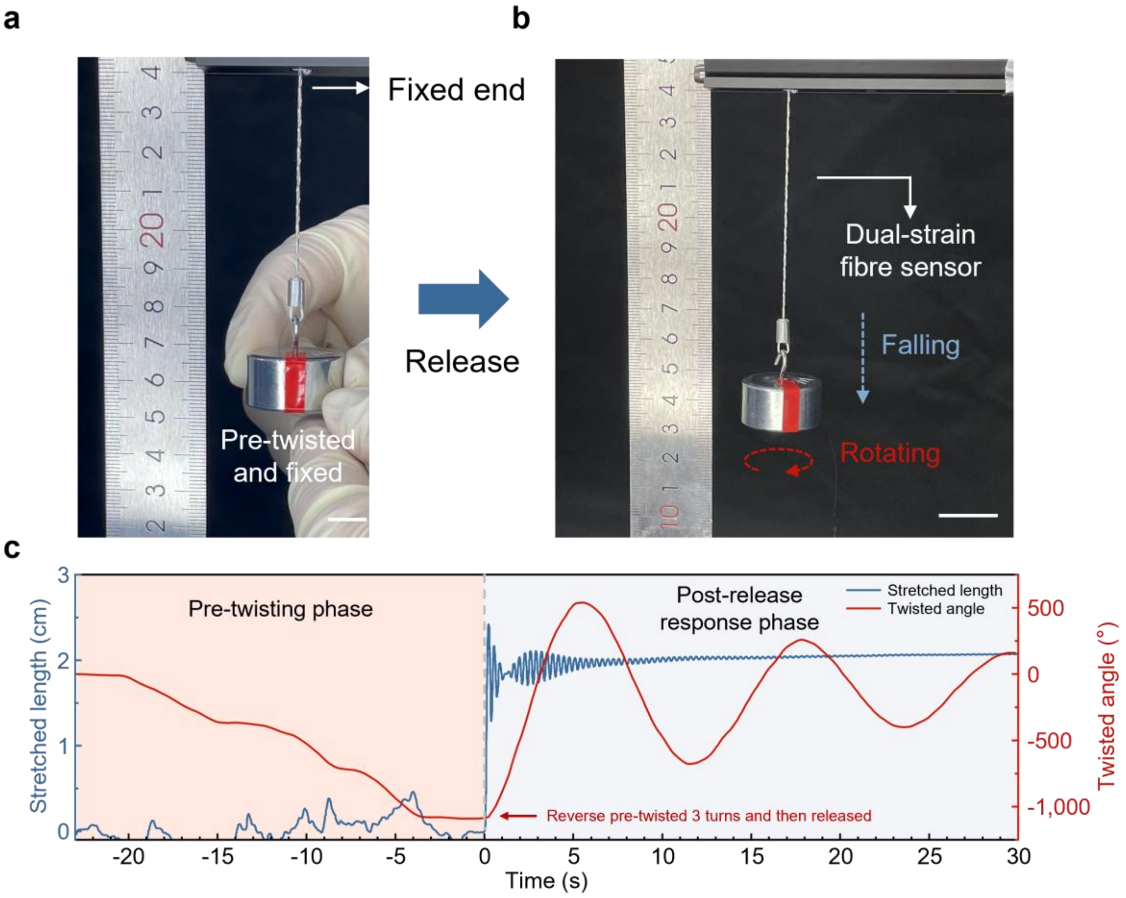

Fig. R11 | Detailed experimental procedure for composite strain measurement. a, The fibre sensor is fixed at one end, with the opposite end left free to undergo torsion. The illustration depicts the state following pre-twisting and prior to weight release. **b,** Upon release of the weight, the fibre sensor enables real-time decoupling and monitoring of the dynamically superimposed tensile and torsional strain fields. **c,** Complete response data of the dual-strain fibre sensor throughout the composite strain decoupling experiment, encompassing the initial pre-twisting phase through to the dynamic response period following weight release.

In the manuscript, the time point immediately following the pre-twisting stage was selected as the analysis baseline to clearly showcase the sensor's capability to decouple and sense composite strains. This choice was made because the manually applied pre-twist is temporally non-uniform, whereas the post-release motion constitutes a highly controllable and repeatable dynamic loading regime. This regime more effectively

highlights the core performance metrics of the sensor. We have updated the relevant figures and text in the revised version.

[Revised Fig. 5e]

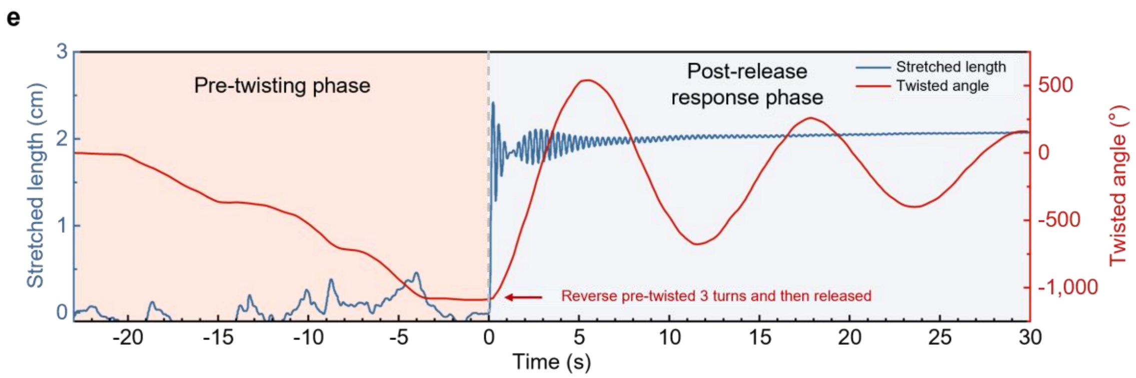

Fig. 5e, Real-time response curves of the actual stretched length and twisted angle of the objects.

9. The principles underlying the numerical model presented in Figure 5A are not clearly explained, and the methodological process remains ambiguous. Meanwhile, when the motion is a composite one, even if a certain offset in $\Delta R/R_0$ is observed, can the corresponding tensile values be clearly defined? For instance, as shown in Figures 3C and 3E, $\Delta C/C_0$ follows an approximately linear relationship in Figure 3C and a roughly quadratic relationship in Figure 3E. When these two effects are coupled, the resulting behavior should not be represented as a simple linear offset.

Response: We thank the reviewer for this insightful comment. The numerical model presented in revised Fig. 5a (corresponding to Fig. R12a, b) was constructed according to the following methodology and rationale: First, under predefined combined tensile and torsional strain conditions, we systematically acquired the variations in resistance and capacitance signals from the fibre sensor across a range of strain combinations (Fig. R12c). These data were subsequently subjected to nonlinear polynomial fitting and modeling analysis, yielding the final numerical model. Subsequently, the decoupling and perception of combined strains via this numerical model comprise two principal steps. Initially, given

that the resistance signal demonstrates specific sensitivity to tensile strain while remaining largely unaffected by torsional strain, the tensile strain can be accurately derived directly from $\Delta R/R_0$ (Fig. R12a). Subsequently, with the tensile strain thus determined, together with the measured $\Delta C/C_0$, the torsional strain can be further decoupled and quantified (Fig. R12b).

As rightly noted, $\Delta C/C_0$ in Fig. 3c exhibits a linear response to tensile strain, whereas $\Delta C/C_0$ in Fig. 3e follows an approximately quadratic relationship with torsional strain. Under coupled strain conditions, the relative capacitance variation can be effectively regarded as a superposition of the contributions from both strain components. Specifically, under a constant torsional strain, $\Delta C/C_0$ maintains a linear dependence on increasing tensile strain, as illustrated in Fig. R12c. This confirms that under combined deformation, the capacitive response retains additivity and separability, thereby furnishing a theoretical foundation for establishing the numerical model and enabling effective strain decoupling.

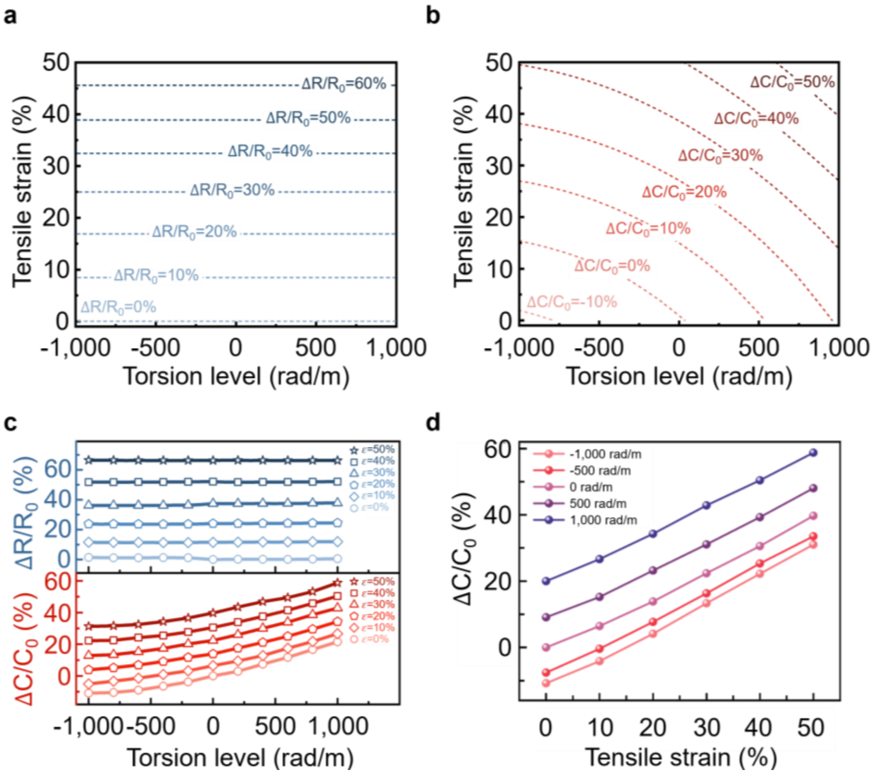

Fig. R12 | Interpretation of the dual-strain decoupling and sensing numerical model. a, Numerical simulation response of resistance signal under coupled tensile and torsional strain. **b,**

Numerical simulation response of capacitance signal under coupled tensile and torsional strain. **c,** Resistive and capacitive response of the fibre sensor (*h*=5 mm) measured under torsional strain at various fixed tensile strains. **d,** Linear capacitive response of the sensor to tensile strain under various fixed torsional strains.

In direct response to the lack of clarity highlighted, we have supplemented and refined the relevant methodological descriptions and theoretical explanations in the revised manuscript. These revisions provide a more lucid elaboration of the working mechanism of the dual-strain fibre sensor and the functional role of the numerical model in strain decoupling.

[On page 20] Calibration data quantifying the relative variations in resistance and capacitance under predefined tensile-torsional strain combinations (Fig. 4f) were employed to construct a numerical model. This model establishes the quantitative relationships between the sensor's electrical responses and the two strain modes (Fig. 5a). Leveraging this numerical model and capitalizing on the exclusive sensitivity of the resistive signal to tensile strain, the tensile strain can be directly and first quantified from the relative resistance change (Fig. 5a, left). Subsequently, the torsional strain can be decoupled by incorporating the quantified tensile strain together with the relative capacitive variation (Fig. 5a, right). This methodology enables accurate prediction of both tensile and torsional strain states, with the predicted values closely matching the predefined parameters (Fig. 5b).

10. The number of heavy-object samples tested in the experimental section is limited, and at a minimum, the relevant parameters of these objects should be reported. How do these parameters influence the test results?

Response: We thank the reviewer for this comment. In the composite strain decoupling and monitoring experiments, we conducted comprehensive tests using weights of varying

masses under diverse initial conditions. For the manuscript, we selected a representative trial employing a cylindrical weight of 50 g to clearly elucidate the sensor's working mechanism. As the reviewer correctly noted, the mass of the weight directly dictates the tensile stress applied to the sensor. Conversely, the inertial characteristics governing torsional oscillation are determined by the interplay of the weight's mass distribution (geometry) and the initial pre-twist angle. Collectively, these parameters dictate the dynamic mechanical response of the sensor upon release.

However, the primary objective of this work was to demonstrate the core functionality of the fabricated dual-strain fibre sensor: the real-time and accurate decoupling of tensile and torsional strains under composite loading. It is important to articulate that while varying load masses inevitably influence the magnitude and dynamic characteristics of the strain signals, the sensor's decoupling capability remains robust. This robustness stems from the inherent dual-signal synergistic sensing mechanism and the associated decoupling algorithm. In other words, within the operational strain range, the sensor consistently isolates and quantifies tensile and torsional components. The stability and accuracy of this decoupling process are primarily determined by the intrinsic properties of the sensing structure and the signal processing methodology, rather than the specific parameters of the applied load.

Nevertheless, we concur with the reviewer's perspective that future systematic investigations involving loads with varying masses, geometries, and moments of inertia would be valuable. Such studies would further comprehensively characterize the sensor's response boundaries and applicability across diverse scenarios. We thank the reviewer once again for their thorough and constructive critique, which has undoubtedly helped to enhance the rigor and completeness of our manuscript.

Response to Reviewer #3:

This manuscript reports a stretchable tri-channel fibre sensor with concentric and double-helical microchannels filled with gallium-based liquid metal, designed to decouple tensile and torsional deformations. The authors demonstrate enhanced stretchability and durability due to the helical chain orientation, and the sensor achieves dual resistive-capacitive sensing with good linearity and bidirectional torsional sensitivity. A digital twin platform is further developed for real-time monitoring and motion decoupling. However, in my opinion, the level of innovation is insufficient for publication in Nature Communications. Research on liquid metal-based fibre sensors is already extensive, and the present study mainly modifies the fibre structure without introducing a fundamentally new sensing mechanism or material concept. While the engineering optimization is carefully executed, the contribution does not substantially advance the state of the art.

Response: We extend our sincere gratitude to the reviewer for the meticulous evaluation and constructive suggestions, which have been instrumental in enhancing the quality of our manuscript and guiding our subsequent research directions. We also appreciate the reviewer's comment concerning the novelty of our work.

To more emphatically underscore the uniqueness and novelty of our work, the manuscript has been revised to provide a more emphatic elaboration of its key contributions. The novelty of our work is primarily demonstrated through three key aspects:

First, by combining theoretical analysis with experimental characterisation, we have validated for the first time that introducing a rotation process during fibre drawing process significantly enhances the mechanical properties of fibre. We find that rotating the preform during thermal drawing modulates the polymer chain orientation, inducing a helical alignment along the fibre axis, which yields increase from 805% to 1,918% in elongation at break and increase from 8.68 MPa to 15.91 MPa in tensile strength, thereby substantially

bolstering the mechanical robustness of both the fibres and the devices. This approach establishes a novel paradigm for advancing the performance and durability of stretchable fibre electronics.

Second, we engineered a high-performance dual-strain fibre sensor that exhibits exceptional performance metrics, including: (i) highly linear response to tensile strain, with $R^2 > 0.999$ over a broad range of 0-800%; (ii) wide bidirectional torsion sensing range of ±2000 rad/m; and (iii) superior durability exceeding 75,000 cycles at 200% tensile strain and 425,000 at ±1,000 rad/m torsional strain. Compared with other state-of-the-art multimodal strain sensors, our dual-strain fibre sensor demonstrates outstanding performance across critical metrics.

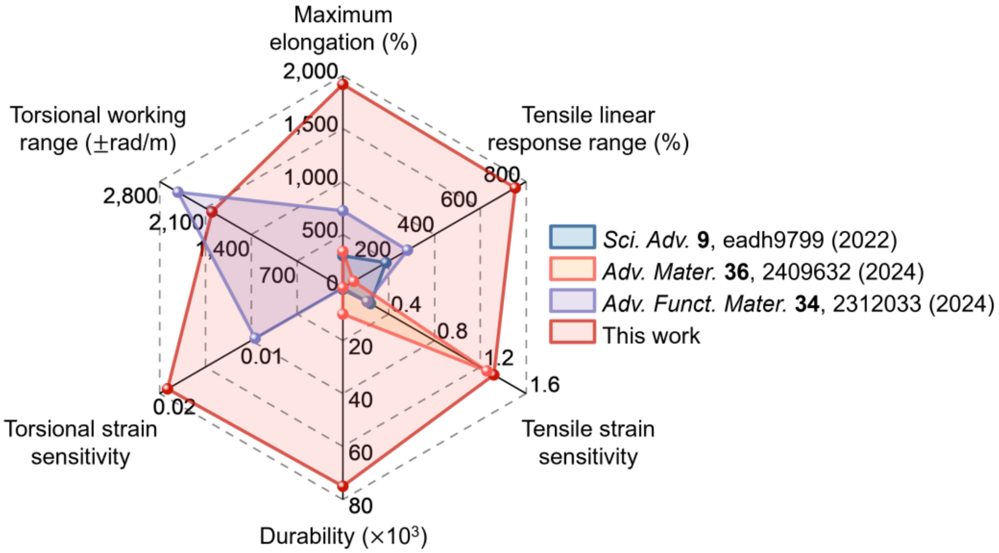

Fig. R13 | Comparison of the performance of the dual-strain fibre sensor with those state-of-the-art strain sensors in previous reports.

Third, we demonstrated a pioneering approach for the accurate, real-time decoupling and monitoring of composite tensile-torsional deformations using our dual-strain fibre sensor. This is achieved through a tri-channel fibre that integrates concentric straight and double-helical microchannel architectures. A dedicated algorithm leverages the differential

electrical responses of these distinct microchannels under tensile and torsional loading to achieve real-time decoupling and concurrent measurement of both strain components. This strategy effectively overcomes a key limitation of conventional strain sensors, which typically struggle to decouple multiple strain components under complex loading conditions. More importantly, it provides an unprecedented paradigm for deformation sensing in future fibre electronics and wearable devices destined for authentic, complex environments. This advancement significantly elevates the capabilities of fibre sensors in terms of multifunctional integration and robust signal decoupling.

All the points raised have been carefully addressed in the revised manuscript. We hope we clarify our work novelty and thank the reviewer again for the time and insightful comments, which have substantially strengthened the manuscript.

We have meticulously addressed all points raised by the reviewer, and corresponding revisions have been incorporated throughout the manuscript to better highlight our core innovations. We sincerely hope that our revisions and clarifications are now satisfactory and thank the reviewer once again for their time and insightful comments, which have substantially strengthened our manuscript.

1. The manuscript introduces a tri-channel fibre with two helical and one concentric conductive pathways. However, the necessity of incorporating three electrodes is not sufficiently justified. Since two electrodes could already accomplish capacitive sensing, the authors should clearly explain why three conductive channels are required and establish a detailed mathematical model of the double-helical capacitor.

Response: We thank the reviewer for this insightful comment. In this work, we propose a tri-channel fibre incorporating a concentric straight microchannel and double-helical microchannels. This distinctive architecture enables the simultaneous decoupling and real-time monitoring of superimposed tensile and torsional strains. Specifically, the concentric

straight channel outputs a resistive signal, while the double-helical channels output a capacitive signal. Under the application of a single strain type—either tensile or torsional—the fibre sensor exhibits robust and accurate responses, which can be directly monitored via the capacitance variations (Fig. R13a, b). However, when both strains are applied concurrently, the capacitive variation reflects a superposition of both strain components (lower panel of Fig. R13c).

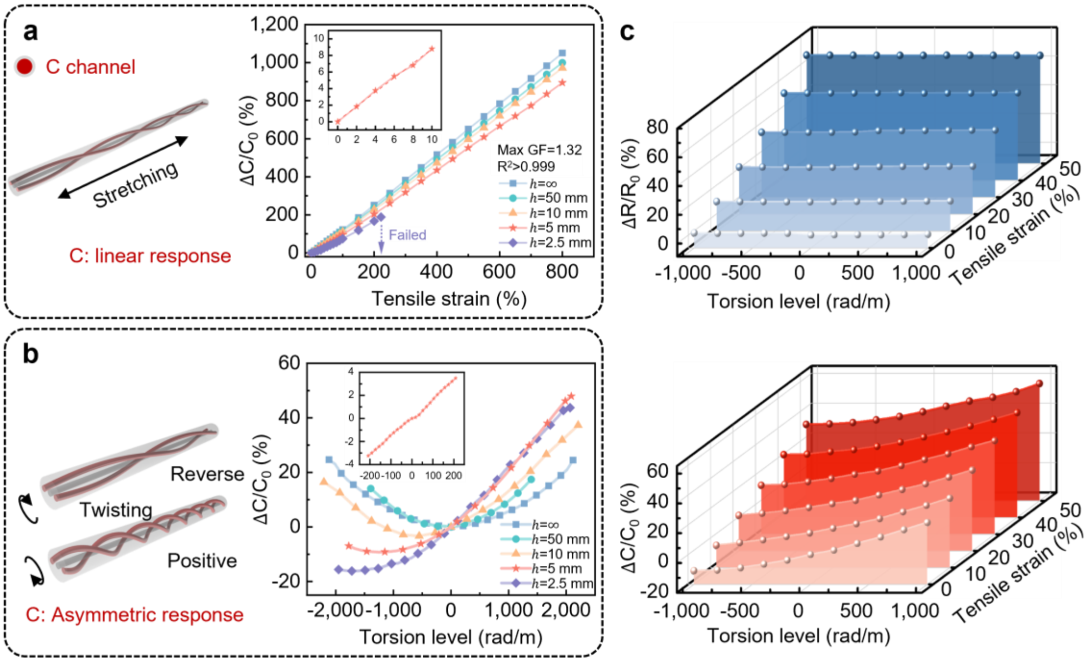

Fig. R13 | Challenges in decoupling and monitoring composite strain via capacitive channels alone. a, Schematic and corresponding response curve of the fibre sensor subjected to pure tensile strain. The inset shows the response at small tensile strains (from 0% to 10%). **b,** Schematic and corresponding response curve of the fibre sensor subjected to pure torsional strain. The inset shows the response at small torsional strains (from -200 rad/m to +200 rad/m). **c,** Resistive and capacitive response of the fibre sensor (h=5 mm) measured under torsional strain at various fixed tensile strains.

Theoretically, tensile strain (ε) and torsional strain (γ) can be decoupled from composite deformation by modeling the simultaneous resistance and capacitance responses

of the capacitive channel. Theoretical analysis reveals that the relative changes in resistance ($\Delta R/R_0$) and capacitance ($\Delta C/C_0$) can be expressed as functions of ε and γ, respectively:

$$\frac{\Delta R}{R_0}(\varepsilon,\gamma) = \frac{(1+\varepsilon)^3 + \left[\frac{\pi d_0}{h_0} + (1+\varepsilon)\frac{\gamma d_0}{2}\right]^2}{(1+\varepsilon)\left[1+\left(\frac{\pi d_0}{h_0}\right)^2\right]} - 1$$

$$\frac{\Delta C}{C_0}(\varepsilon,\gamma) = \sqrt{\frac{(1+\varepsilon)^3 + \left[\frac{\pi d_0}{h_0} + (1+\varepsilon)\frac{\gamma d_0}{2}\right]^2}{(1+\varepsilon)\left[1+\left(\frac{\pi d_0}{h_0}\right)^2\right]}}\frac{\ln\left(\frac{d_0}{2r_0} + \sqrt{\left(\frac{d_0}{2r_0}\right)^2 - 1}\right)}{\ln\left\{\frac{d_0}{2r_0}\left\{\frac{(1+\varepsilon)^3 + \left[\frac{\pi d_0}{h_0} + (1+\varepsilon)\frac{\gamma d_0}{2}\right]^2}{(1+\varepsilon)^3\left[1+\left(\frac{\pi d_0}{h_0}\right)^2\right]}\right\}^{1/4} + \sqrt{\left(\frac{d_0}{2r_0}\right)^2\left\{\frac{(1+\varepsilon)^3 + \left[\frac{\pi d_0}{h_0} + (1+\varepsilon)\frac{\gamma d_0}{2}\right]^2}{(1+\varepsilon)^3\left[1+\left(\frac{\pi d_0}{h_0}\right)^2\right]}\right\}^{1/2} - 1}\right\}} - 1$$

where d_0 is the initial distance between the centers of the two helical channels, h_0 is the initial helical period, and r_0 is the initial radius of the channel. Although theoretically feasible to determine ε and γ by simultaneously measuring $\Delta R/R_0$ and $\Delta C/C_0$, this approach encounters significant practical challenges. First, the disparity in response characteristics between resistance and capacitance signals to different strain modes can introduce substantial errors during equation solving. Second, simultaneous acquisition of both signals from a single channel may suffer from measurement crosstalk, circuit coupling, and noise interference, thereby compromising signal independence and decoupling accuracy. Consequently, relying solely on the capacitive channel for decoupling remains unreliable in practical applications.

To address these limitations, we implemented a robust strategy leveraging the unique property of the concentric straight channel: its resistance is insensitive to torsional strain and responds exclusively to tensile strain within the operating range (upper panel of Fig. R13c), as described by the following relationship:

$$\frac{\Delta R}{R_0}(\varepsilon,\gamma) = (\varepsilon+1)^2 - 1$$

Under composite strain conditions, this property allows the tensile strain component to be quantified independently and directly from the resistance change. Subsequently, the determined tensile strain is substituted into the capacitive response model as a known variable, enabling the accurate resolution of the corresponding torsional strain component.

This approach not only significantly reduces algorithmic complexity and real-time processing burdens, but also enhances system immunity to interference and overall robustness in dynamic environments through signal isolation and sequential solving. This strategy thereby ensures stable and reliable real-time dual-strain monitoring, providing an effective solution for decoupling measurements in complex mechanical environments.

As detailed in the Supplementary Information, we have formulated a mathematical model that describes the resistive response of the concentric straight microchannel to tensile strain and the capacitive response of the double-helical channels to both tensile and torsional strains (Fig. R15). A comprehensive analysis of the electrical responses as functions of the applied strains is provided in Supplementary Notes 4-6. This model quantitatively delineates the relationships between the variations in resistance and capacitance under applied tensile or torsional strain, thereby establishing the theoretical foundation for our decoupling algorithm.

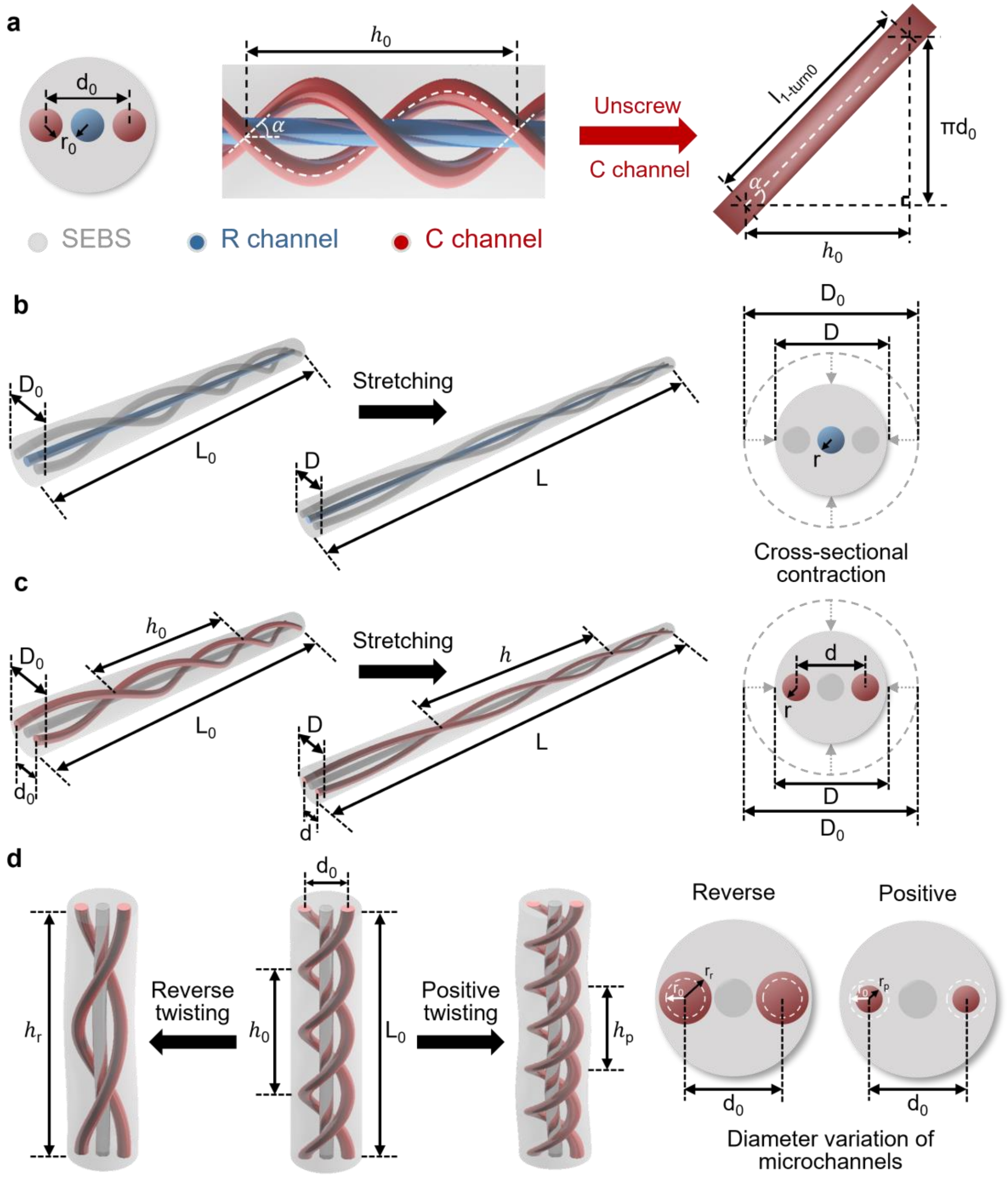

Fig. R15 | Working mechanism of the dual-strain fibre sensor for tensile and torsional strain sensing. a, The cross-section and side diagrams of the fibre strain sensor. The white dashed in the helical capacitance channel within the fibre highlights a single period of the helical channel and its unscrew version. **b,** Schematic diagram of resistance channel variation under tensile strain. **c,** Schematic diagram of capacitance channel variation under tensile strain. **d,** Schematic diagram of capacitance channel under torsional strain.

We have accordingly revised and supplemented the manuscript to provide a clearer exposition of the necessity of the tri-channel design for decoupling and monitoring composite strains.

[On page 14] When tensile and torsional strains are applied concurrently, the resistive signal responds exclusively to tensile strain while remaining insensitive to torsion. Conversely, variations in the capacitive signal arise from the synergistic influence of both strain types. Consequently, decoupling these combined loading conditions is unachievable by monitoring either the resistive or capacitive signal in isolation. This necessitates a combination approach: primarily quantifying the tensile strain via the resistive signal, and subsequently utilizing this data alongside the capacitive response to decouple and resolve the torsional strain (see Supplementary Note 6 for details). Thus, the synergistic utilization of both electrical sensing mechanisms is essential to not only distinguish individual strains, but also effectively decouple and monitor them under complex combined loading conditions.

2. In Fig. 4c, the response times are provided, but the corresponding applied stretching or torsional input times are not marked. The authors should indicate the precise timing of mechanical loading to ensure that the reported response times are clearly validated.

Response: We thank the reviewer for this insightful suggestion. We fully agree that clearly indicating the timing of applied strain is essential for accurately interpreting and verifying the sensor's dynamic response and recovery. Following this suggestion, we have incorporated explicit annotations into the original Fig. 4c. The revised Fig. 4c (corresponding to Fig. R16) clearly demarcates the application periods of both the 5% tensile strain and 300 rad/m torsional strain, thereby directly correlating these mechanical stimuli with the corresponding features in the sensor's electrical response profile.

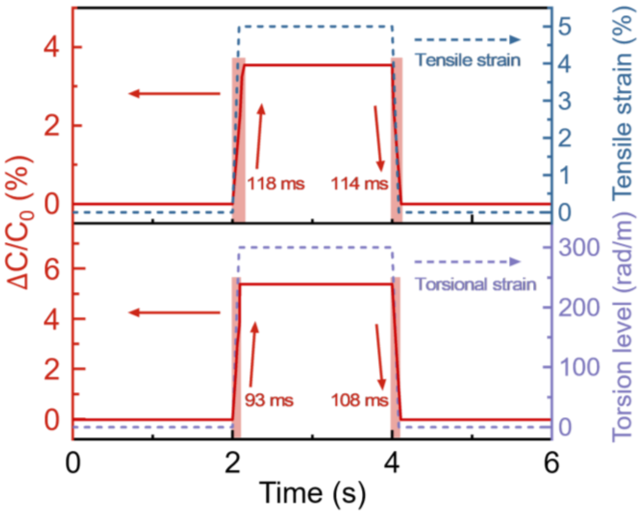

Fig. R16 | Response and recovery times of the sensor under 5% tensile and 300 rad/m torsional strains.

3. The maximum torsional angle that the fibre can withstand is not reported. The authors should specify the upper torsional limit and clarify at what angle the fibre undergoes transition from pure torsion to tensile deformation.

Response: We are grateful to the reviewer for this valuable observation. Under conditions of pure torsional strain, the applied torsion can be effectively monitored via corresponding variations in the capacitive signal. As demonstrated in Fig. 4e, the fibre sensor exhibits both accurate responsivity and fully recoverable cyclic performance within a defined torsional range. However, beyond a critical torsional threshold, excessive twisting can induce superimposed tensile deformation in addition to the primary torsional strain. In more severe instances, this leads to sensor "snarling" (see inset, Fig. R17a), thereby severely compromises the accurate perception of torsional strain. Fig. R17a delineates the maximum tolerable torsional strain prior to the onset of snarling under various applied tensile strains. These results indicate that the range of pure torsional response expands under larger applied tensile strains.

The decoupling and monitoring of superimposed tensile and torsional strains relies on a key structural feature: the concentric straight microchannel within the fibre responds

exclusively to tensile strain while remaining largely insensitive to torsion (exhibiting $\Delta R/R_0 < 1\%$ under pure torsional strain). As evidenced in Fig. R17b, under 0% tensile strain, the torsional strain exceedingly approximately ±1300 rad/m results in the $\Delta R/R_0$ value greater than 1%. This threshold corresponds, for instance, to about 20 full rotations in either direction for a 10-cm-long fibre, within which range the resistance change of the concentric straight channel remains negligible. This signifies that the concentric microchannel experiences superimposed tensile strain alongside the applied torsion. Consequently, under 0% tensile strain, torsional strains beyond ±1300 rad/m introduce non-negligible tensile components, thereby compromising the accuracy of composite strain decoupling.

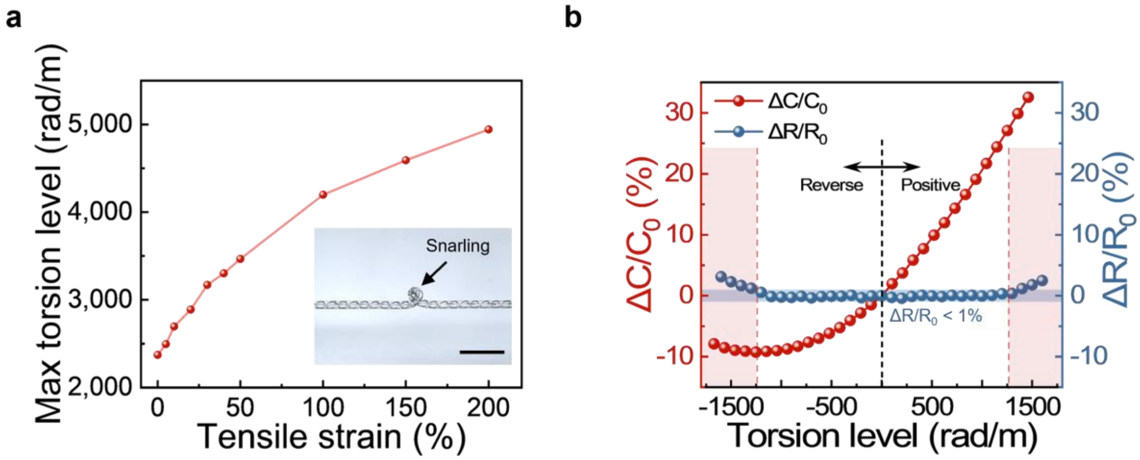

Fig. R17 | Maximum tolerable torsional strain range of the fibre sensor. a, The relationship between the maximum torsion level and the tensile strain of the fibre strain sensor. The insert shows a photograph of a highly twisted fibre strain sensor forming plectonemes at a snarling point (scale bar, 5 mm). b, Resistance and capacitance responses of the fibre sensor (h=5 mm) under pure torsional strain.

To ensure stable decoupling, robust recovery, and long-term reliability under composite strain, we define the effective operational range under composite strain as follows: tensile strain ≤ 50% and torsional strain within the range of –1000 to +1000 rad/m. This operational range has been validated through composite strain decoupling

experiments, ensuring accurate and stable dual-mode signal output along with real-time decoupling within the recoverable deformation regime (as shown in Fig. 5).

We thank the reviewer again for their thorough review. In response to these comments, we have revised the manuscript accordingly, including a more detailed discussion on the maximum tolerable torsional strain range. These modifications have enhanced the overall rigor and clarity of the work.

[On page 14] Notably, when torsional strain exceeds a critical threshold, excessive torsion may induce additional tensile deformation alongside the primary torsional deformation. This deformation alters the geometry of the concentric resistance channel, resulting in the $\Delta R/R_0$ being unable to remain within 1%. As evidenced in Fig. 3e, the resistance signal exhibits a slight increase under high magnitudes of reverse torsional strain. More critically, this can lead to snarling (Supplementary Fig. 14c), which severely compromises the accurate perception of torsional strain.

[On page 17] To ensure decoupling stability and long-term reliability under combined strain, the effective operational range of the sensor was confined to tensile strains $\leqslant$ 50% and torsional strains within ±1000 rad/m.

4. The mechanical and electrical hysteresis behaviour of the fibre sensor should be systematically characterised, as hysteresis is critical for evaluating reliability and long-term sensing performance.

Response: We thank the reviewer for raising this insightful comment. In response, we have systematically supplemented and analysed the mechanical and electrical hysteresis properties of the fibre sensor, with the results summarized in Fig. R18. As shown in Fig. R18a, the elastic performance of the fibre was evaluated under progressively increasing tensile strains ranging from 100% to 500%. Throughout this strain range, the resultant stress-strain curves exhibit characteristic hysteresis, and the area of the hysteresis loop

increases proportionally with the applied tensile strain. Furthermore, under cyclic loading at a fixed 100% tensile strain (Fig. R18b), the mechanical hysteresis loop area was maximized during the initial cycle. This phenomenon is primarily attributed to energy dissipation arising from chain slippage and internal friction during the straining process, combined with the reorganization and dissipation within the microscopic two-phase structure following the initial large deformation [*Adv. Sci.* 2025, 12, e05417]. In subsequent cycles, the loop area gradually diminished and subsequently stabilized. This trend suggests that the elastic network progressively attained a dynamic equilibrium following repeated stretching-recovery cycles, resulting in a more reversible mechanical response.

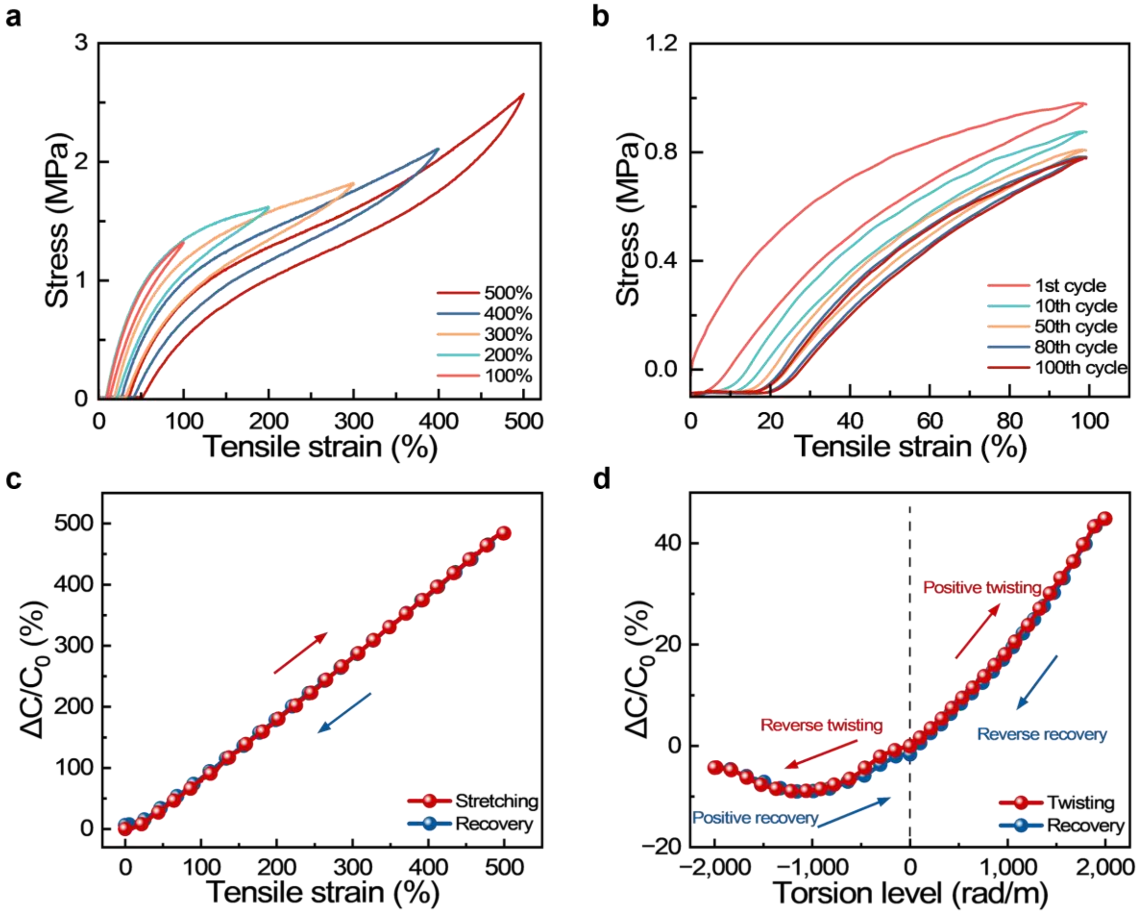

Fig. R18 | Mechanical and electrical hysteresis characteristics of fibre sensors. a, Stress-strain curves of the fibre with helical microchannels (h = 5 mm) under varying tensile strains. **b,** Cyclic loading-unloading curves of the fibre with helical microchannels (h = 5 mm) at a fixed strain of

100% over 100 cycles. **c,** Electrical hysteresis of the fibre sensor under 500% tensile strain. **d,** Electrical hysteresis under torsional strain ranging from –2000 to 2000 rad/m.

Following multiple strain-recovery steps, the electrical hysteresis curves under both tensile and torsional strain (Fig. R18c, d) reveal substantial overlap between the signal responses during the loading and unloading processes. This indicates excellent signal consistency and repeatability during dynamic measurements, indicating negligible electrical hysteresis. Furthermore, the cyclic stability and operational lifespan of the sensor were evaluated under varying tensile strains, as summarized in Fig. R19. Even at 200% tensile strain, the fibre sensor maintains stable cyclic operation over 75,000 cycles.

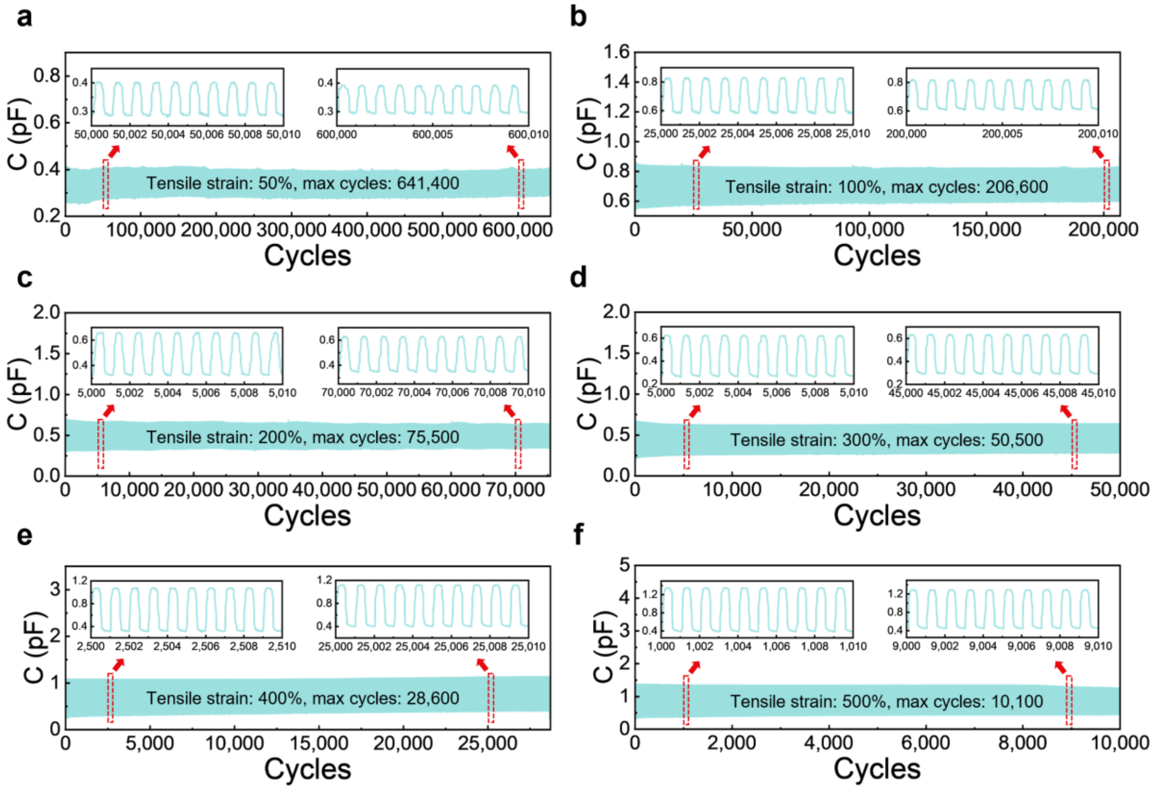

Fig. R19 | Cyclic stability and service life testing of fibre sensors under different strains. a, Under 50% cyclic tensile strain, maximum durability cycles reach 641,400. **b,** Under 100% cyclic tensile strain, maximum durability cycles reach 206,600. **c,** Under 200% cyclic tensile strain, maximum durability cycles 75,500. **d,** Under 300% cyclic tensile strain, maximum durability cycles

reach 50,500. **e,** Under 400% cyclic tensile strain, maximum durability cycles reach 28,600. **f,** Under 500% cyclic tensile strain, maximum durability cycles reach 10,100.

In summary, the fibre sensor exhibits inherent hysteresis in both its mechanical and electrical responses. However, following a few conditioning strain-recovery cycles, both mechanical and electrical properties rapidly stabilize. This characteristic signifies a highly stable internal structure and reliable signal output, thereby rendering the sensor suitable for dynamic sensing applications that require long-term, stable monitoring. We have revised and supplemented the relevant sections of the manuscript and supplementary information to provide a clearly analysis and explanation of the mechanical and electrical hysteresis characteristics of the fibre sensor.

[On page 11] The mechanical hysteresis behaviour of the tri-channel fibre was further investigated across various tensile strain levels and under cyclic loading, demonstrating excellent elastic recovery and durability (Supplementary Fig. 9-11).

[On page 17] Furthermore, the electrical hysteresis behaviour of the sensor was investigated (Supplementary Fig. 19). Following multiple strain cycles, the electrical hysteresis curves for both tension and torsion exhibited substantial overlap between the loading and unloading phases. This overlap signifies excellent signal consistency and repeatability, confirming negligible electrical hysteresis during dynamic measurements.

5. A comparative study is needed on fibres with different helical periods, specifically regarding their maximum stretchable length. In addition, the authors should report the elongation at break before and after liquid metal filling, to clarify the influence of structural design and filler on the mechanical robustness.

Response: We appreciate the reviewer for this valuable comment. To address it, we performed tensile stress-strain measurements on fibres with varying helical periods (h). The resultant data, presented in Fig. R20a, reveal that the ultimate elongation of the fibres

does not follow a simple negative correlation with the h. Specifically, the measured fracture strains are 805% (h=∞), 891% (h=50 mm), 1,076% (h=25 mm), 1,578% (h=10 mm), 1,918% (h=5 mm), 1,222% (h=2.5 mm). This phenomenon originates from the interplay between microscopic chain orientation and macroscopic structural features. At a moderate h, the tilt angle δ between the polymer chains and the fibre axis reaches an optimal value. This configuration facilitates effective energy dissipation through chain uncoiling during stretching, while maintaining relatively mild stress concentration within the helical channels, thereby collectively contributing to significantly enhanced toughness. However, when h is excessively small, the resulting large tilt angle δ reduces the axial load-bearing capacity. Concurrently, the high density of helical channels induces pronounced stress concentration, promoting localized deformation and accelerating defect propagation. These factors collectively lead to a reduction in the elongation at break. This behaviour fundamentally reflects a trade-off between the toughening effect conferred by the helical orientation and the degradation in load-bearing capacity induced by an excessively small helical period.

To further elucidate the influence of the microchannel filler on mechanical performance, we characterized the tensile stress-strain response of the tri-channel fibre infused with liquid metal (Fig. R20b). The results demonstrate a slight reduction in the fibre's elongation at break but a corresponding increase in its fracture strength upon liquid metal infusion. This change stems from two competing mechanisms: on one hand, the infiltration of liquid metal may accelerate the propagation of microcracks initiating within the fibre at high strains, leading to a premature fracture [*Nat. Electron.* 2025, 8, 1072-1081]; on the other hand, the liquid metal filler may enhance the fibre's load-bearing capacity through interfacial interaction and local stress redistribution, which manifests as the observed increase in fracture strength [*Adv. Mater.* 2025, 37, 2415268]. Collectively, this indicates that employing liquid metal as a filler material does not substantially alter the

mechanical behaviour of the fibre, underscoring that structural design and microscopic polymer chain orientation remain the dominant factors governing its tensile properties. We have accordingly refined the discussion in the revised manuscript and Supplementary information to more clearly delineate the distinct roles of structural parameters and filler materials in determining the fibre's mechanical robustness.

[On page 11] Tri-channel fibres with helical periods varying from 2.5 to 50 mm consistently exhibit enhanced fracture elongation and tensile strength compared to their counterparts with $h = \infty$ (Supplementary Fig. 9a). This enhanced mechanical behaviour arises from the specific chain orientation and structural configuration, which strengthen the intermolecular interactions and the material's capacity for energy dissipation during deformation (see Supplementary Note 3 for details).

[On page 12] In addition, the effect of liquid metal infusion into the microchannels on the mechanical properties of the fibres was examined (Supplementary Fig. 9b). The results indicate that the high fluidity of the liquid metal allows it to accommodate fibre deformation through flow within the microchannels, thus exerting only a small influence on the intrinsic mechanical properties of the fibre matrix.

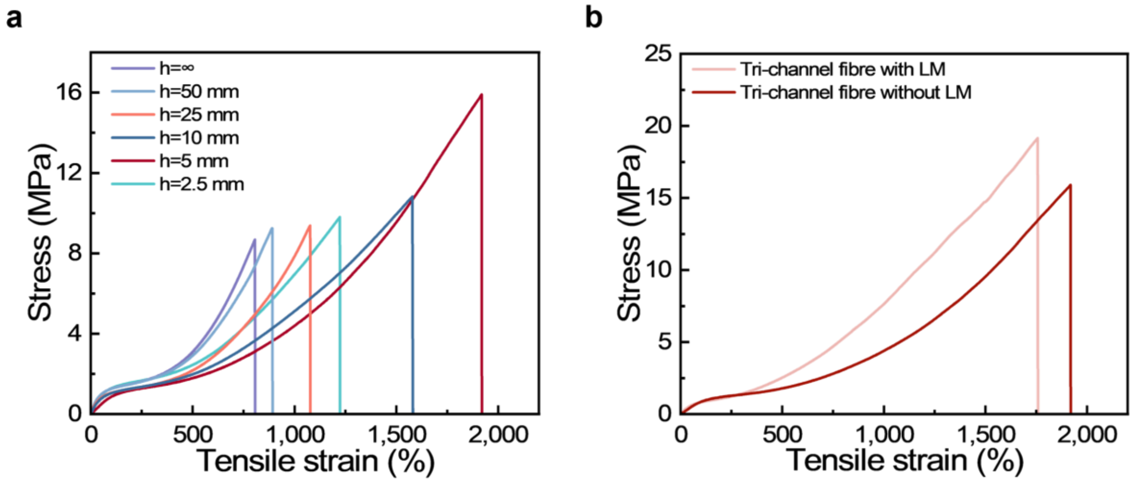

Fig. R20 | Stress-strain curves of the different type of tri-channel fibres. a, Stress−strain curves for tri-channel fibres with different h. **b,** Comparison of stress–strain curves for tri-channel fibres (h=5 mm) with and without liquid metal filling the microchannels.

Response to Reviewer #1:

Thank you for addressing the comments and providing the corresponding clarifications. The tri-channel fiber geometry appears to be an effective approach for decoupling strain and torsion, and I was particularly impressed by the close agreement between the theoretical predictions and the experimental results. I also find the approach of treating the change in resistance as a known quantity and incorporating it into the capacitance model to be a practical and well-justified strategy for real-world applications.

Response: We sincerely thank the reviewer for the positive evaluation and encouraging remarks regarding our revised manuscript. We are delighted to learn that the clarifications provided have satisfactorily addressed your previous concerns. We greatly appreciate your recognition of the tri-channel fibre geometry as an effective solution for decoupling strain and torsion, as well as your endorsement of our modeling strategy involving resistance and capacitance. Your constructive feedback throughout this process has significantly improved the quality and clarity of our work. We are grateful for your time and the insightful perspective you have brought to this study.

1. Out of curiosity, I had one question regarding the distinction between the equation presented on page 9 of the response and the change-in-resistance equation in Supplementary Note 4. Were these equations derived using different assumptions or methods, and if so, what prevents the equation in the supplementary information from being used in the proposed practical application strategy?

Response: We thank the reviewer for this comment. We would like to clarify that the equation presented on page 9 of our previous response and the change-in-resistance equation detailed in Supplementary Note 4 were indeed **derived using distinct assumptions and methodologies**. Specifically, as discussed on Page 9 of our previous

response, we explored a theoretical scenario for decoupling composite tensile (ε) and torsional (γ) strains by relying solely on the resistance and capacitance signals from the internal helical channels (i.e., the capacitive channels). Whereas in Supplementary Note 4, we present the practical decoupling strategy that leverages our unique tri-channel fibre architecture. This method utilizes the resistance signal from the concentric straight channel (which is insensitive to torsional strain) in conjunction with the capacitance signal from the double helical microchannels to synergistically perceive and decouple composite strains. This strategy significantly streamlines the decoupling process and enhances measurement accuracy. The resistance response of the concentric straight channel under combined tensile and torsional strains can be simplified as follows (also presented on Page 9 of our previous response):

$$\frac{\Delta R}{R_0}(\varepsilon, \gamma) = (\varepsilon + 1)^2 - 1$$

This expression is consistent with the resistance response equation **under pure tensile strain** derived in **Supplementary Note 4**. Furthermore, **Supplementary Note 6** provides a comprehensive derivation of the resistance and capacitance responses **under combined strain conditions**. **Consequently, for the practical implementation involving composite deformation, we employed the theoretical model established in Supplementary Note 6.** We have revised this section to improve readability and clarity, and we hope these explanations satisfactorily address your concerns.

[Supplementary Information, Page 11]

Supplementary Note 4 | Calculation of the resistive and capacitive response of the dual-strain fibre sensor under pure tensile strain.

Therefore, when subjected only to tensile strain ε, the resistance $R(\varepsilon)$ and its relative changes $\Delta R/R_0(\varepsilon)$ as measured by the resistance channel, are given by the following expressions:

$$R(\varepsilon) = \rho \frac{L}{\pi r^2} = \rho \frac{(1+\varepsilon)^2 L_0}{\pi {r_0}^2} \tag{6}$$

$$\frac{\Delta R}{R_0}(\varepsilon) = (\varepsilon + 1)^2 - 1 \tag{7}$$

[Supplementary Information, Page 16]

Supplementary Note 6 | Real-time monitoring and computational decoupling of simultaneous tensile and torsional strains using the dual-strain fibre sensor.

When the fibre strain sensor is subjected to both tensile and torsional strains simultaneously, the resistive signal responds exclusively to tensile strain, remaining insensitive to torsion; in contrast, the capacitive response arises from the superposition of both stimuli. Consequently, the resistance $R(\varepsilon, \gamma)$, capacitance $C(\varepsilon, \gamma)$ and their relative change $\frac{\Delta R}{R_0}(\varepsilon, \gamma)$ and $\frac{\Delta C}{C_0}(\varepsilon, \gamma)$ under composite tensile strain ε and torsion level γ are described by the following relationships:

$$R(\varepsilon, \gamma) = \rho \frac{L}{\pi r^2} = \rho \frac{(1+\varepsilon)^2 L_0}{\pi {r_0}^2} \tag{24}$$

$$\frac{\Delta R}{R_0}(\varepsilon, \gamma) = (\varepsilon + 1)^2 - 1 \tag{25}$$

$$C(\varepsilon, \gamma) = \frac{\pi \epsilon L_0 \sqrt{\dfrac{(1+\varepsilon)^3 + \left[\dfrac{\pi d_0}{h_0} + (1+\varepsilon)\dfrac{\gamma d_0}{2}\right]^2}{(1+\varepsilon)}}}{\ln\left\{\dfrac{d_0}{2r_0}\left\{\dfrac{(1+\varepsilon)^3 + \left[\dfrac{\pi d_0}{h_0} + (1+\varepsilon)\dfrac{\gamma d_0}{2}\right]^2}{(1+\varepsilon)^3\left[1 + \left(\dfrac{\pi d_0}{h_0}\right)^2\right]}\right\}^{\frac{1}{4}} + \sqrt{\left(\dfrac{d_0}{2r_0}\right)^2\left\{\dfrac{(1+\varepsilon)^3 + \left[\dfrac{\pi d_0}{h_0} + (1+\varepsilon)\dfrac{\gamma d_0}{2}\right]^2}{(1+\varepsilon)^3\left[1 + \left(\dfrac{\pi d_0}{h_0}\right)^2\right]}\right\}^{\frac{1}{2}} - 1}\right\}} \tag{26}$$

$$\frac{\Delta C}{C_0}(\varepsilon, \gamma) = \sqrt{\frac{(1+\varepsilon)^3 + \left[\dfrac{\pi d_0}{h_0} + (1+\varepsilon)\dfrac{\gamma d_0}{2}\right]^2}{(1+\varepsilon)\left[1 + \left(\dfrac{\pi d_0}{h_0}\right)^2\right]}} \frac{\ln\left(\dfrac{d_0}{2r_0} + \sqrt{\left(\dfrac{d_0}{2r_0}\right)^2 - 1}\right)}{\ln\left\{\dfrac{d_0}{2r_0}\left\{\dfrac{(1+\varepsilon)^3 + \left[\dfrac{\pi d_0}{h_0} + (1+\varepsilon)\dfrac{\gamma d_0}{2}\right]^2}{(1+\varepsilon)^3\left[1 + \left(\dfrac{\pi d_0}{h_0}\right)^2\right]}\right\}^{\frac{1}{4}} + \sqrt{\left(\dfrac{d_0}{2r_0}\right)^2\left\{\dfrac{(1+\varepsilon)^3 + \left[\dfrac{\pi d_0}{h_0} + (1+\varepsilon)\dfrac{\gamma d_0}{2}\right]^2}{(1+\varepsilon)^3\left[1 + \left(\dfrac{\pi d_0}{h_0}\right)^2\right]}\right\}^{\frac{1}{2}} - 1}\right\}} - 1$$

$$= \lambda\sqrt{Z}\frac{\ln\left(A + \sqrt{A^2 - 1}\right)}{\ln\left\{AZ^{\frac{1}{4}} + \sqrt{A^2 Z^{\frac{1}{2}} - 1}\right\}} - 1 \tag{27}$$

Where $\lambda = (1+\varepsilon)$ is a parameter related to the tensile strain, $\beta = \frac{\gamma h_0}{2\pi}$ is a parameter related to the torsion level, and $Z = \frac{(1+\varepsilon)^3 + \left[\frac{\pi d_0}{h_0} + (1+\varepsilon)\frac{\gamma d_0}{2}\right]^2}{(1+\varepsilon)^3\left(1+\left(\frac{\pi d_0}{h_0}\right)^2\right)} = \frac{\lambda^3 + \left[\frac{\pi d_0}{h_0}(1+\lambda\beta)\right]^2}{\lambda^3\left[1+\left(\frac{\pi d_0}{h_0}\right)^2\right]}$.

Response to Reviewer #2:

In the previous response, the author has addressed the reviewer's comments, but some issues still remain to be resolved.

1. When addressing whether the pre-torsion step could affect the measurement results, the authors' statement that the sensor response is "independent of external forces" is overly absolute, since sensor strain is inherently induced by external loading and the decoupling relies on strain signals. Please clarify whether different loading rates (dynamic impact vs. quasi-static) could alter the response due to material viscoelasticity and liquid-metal inertia. The experiments currently cover only quasi-static and low-frequency oscillations, without validation under dynamic loading. In addition, how is the pre-torsion level determined? Is the same pre-torsion angle applied across different load masses, or is it adjusted depending on the load?

Response: We are grateful to the reviewer for raising these critical points regarding sensor dynamics and the pre-torsion procedure. Accordingly, we have revised the manuscript to comprehensively address these concerns, as detailed below.

(1) Clarification on "independence of external forces"

We acknowledge the reviewer's valid point that our initial phrasing was not sufficiently precise. We have refined the text to clarify that while the sensor's instantaneous electrical response is intrinsically determined by the fibre's strain states, the temporal evolution of strains is strictly governed by the external loading conditions. Specifically, the magnitude of the suspended mass directly determines the tensile stress applied to the sensor. Simultaneously, the torsional oscillation dynamics are defined by the interplay between the mass's moment of inertia (dependent on geometry) and the initial pre-twist angle. Collectively, these parameters dictate the dynamic mechanical behaviour of the sensor upon release.

[Manuscript, Page 20]

Initially, a weight was suspended from the lower end of the dual-strain fibre sensor, maintaining the sensor in its original length state and pre-twisted. While the mass of the suspended weight governs the tensile response profile, the torsional response is determined by the interplay between the object's moment of inertia and the initial pre-twist angle. Upon release, the suspended mass induced simultaneous axial elongation and torsional recovery, generating oscillatory deformation cycles.

(2) Influence of loading rates on sensor response

We appreciate the reviewer's insightful query regarding the dependence of the sensor response on loading rates. We acknowledge that the viscoelastic nature of the SEBS elastomer and the inertial properties of the liquid metal could theoretically influence the sensor response, particularly under high-strain-rate regimes. **However, our results demonstrate that across a broad strain range investigated under quasi-static conditions (Figs. 4d and 4e), the sensor maintains exceptional consistency and real-time responsiveness. This robust performance is attributed to the rapid elastic recovery of the elastomeric matrix and the high fluidity of the liquid metal. Furthermore, experimental validation confirms that within a defined range of loading rates, both the response frequency and the response characteristics remain consistent (Supplementary Fig. 22).** Nevertheless, at significantly elevated loading rates, the viscoelastic relaxation times and fluid inertial effects may become non-negligible, potentially inducing signal hysteresis or compromising measurement accuracy.

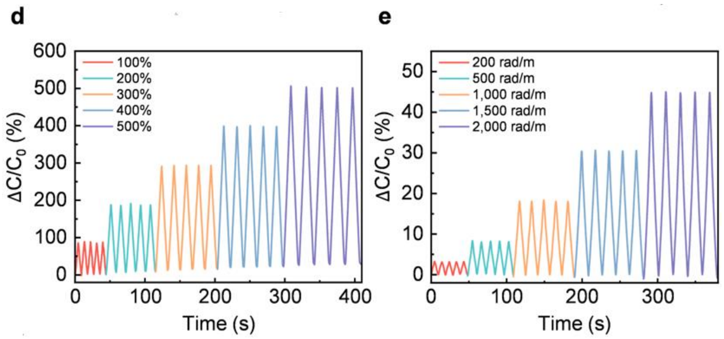

Fig. 4 | Electromechanical performance of dual-strain fibre sensor. d, Capacitive response of the sensor under varying tensile strains (100–500%). **e,** Capacitive response of the sensor under varying torsional strain (200–2,000 rad/m).

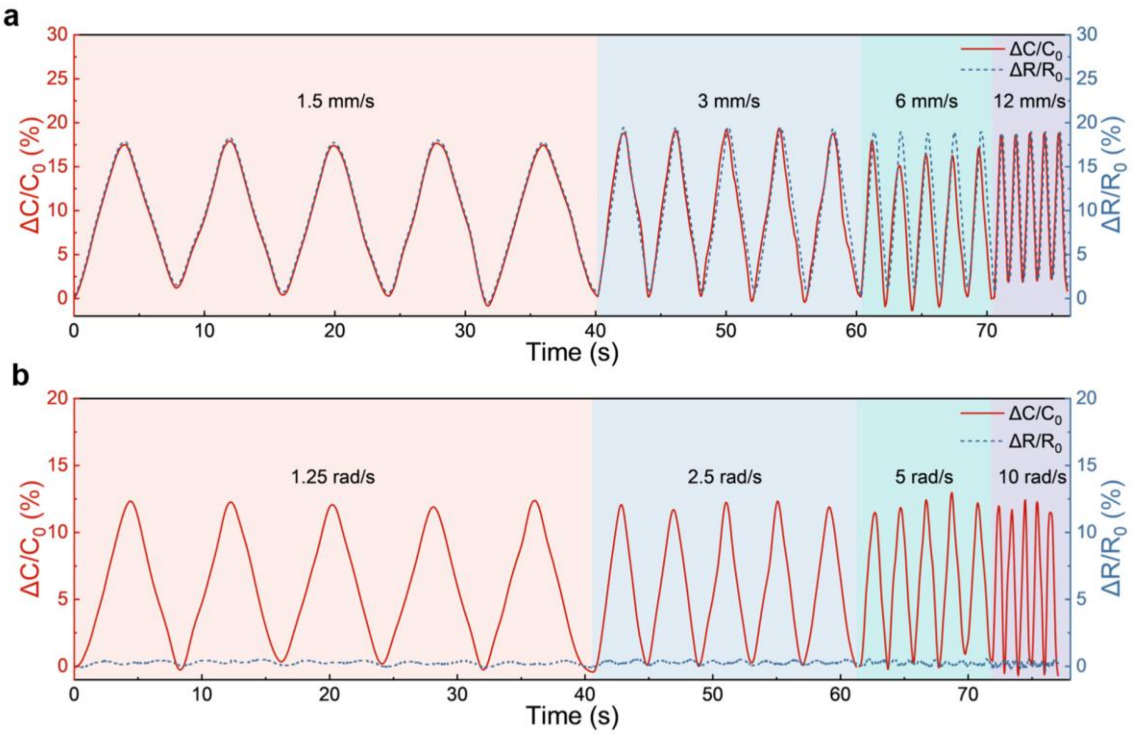

Supplementary Fig. 22 | Resistive and capacitive response curves of the dual-strain fibre sensor under tensile and torsional strains at various loading rates. a, Relative changes in resistance and capacitance of the fibre strain sensor under tensile strain at loading rates of 1.5, 3, 6, and 12 mm/s. **b,** Relative changes in resistance and capacitance of the fibre strain sensor under torsional strain at loading rates of 1.25, 2.5, 5, and 10 rad/s.

The present study focuses on characterizing quasi-static and low-frequency oscillatory regimes, which encompass most human-motion and soft-robotic applications. This comment provides valuable guidance for future investigations to systematically probe the dynamic response limits and incorporate inertial compensation models for applications requiring high-frequency sensing. To address the reviewer's concern, we have included the following discussion in the revised manuscript:

[Manuscript, Page 18]

Attributable to the rapid elastic recovery of the elastomeric matrix and the inherent fluidity of the liquid metal, the sensor exhibits high signal consistency and real-time responsiveness across various loading rates (Supplementary Fig. 22), thereby satisfying the demands of most quasi-static and low-frequency dynamic oscillatory sensing scenarios.

[Supplementary Information, Page 40]

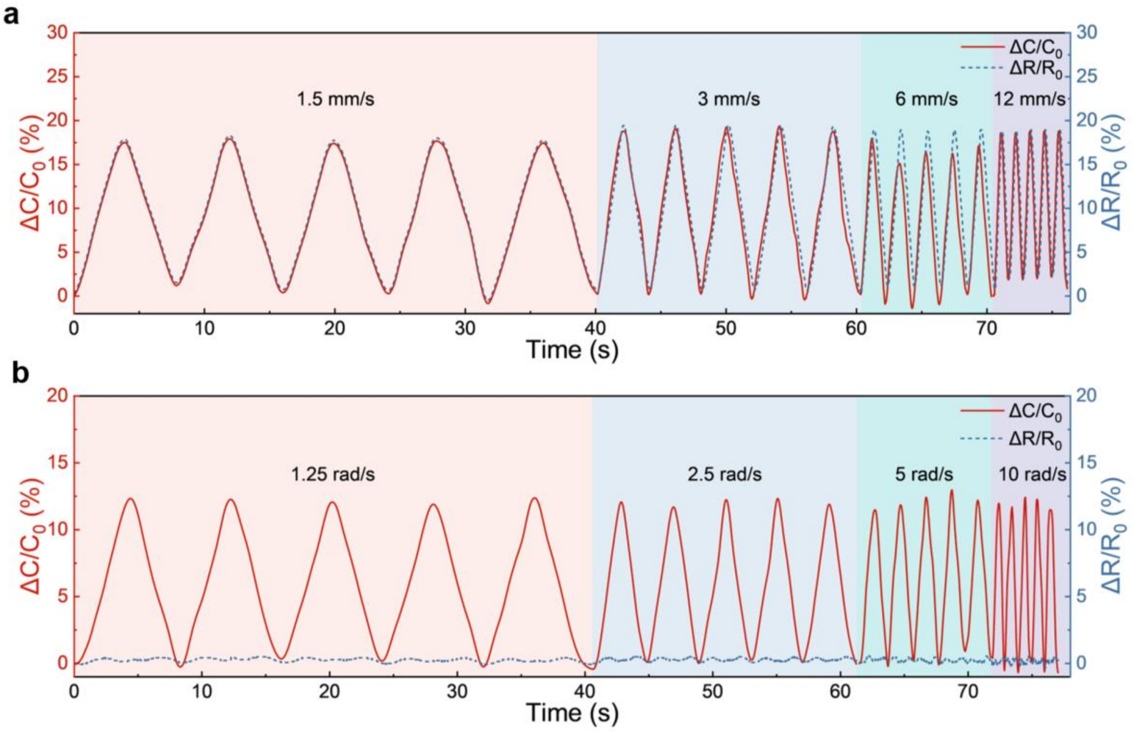

Supplementary Fig. 22 | Resistive and capacitive response curves of the dual-strain fibre sensor under tensile and torsional strains at various loading rates. a, Relative changes in resistance and capacitance of the fibre strain sensor under tensile strain at loading rates of 1.5, 3, 6, and 12 mm/s. **b,** Relative changes in resistance and capacitance of the fibre strain sensor under torsional strain at loading rates of 1.25, 2.5, 5, and 10 rad/s.

(3) Rationale for the selection of pre-torsion parameters

In the demonstration of simultaneous monitoring and decoupling of combined tensile and torsional strains (Fig. 5c-e), pre-torsion was applied to impart initial torsional potential

energy, ensuring that the mass-induced motion comprised both tensile and torsional components. To ensure consistency across experiments with varying load masses, a constant pre-torsion turns was maintained (three full turns for the results shown in Fig. 5). While these specific parameters served as representative examples, we have expanded our validation in Supplementary Figs. 25 and 26. These figures present decoupling results under varying pre-torsion turns with constant load mass, and conversely, varying load masses with constant pre-torsion turns, thereby demonstrating the universality of our approach.

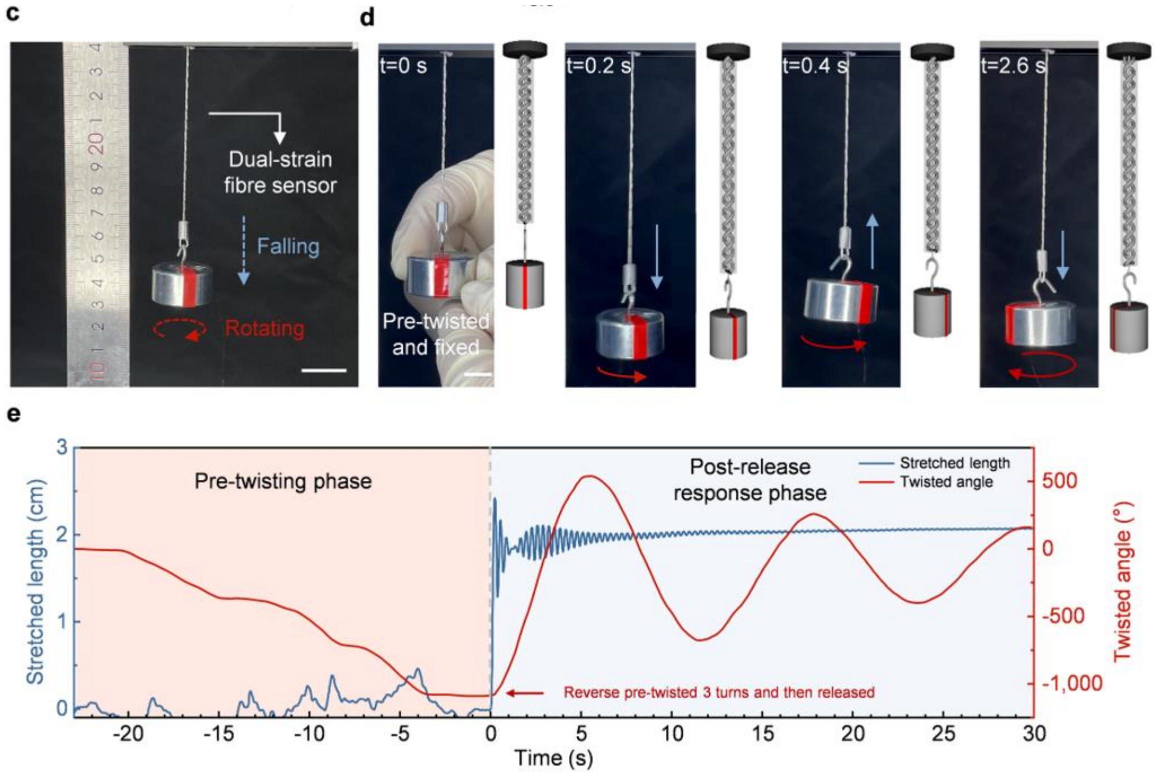

Fig. 5 | Composite motions decoupling performance of the dual-strain fibre sensor. c, Experimental platform for simultaneous monitoring and discrimination of tensile and torsional strains (scale bar, 2 cm). **d,** Digital twin model reflecting the real-time motion states of the objects (scale bar, 1 cm). **e,** Real-time response curves of the actual stretched length and twisted angle of the objects.

The experimental observations indicate that, **<u>under a constant load mass, increasing the pre-torsion turns predominantly influences the system's torsional response</u>** (Supplementary Fig. 25). Specifically, the initial amplitude of torsional oscillation increases significantly, whereas the oscillation period remains largely unaffected. Conversely, the impact on tensile oscillation is marginal; the tensile strain continues to oscillate within a comparable range. This behaviour is attributed to the fact that pre-torsion primarily alters the initial torsional potential energy of the system with negligible impact on axial stiffness within an appropriate torsional range, thereby preserving the stability of the tensile response dynamics.

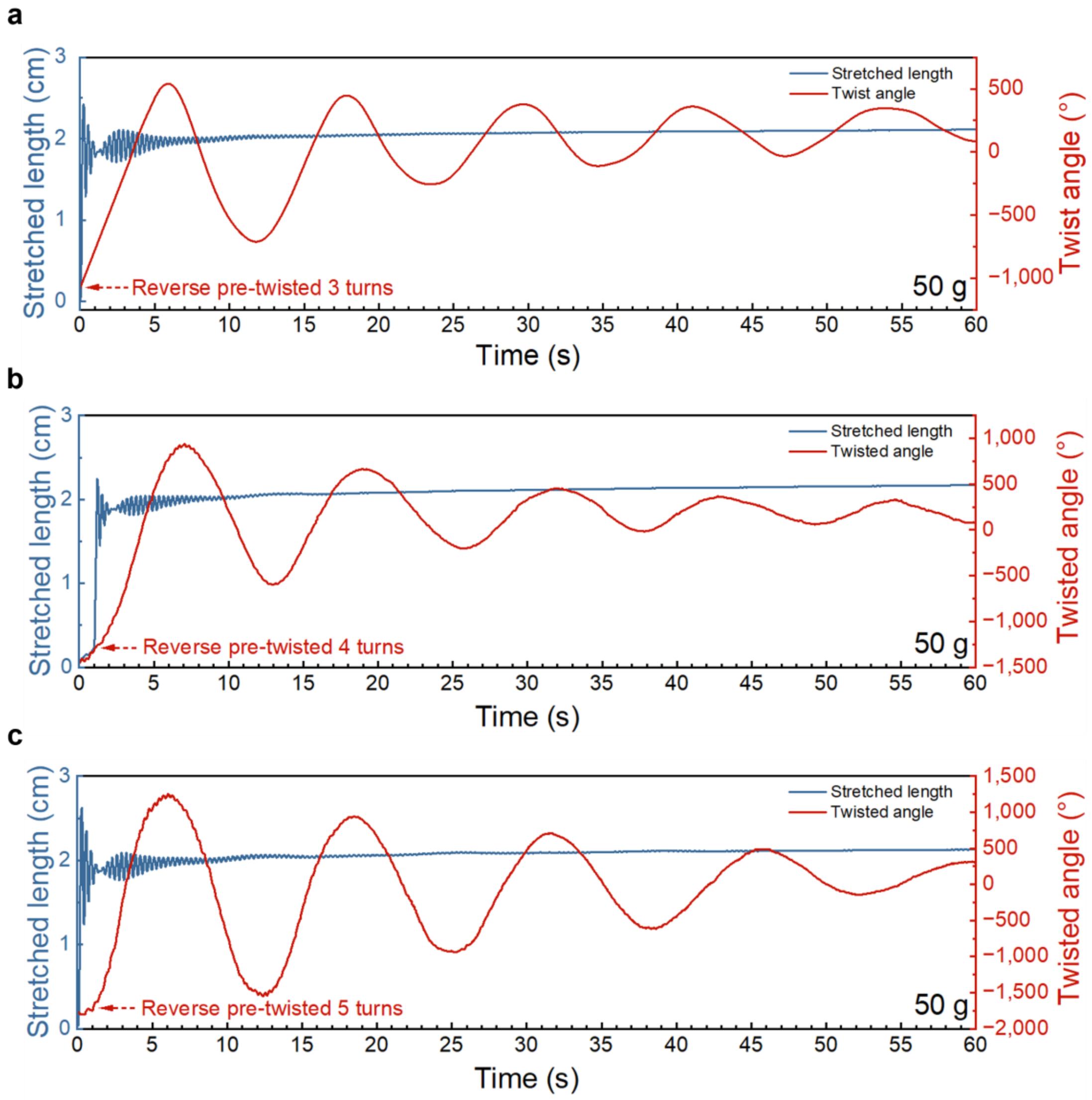

Supplementary Fig. 25 | Real-time decoupling performance under constant load and varying pre-twist turns. a–c, Decoupled information with pre-twist of 3 turns **(a)**, 4 turns **(b)**, and 5 turns **(c)**.

In contrast, **under a constant pre-torsion angle, varying the load mass significantly alters the dynamic response characteristics for both tension and torsion** (Supplementary Fig. 26). As the load mass increases, the tensile equilibrium length extends, and the total duration of oscillation is prolonged. Notably, under low-mass conditions (e.g., 10 g), the system exhibits a slow elastic recovery, characterized by a gradual decrease in equilibrium length during the oscillation process (Supplementary Fig. 26a). This phenomenon arises because, with lower mass, the inertial forces are reduced, making damping effects and the fibre's viscoelastic relaxation behaviour more pronounced in the macroscopic response. Regarding torsional oscillation, while the initial amplitude remains substantially constant as mass increases, the oscillation period lengthens significantly. This is physically attributed to the increase in the moment of inertia associated with the heavier load. Crucially, across all tested conditions—whether varying pre-torsion turns or load masses—the strain components decoupled by our algorithm consistently demonstrate excellent agreement with experimental observations. These results further validate the robustness and reliability of our proposed method under dynamic loading.

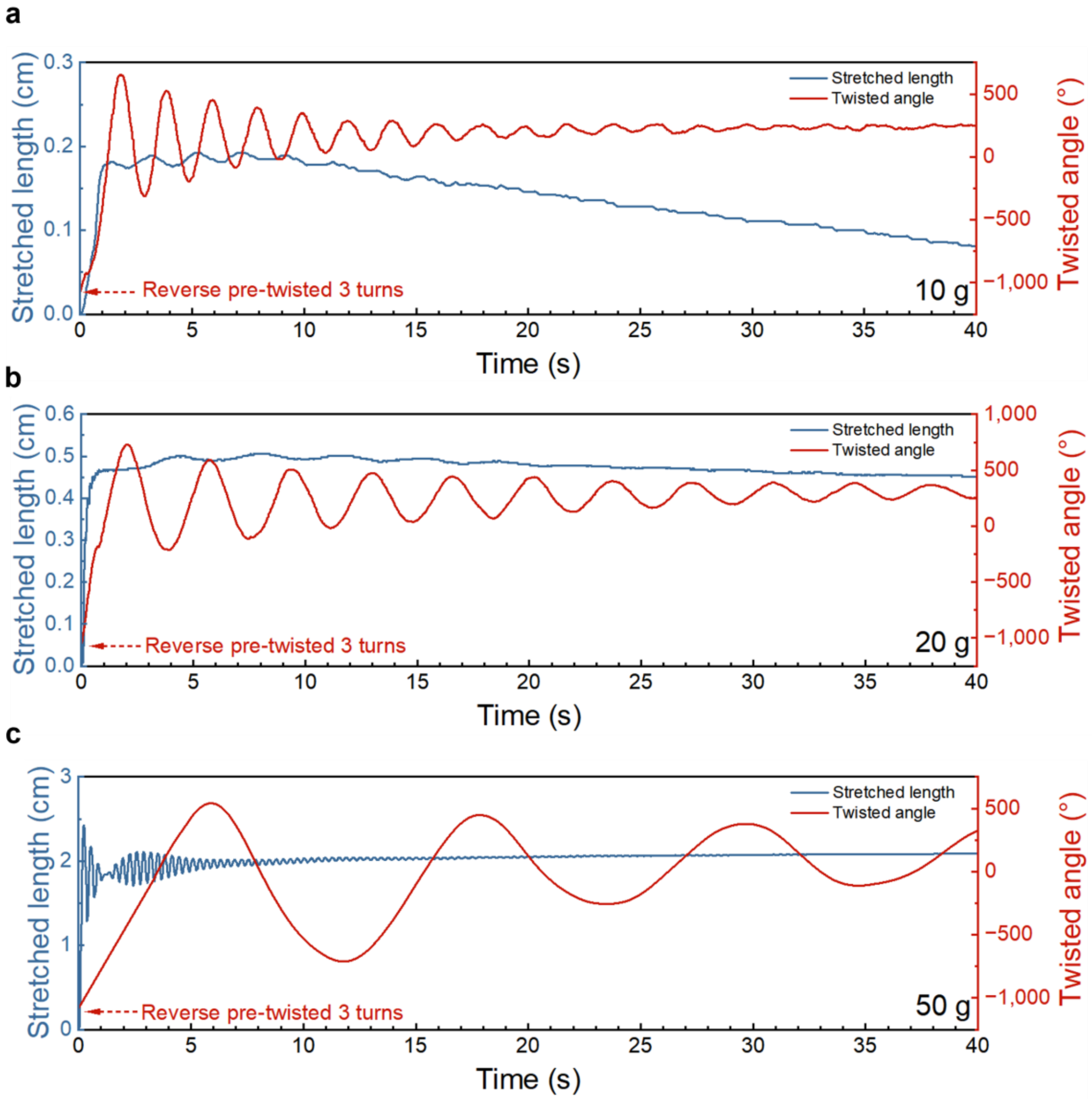

Supplementary Fig. 26 | Real-time decoupling performance under constant pre-twist turns and varying loads. a–c, Decoupled information under applied loads of 10 g **(a)**, 20 g **(b)**, and 50 g **(c)**

These additional data verify the generality and robustness of the proposed method within a reasonable range of pre-torsion levels. We have revised the relevant sections to clarify the rationale for the experimental conditions and parameter settings as follows:

[Manuscript, Page 21]

Furthermore, decoupling results obtained under varying load masses and pre-torsion turns are presented in Supplementary Figs. 25 and 26. These results highlight the sensor's

remarkable ability to monitor and decouple complex mechanical deformation states, providing valuable strategies and methodologies for applications demanding precise decoupling of composite motions.

[Supplementary Information, Page 43-44]

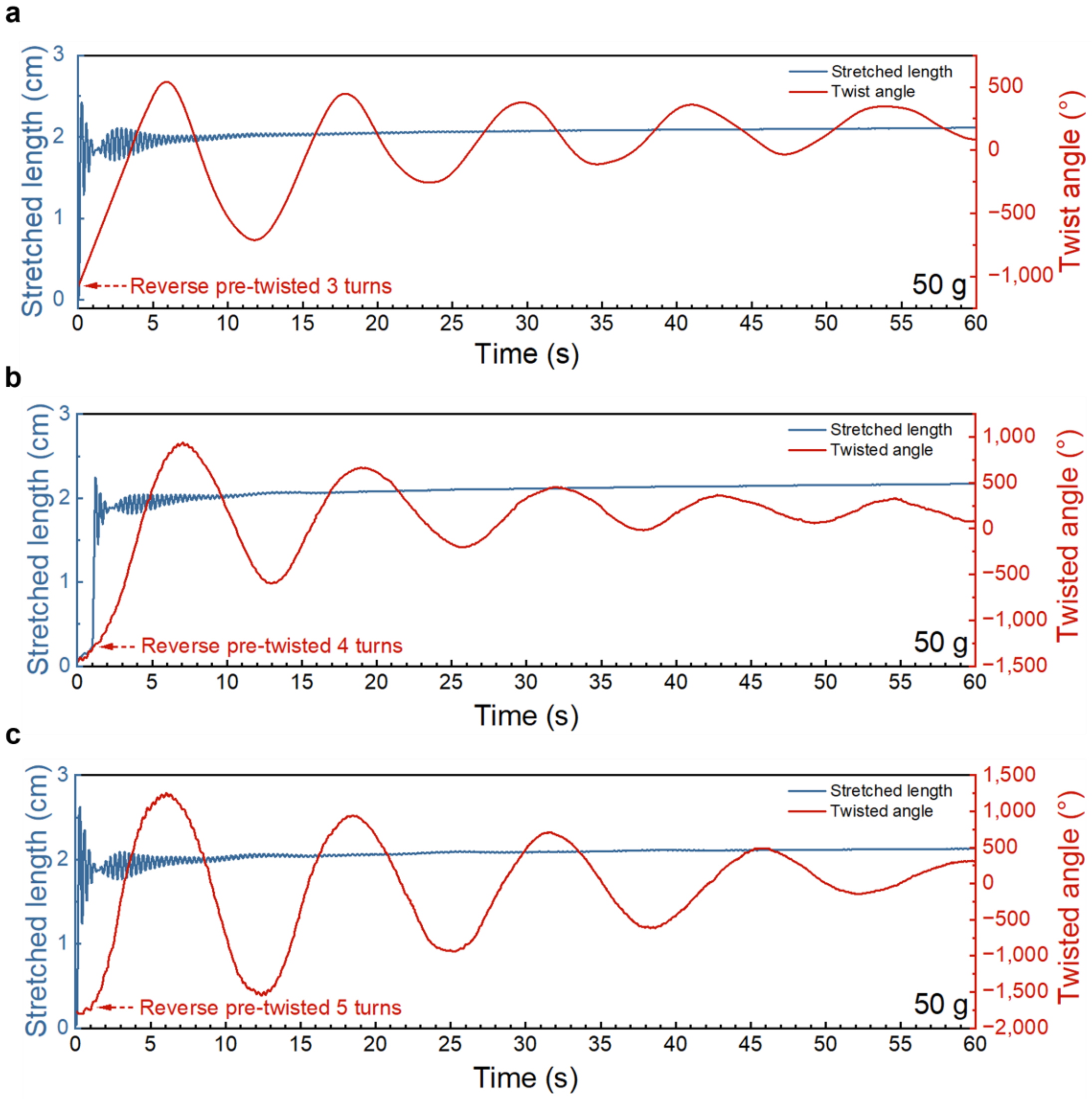

Supplementary Fig. 25 | Real-time decoupling performance under constant load and varying pre-twist turns. a–c, Decoupled information with pre-twist of 3 turns **(a)**, 4 turns **(b)**, and 5 turns **(c)**.

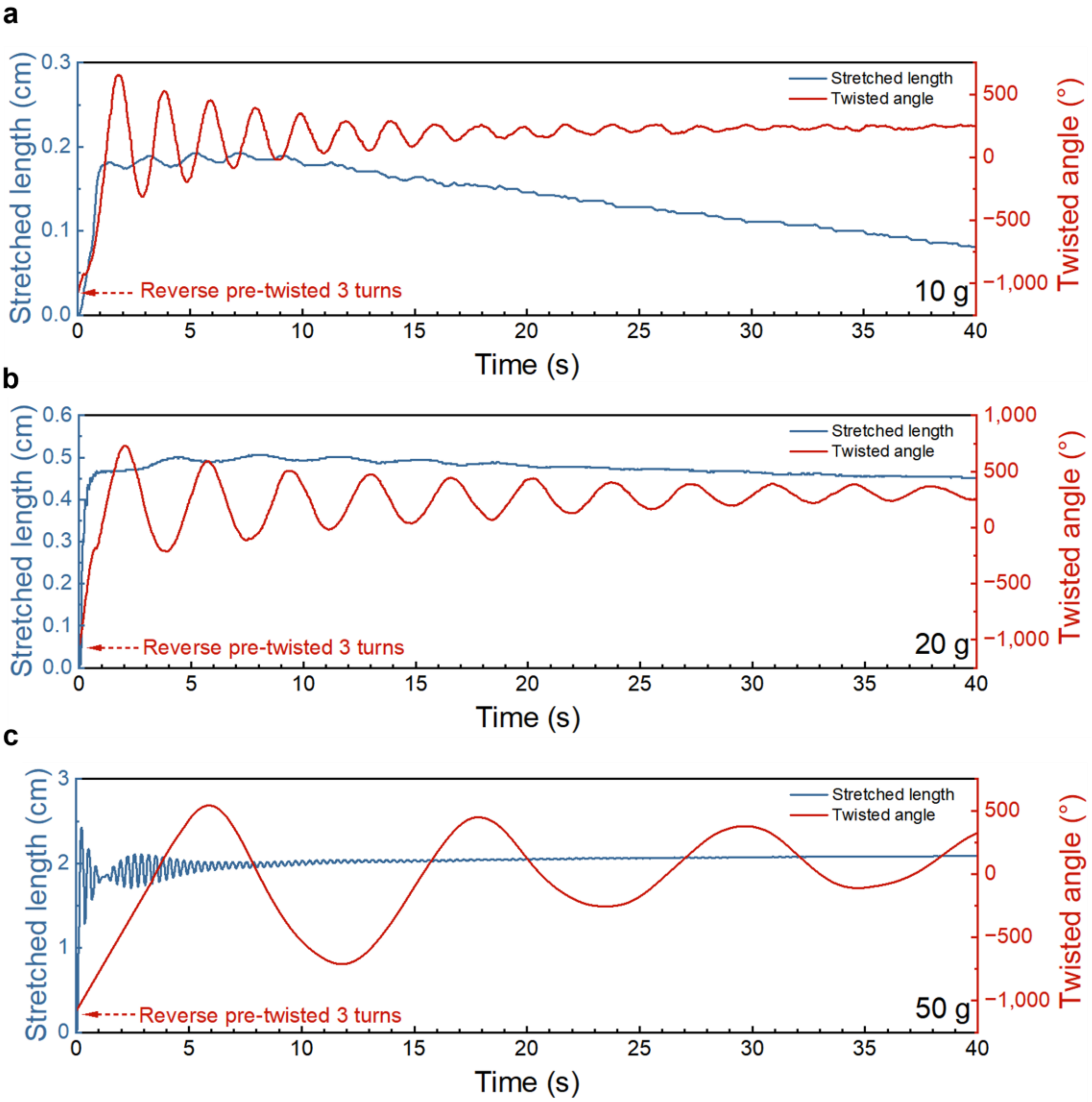

Supplementary Fig. 26 | Real-time decoupling performance under constant pre-twist turns and varying loads. a–c, Decoupled information under applied loads of 10 g **(a)**, 20 g **(b)**, and 50 g **(c)**

2. In responding to the question about the necessity of the three channels, if the authors emphasize that noise/coupling can undermine the stability of a simultaneous solution, they should also provide an error or robustness assessment for the sequential method (even a simple sensitivity analysis or repeatability statistics). Otherwise, the argument is not fully convincing.

Response: We sincerely appreciate the reviewer's valuable suggestion. We agree that a rigorous error and robustness assessment of the decoupling method is crucial to substantiate the necessity of the three-channel design. Accordingly, we have incorporated a comprehensive analysis into the revised manuscript to address this point, as detailed below:

(1) Calibration protocols and error analysis

Following established protocols for composite strain monitoring and decoupling, we systematically calibrated the fibre sensor prior to each test. Specifically, the calibration workflow proceeded as follows: First, we simultaneously acquired resistance and capacitance signals across a predefined set of composite strain combinations, as illustrated in Fig. 4f. Leveraging this experimental dataset in conjunction with the theoretical response model derived from the sensor's architecture, we performed non-linear fitting to optimise and calibrate the model parameters. This process yielded the robust numerical mapping model presented in Fig. 5a. This model enables the precise decoupling and reconstruction of individual strain component values from real-time electrical signals under unknown strain states. To evaluate the sensor's predictive accuracy and generalization capability, we validated it against a series of known composite strain conditions. **The comparison between the predicted values generated by the decoupling algorithm and the experimentally set ground truth values (Fig. 5b) reveals an exceptional degree of agreement across the entire testing range.** Furthermore, the repeated measurements at each strain combination and subsequent statistical error analysis, as summarized in Supplementary Fig. 24. Attributed to the highly consistent resistive response under tensile strain and its insensitivity to torsion (Supplementary Fig. 17c and d), the fibre sensor maintains a relative error below 1% for tensile strain measurements. Regarding the decoupling of torsional strain via capacitive signals, errors primarily stem from signal

fluctuations when torsion is superimposed onto tensile strain, yielding a relative error within 8%.

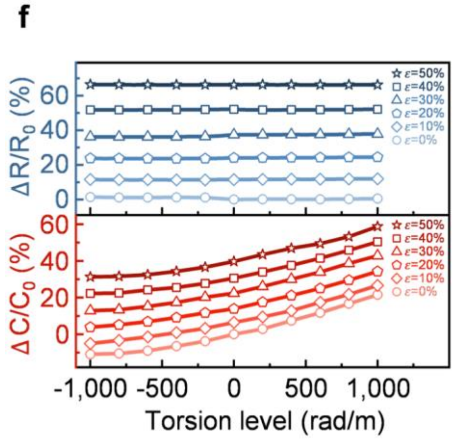

Fig. 4 | Electromechanical performance of dual-strain fibre sensor. f, Resistive and capacitive response of the sensor measured under torsional strain at various fixed tensile strains.

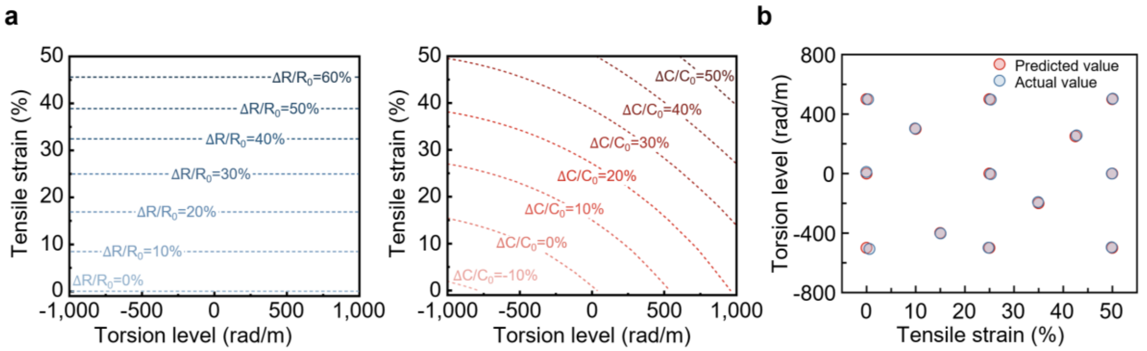

Fig. 5 | Composite motions decoupling performance of the dual-strain fibre sensor. a, Equivalent relationship curve between the influence of tensile strain and torsional strain on the capacitive and resistive response of the sensor. **b,** Prediction strain states of the sensor under different combinations of tensile strains and torsion levels.

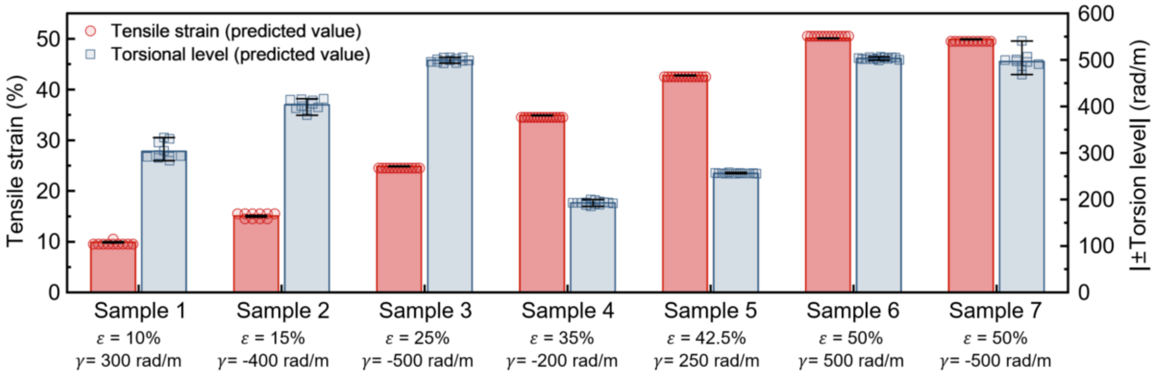

Supplementary Fig. 24 | Decoupling performance under composite strain combinations. Torsional strains are presented as absolute values. Error bars represent the standard deviation of the predicted values.

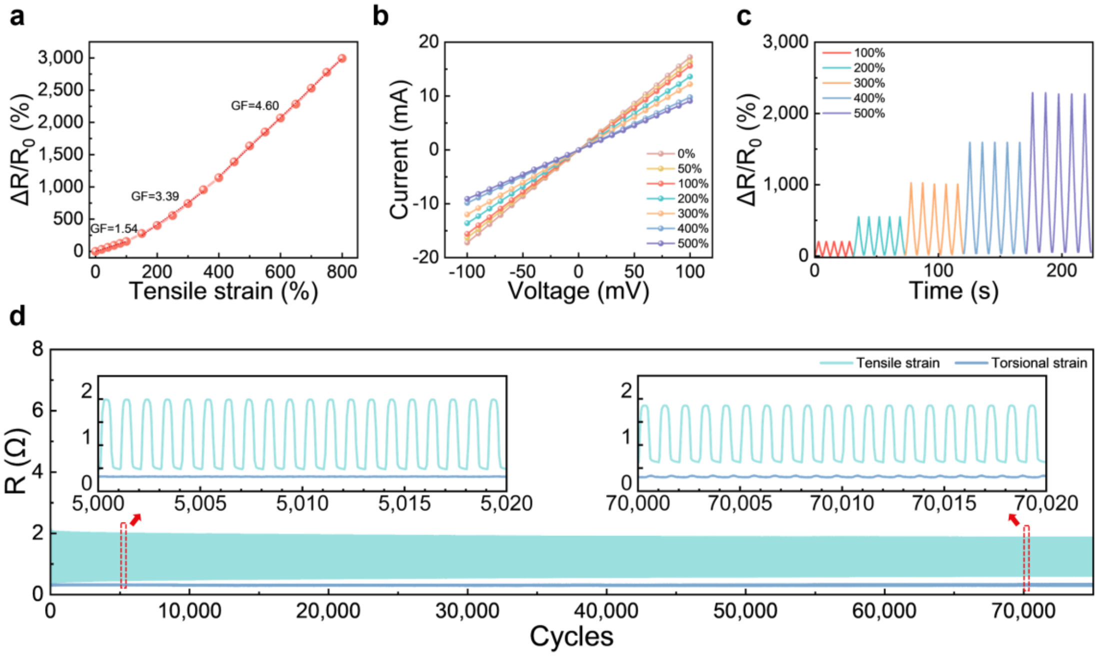

Supplementary Fig. 17 | Resistive electromechanical response characteristics of dual-strain fibre sensors. a, Resistive response of sensors under tensile strain. **b,** Volt-ampere characteristics curve of sensor under different tensile strains. **c,** Resistive response of the sensor under varying tensile strains (100–500%). **d,** Resistive response of the sensor under cyclic 200% tensile strain (cyan) and ±1,000 rad/m torsional strain (blue) over 75,000 cycles, respectively. The inset shows cycles from 5,000-5,020 and 70,000-70,020.

[Manuscript, Page 20]

The model's fidelity was validated under preset composite strain conditions to ensure reliability during dynamic monitoring. As shown in Fig. 5b and Supplementary Fig. 24, the system accurately predicts both strain states, with calculated values closely tracking the predefined parameters.

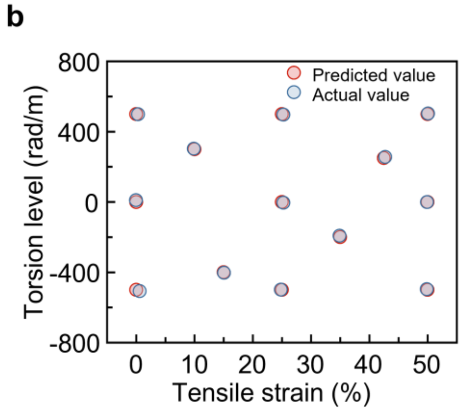

Fig. 5 | Composite motions decoupling performance of the dual-strain fibre sensor. b, Prediction strain states of the sensor under different combinations of tensile strains and torsion levels.

[Supplementary Information, Page 16]

Supplementary Note 6 | Real-time monitoring and computational decoupling of simultaneous tensile and torsional strains using the dual-strain fibre sensor.

The dual-strain decoupling algorithm proceeds in three sequential steps:

1. Calibration and modeling: The resistance and capacitance responses of the fibre sensor were measured under predetermined combinations of tensile and torsional strains (Fig. 4f). These datasets were used to calibrate the dual-strain sensor response, leading to a numerical model (Fig. 5a) established via nonlinear polynomial fitting and modeling analysis. The model was subsequently validated against preset composite strain conditions to ensure decoupling precision during real-time dynamic monitoring (Fig. 5b).

2. Tensile strain quantification: The tensile strain ε is first quantified directly from the relative resistance change $\frac{\Delta R}{R_0}$ using Equation (25), independent of torsion (Fig. 5a, left).

3. Torsional strain decoupling: The computed tensile strain ε is substituted into the Equation (27) alongside the measured relative capacitance variation $\frac{\Delta C}{C_0}$ to isolate and quantify the torsional strain (Fig. 5a, right).

Error analysis of composite strain sensing and decoupling: The sensor demonstrates a

relative tensile strain error of <1%, attributed to its highly consistent resistive response to tension and negligible cross-sensitivity to torsion. For torsional decoupling based on capacitive signals, the relative error is maintained within 8%, where deviations principally arise from signal fluctuations induced by superimposed tensile loading. To guarantee sustained fidelity during long-term dynamic monitoring, a periodic recalibration protocol at the zero-strain baseline is implemented to mitigate potential drift.

[Supplementary Information, Page 42]

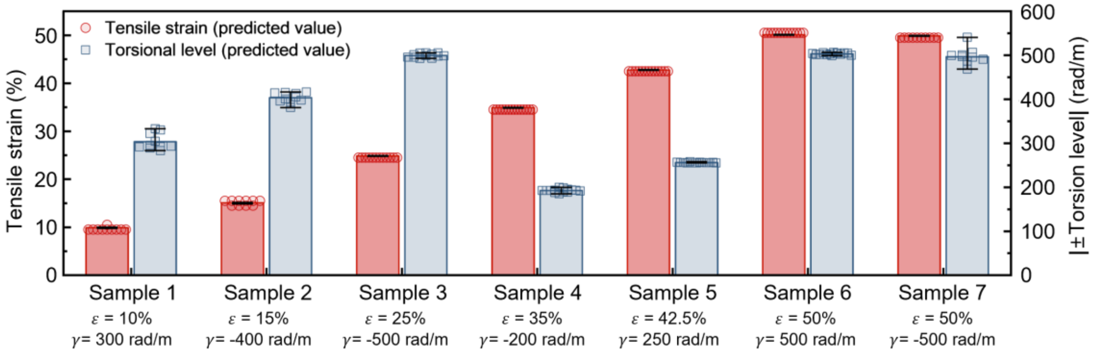

Supplementary Fig. 24 | Decoupling performance under composite strain combinations. Torsional strains are presented as absolute values. Error bars represent the standard deviation of the predicted values.

(2) Validation in dynamic scenarios

In the dynamic scenarios illustrated in Figs. 5c-e, the unconstrained motion of the released mass makes it challenging to establish "ground-truth" values for direct error calculation (as performed in static calibration). Consequently, we adopted a comparative strategy to validate the reliability of the decoupling method by benchmarking the digital twin reconstruction against experimental video analysis. As demonstrated in Supplementary Movie 1, the temporal profiles of the fibre sensor's axial length and twist angle, reconstructed via the decoupling algorithm, exhibit good agreement with the experimental video footage in terms of motion state, frequency, and amplitude. This

consistency substantiates the effectiveness and robustness of the decoupling algorithm in dynamic regimes.

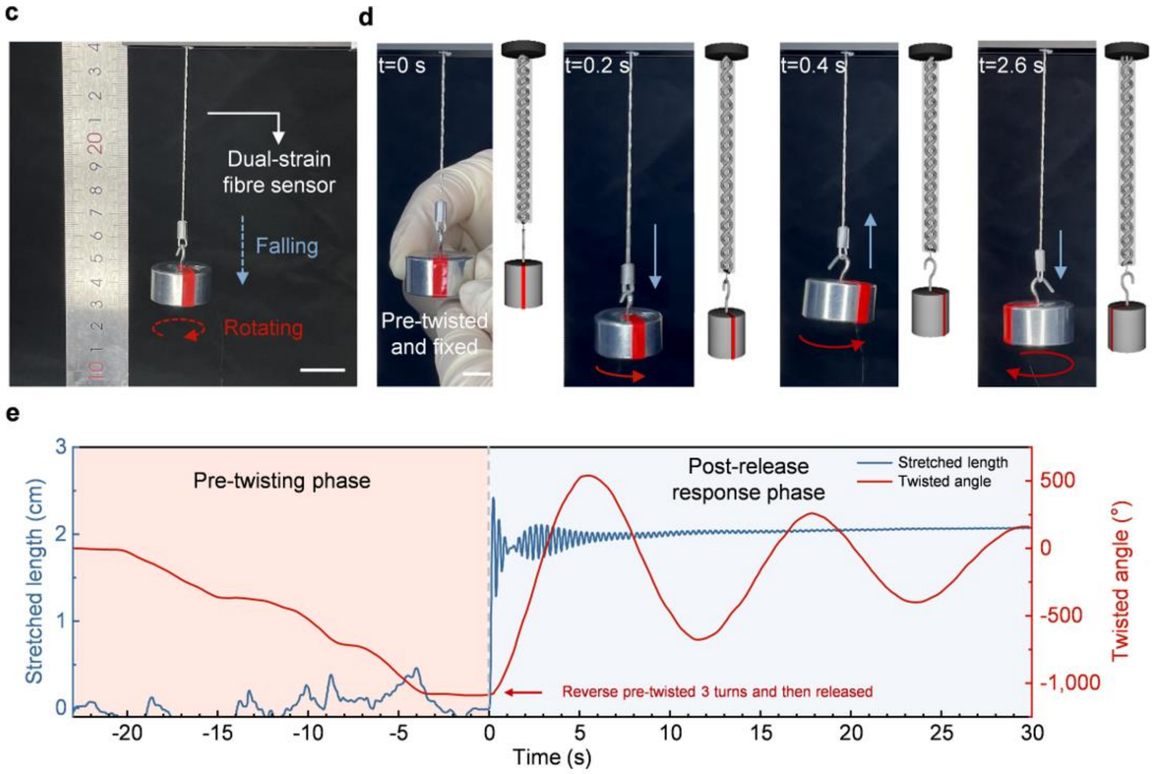

Fig. 5 | Composite motions decoupling performance of the dual-strain fibre sensor. c, Experimental platform for simultaneous monitoring and discrimination of tensile and torsional strains (scale bar, 2 cm). **d,** Digital twin model reflecting the real-time motion states of the objects (scale bar, 1 cm). **e,** Real-time response curves of the actual stretched length and twisted angle of the objects.

3. In response to the reviewer's questions on the model construction in Figure 5A and the identifiability of ε from ΔR/R0 under combined motion, the authors mainly restate the decoupling procedure without sufficient detail for reproducibility. Please (1) specify how the mathematical/numerical model is built, and (2) provide an error bound for ε estimation and a method to handle linear drift/offset. Otherwise, the current description is difficult to reproduce and verify independently.

Response: We appreciate the reviewer for this comment. We acknowledge that a clear elaboration of the mathematical model construction, quantitative error analysis of key

parameters are paramount for evaluating the rigor and reliability of this research. In response to the reviewer's suggestions, we have systematically expanded and clarified these aspects in both the revised main text and the Supplementary Information.

(1) Elaboration on the mathematical and numerical model construction

To balance main text readability with methodological completeness, the revised main text (Pages 13–15) now focuses on the physical principles of decoupling, leveraging the geometric structure of the three channels and their associated electrical signal variations. Comprehensive mathematical derivations, the model construction process, and parameter fitting details are detailed in **Supplementary Notes 4–6**. These sections derive the morphological changes of conductive microchannels within the fibre sensor under strains (Supplementary Fig. 16), and the analytical relationships of resistive and capacitive responses as functions of tensile (ε) and torsional (γ) strains, elucidating how experimental calibration data (Fig. 4f) are utilized to transform these theoretical relationships into a computational numerical model. The specific mathematical formulation of the fibre sensor response is detailed below:

As shown in Supplementary Fig. 16, when the fibre sensor is in its initial relaxed state, the radius of the helical microchannel is r_0, the distance between the centers of the two helical channels is d_0, and the helical period is h_0.

- **Responses under pure tensile strain** (detailed in Supplementary Note 4):

$$\frac{\Delta R}{R_0}(\varepsilon) = (\varepsilon + 1)^2 - 1$$

$$\frac{\Delta C}{C_0}(\varepsilon) = (1+\varepsilon)\sqrt{\frac{(1+\varepsilon)^3 + \left(\frac{\pi d_0}{h_0}\right)^2}{(1+\varepsilon)^3\left(1+\left(\frac{\pi d_0}{h_0}\right)^2\right)}} \times \frac{\ln\left(\frac{d_0}{2r_0} + \sqrt{\left(\frac{d_0}{2r_0}\right)^2 - 1}\right)}{\ln\left\{\frac{d_0}{2r_0}\left(\frac{(1+\varepsilon)^3 + \left(\frac{\pi d_0}{h_0}\right)^2}{(1+\varepsilon)^3\left(1+\left(\frac{\pi d_0}{h_0}\right)^2\right)}\right)^{\frac{1}{4}} + \sqrt{\left(\frac{d_0}{2r_0}\right)^2\left(\frac{(1+\varepsilon)^3 + \left(\frac{\pi d_0}{h_0}\right)^2}{(1+\varepsilon)^3\left(1+\left(\frac{\pi d_0}{h_0}\right)^2\right)}\right)^{\frac{1}{2}} - 1}\right\}} - 1$$

- **Responses under pure torsional strain** (detailed in Supplementary Note 5):

The resistive response is insensitive to torsional strain.

$$\frac{\Delta R}{R_0}(\gamma) = 0$$

$$\frac{\Delta C}{C_0}(\gamma) = \sqrt{\frac{1+\left(\frac{\pi d_0}{h_0}+\frac{\gamma d_0}{2}\right)^2}{1+\left(\frac{\pi d_0}{h_0}\right)^2}} \times \frac{\ln\left(\frac{d_0}{2r_0}+\sqrt{\left(\frac{d_0}{2r_0}\right)^2-1}\right)}{\ln\left(\frac{d_0}{2r_0}\left(\frac{1+\left(\frac{\pi d_0}{h_0}+\frac{\gamma d_0}{2}\right)^2}{1+\left(\frac{\pi d_0}{h_0}\right)^2}\right)^{\frac{1}{4}}+\sqrt{\left(\frac{d_0}{2r_0}\right)^2\left(\frac{1+\left(\frac{\pi d_0}{h_0}+\frac{\gamma d_0}{2}\right)^2}{1+\left(\frac{\pi d_0}{h_0}\right)^2}\right)^{\frac{1}{2}}-1}\right)} - 1$$

- **Responses under composite tensile and torsional strains** (detailed in Supplementary Note 6):

$$\frac{\Delta R}{R_0}(\varepsilon,\gamma) = (\varepsilon+1)^2 - 1$$

$$\frac{\Delta C}{C_0}(\varepsilon,\gamma) = \sqrt{\frac{(1+\varepsilon)^3+\left[\frac{\pi d_0}{h_0}+(1+\varepsilon)\frac{\gamma d_0}{2}\right]^2}{(1+\varepsilon)\left[1+\left(\frac{\pi d_0}{h_0}\right)^2\right]}} \frac{\ln\left(\frac{d_0}{2r_0}+\sqrt{\left(\frac{d_0}{2r_0}\right)^2-1}\right)}{\ln\left\{\frac{d_0}{2r_0}\left\{\frac{(1+\varepsilon)^3+\left[\frac{\pi d_0}{h_0}+(1+\varepsilon)\frac{\gamma d_0}{2}\right]^2}{(1+\varepsilon)^3\left[1+\left(\frac{\pi d_0}{h_0}\right)^2\right]}\right\}^{\frac{1}{4}}+\sqrt{\left(\frac{d_0}{2r_0}\right)^2\left\{\frac{(1+\varepsilon)^3+\left[\frac{\pi d_0}{h_0}+(1+\varepsilon)\frac{\gamma d_0}{2}\right]^2}{(1+\varepsilon)^3\left[1+\left(\frac{\pi d_0}{h_0}\right)^2\right]}\right\}^{\frac{1}{2}}-1}\right\}} - 1$$

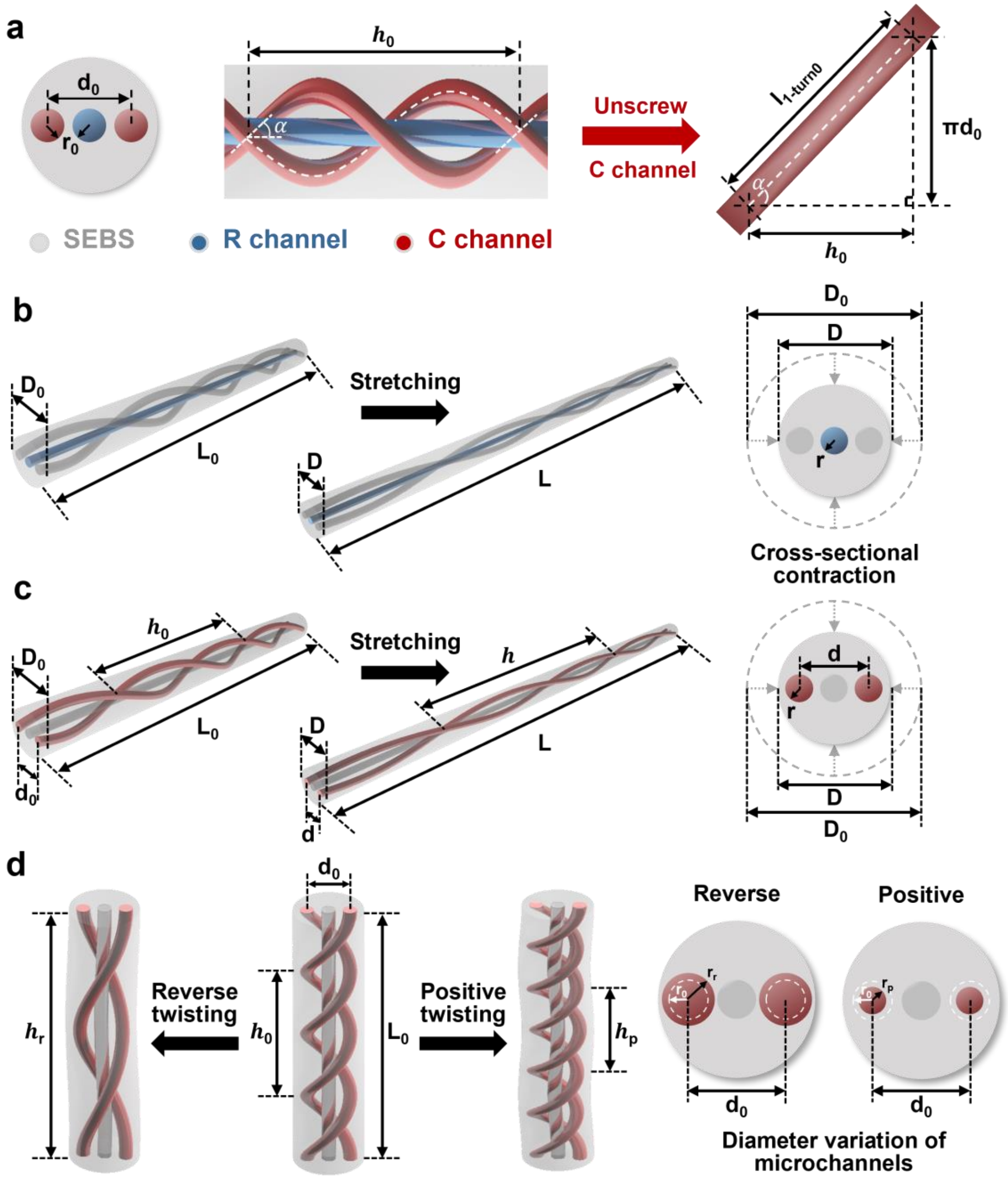

Supplementary Fig. 16 | Working mechanism of the dual-strain fibre sensor for tensile and torsional strain sensing. a, The cross-section and side diagrams of the fibre strain sensor. The white dashed in the helical capacitance channel within the fibre highlights a single period of the helical channel and its unscrew version. **b,** Schematic diagram of resistance channel variation under tensile strain. **c,** Schematic diagram of capacitance channel variation under tensile strain. **d,** Schematic diagram of capacitance channel under torsional strain.

Accordingly, we have revised the relevant sections to further clarify the sensing mechanism and formulation of the mathematical model.

[Manuscript, Page 13]

The $\Delta R/R_0$ exhibits a quadratic dependence on tensile strain, whereas the $\Delta C/C_0$ displays a linear relationship (see Supplementary Note 4 for details).

[Manuscript, Page 14]

Furthermore, the effects of positive and reverse torsion on the helical microchannels structure are oppositional, enabling the determination of torsion direction based on the positive and negative relative changes in capacitive signal (see Supplementary Note 5 for details).

[Manuscript, Page 14]

When tensile and torsional strains are applied concurrently, the resistive signal responds exclusively to tensile strain while remaining insensitive to torsion. Conversely, variations in the capacitive signal arise from the synergistic influence of both strain types. Consequently, decoupling these combined loading conditions is unachievable by monitoring either the resistive or capacitive signal in isolation. This necessitates a system-level integration strategy, wherein multiple microchannels are strategically utilized to incorporate distinct sensing mechanisms within a single fibre, thereby enabling strain separation through analytical modeling. Specifically, this involves a sequential analytical approach: first quantifying tensile strain via the resistive signal, and subsequently utilizing this value alongside the capacitive response to decouple and resolve the torsional strain (see Supplementary Note 6 for details). Thus, the synergistic utilization of both electrical sensing mechanisms is essential to not only distinguish individual strains, but also effectively decouple and monitor them under complex combined loading conditions.

(2) Error analysis of tensile strain and drift mitigation

Quantifying the error of key parameters is critical for assessing method robustness. Benefiting from the high consistency of the resistive signal under tensile strain and its insensitivity to torsion, the fibre sensor maintains a relative error below 1% in tensile strain measurements (Supplementary Fig. 24). Based on calibration measurements and error analysis, we have quantified the theoretical uncertainty in deriving tensile strain ε from $\Delta R/R_0$ and established corresponding error bounds within the sensor's operational range for combined tensile and torsional strains (Fig. 5b). Furthermore, our decoupling algorithm relies on relative changes ($\Delta R/R_0$, $\Delta C/C_0$) from calibrated baselines (R_0, C_0) rather than absolute electrical values. This relative-measurement approach effectively mitigates the impact of slow linear drift, Concurrently, systematic testing of signal stability under long-term cyclic loading reveals that the sensor maintains exceptional stability with negligible drift over hundred thousand cycles of tensile and torsional deformation (Supplementary Fig. 11). For dynamic or long-term monitoring, we implement pre-use re-calibration and periodic zero-strain checks to ensure sustained sensing and decoupling accuracy. In addition, to facilitate independent reproduction of this work, we have provided a comprehensive, step-by-step description of the calibration and measurement procedures in Supplementary Note 6. Relevant updates have been incorporated into the revised manuscript.

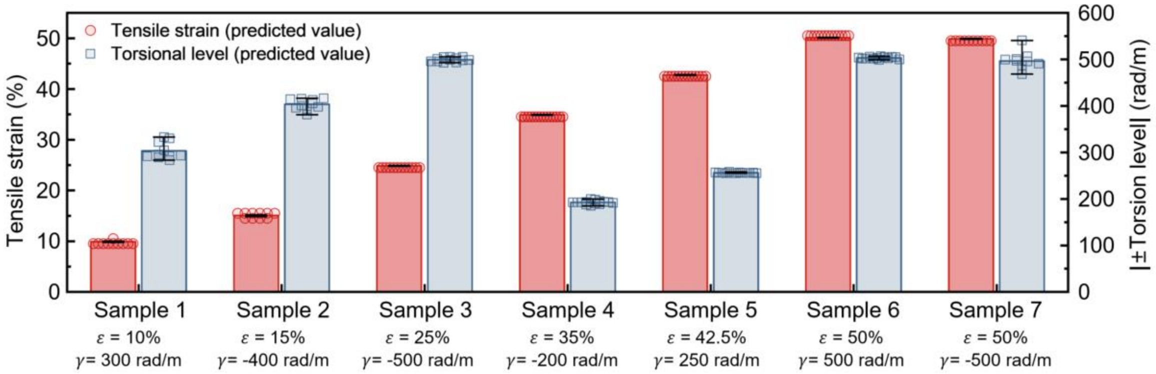

Supplementary Fig. 24 | Decoupling performance under composite strain combinations. Torsional strains are presented as absolute values. Error bars represent the standard deviation of the predicted values.

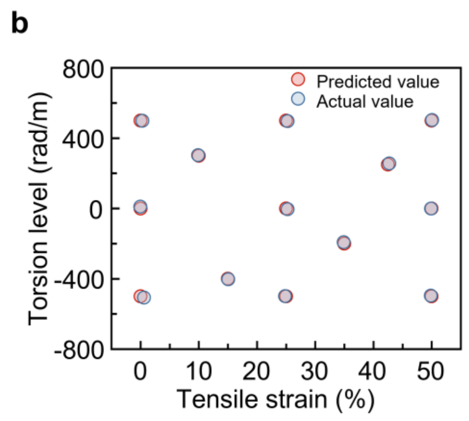

Fig. 5 | Composite motions decoupling performance of the dual-strain fibre sensor. b, Prediction strain states of the sensor under different combinations of tensile strains and torsion levels.

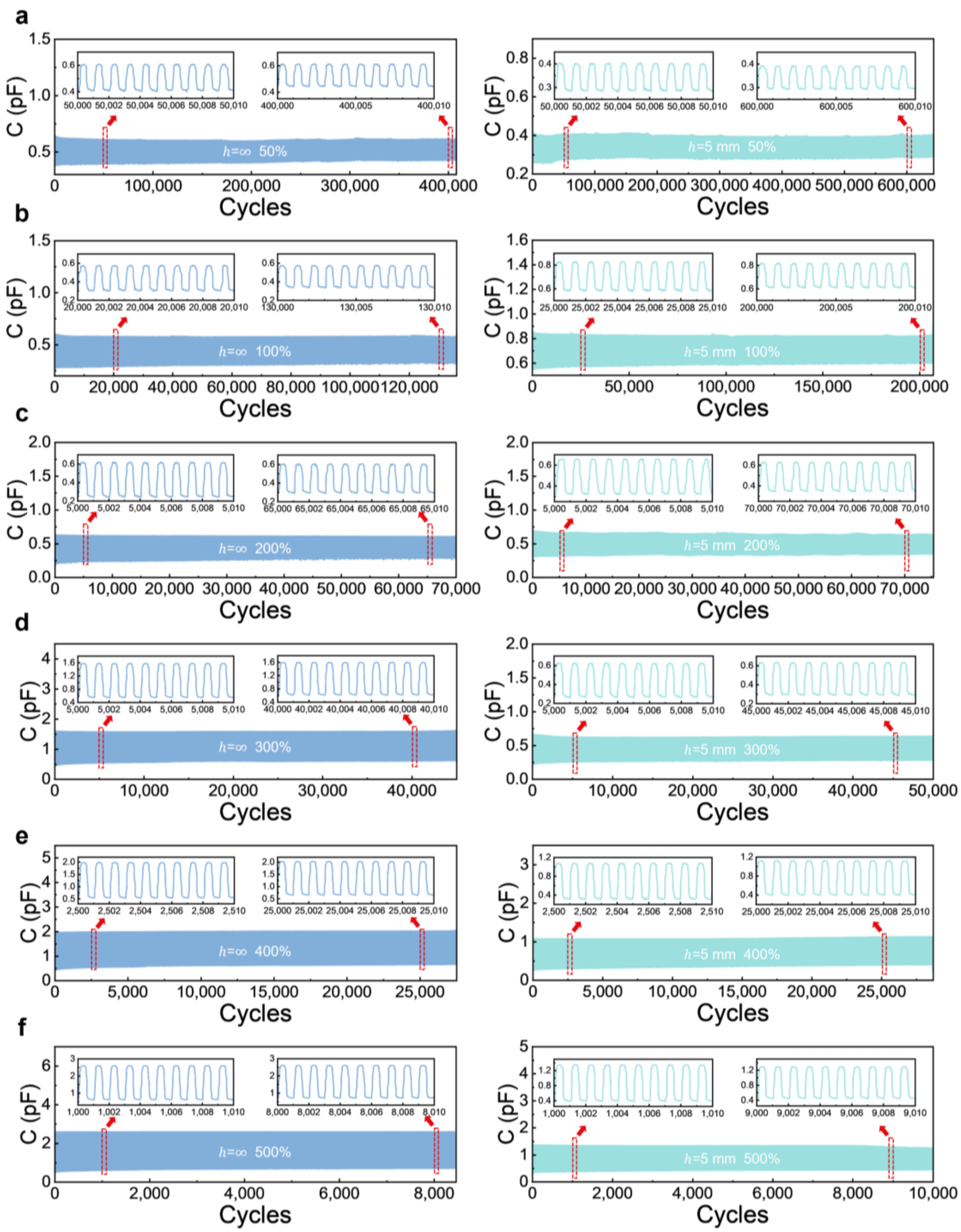

Supplementary Fig. 11 | Cyclic tensile durability of fibres with straight (*h*=∞, blue) versus helical (*h*=5 mm, cyan) microchannels. a, Under 50% cyclic tensile strain, maximum durability cycles reach 408,500 (*h*=∞) and 641,400 (*h*=5 mm). **b,** Under 100% cyclic tensile strain, maximum durability cycles reach 135,500 (*h*=∞) and 206,600 (*h*=5 mm). **c,** Under 200% cyclic tensile strain, maximum durability cycles reach 70,200 (*h*=∞) and 75,500 (*h*=5 mm). **d,** Under 300% cyclic tensile strain, maximum durability cycles reach 45,300 (*h*=∞) and 50,500 (*h*=5 mm). **e,** Under 400%

cyclic tensile strain, maximum durability cycles reach 27,600 (h=∞) and 28,600 (h=5 mm). **f,** Under 500% cyclic tensile strain, maximum durability cycles reach 8,400 (h=∞) and 10,100 (h=5 mm).

[Supplementary Information, Page 16]

Supplementary Note 6 | Real-time monitoring and computational decoupling of simultaneous tensile and torsional strains using the dual-strain fibre sensor.

Error analysis of composite strain sensing and decoupling: The sensor demonstrates a relative tensile strain error of <1%, attributed to its highly consistent resistive response to tension and negligible cross-sensitivity to torsion. For torsional decoupling based on capacitive signals, the relative error is maintained within 8%, where deviations principally arise from signal fluctuations induced by superimposed tensile loading. This discrepancy is primarily ascribed to the dynamic redistribution of liquid metal within the helical microchannels and interfacial phenomena occurring during deformation. To guarantee sustained fidelity during long-term dynamic monitoring, a periodic recalibration protocol at the zero-strain baseline is implemented to mitigate potential drift.

4. In the final experiment using a 50 g weight, the authors state that "tests were conducted with weights of different masses, and the 50 g weight is presented as a representative case." It is recommended that the authors summarize the tested load conditions in a table, or at least provide 2–3 additional representative cases, to support the claim that the results do not rely on a specific load. The claim that the response is "largely independent of load parameters" may be inaccurate. Load mass/geometry changes inertia, which can modify the coupled tensile–torsional dynamics and thus the strain waveforms in dynamic conditions. Please discuss this dependence and/or provide comparative tests with different masses/shapes to demonstrate the robustness of the decoupling algorithm under dynamic loading.

Response: We thank the reviewer for this comment. We acknowledge that the load mass and geometry can influence the coupled tensile-torsional dynamics in our real-time experiment, and that demonstrating the decoupling algorithm's robustness across varied loading conditions is essential. We concur that our original presentation using a single 50 g weight was insufficient to support a broad claim of load independence.

While the 50 g weight served as a representative example, we have now expanded our validation as shown in Supplementary Figs. 25 and 26. These figures present decoupling results under (i) varying pre-torsion turns with constant load mass, and (ii) varying load masses with constant pre-torsion turns, thereby demonstrating the generalizability of our approach.

Experimental observations indicate that increasing the pre-torsion turns under a constant load mass predominantly influences the system's torsional response (Supplementary Fig. 25). Specifically, the initial amplitude of torsional oscillation increases significantly, whereas the oscillation period remains largely unaffected. In contrast, the impact on tensile oscillation is marginal, with the tensile strain continuing to oscillate within a comparable range. This behaviour is attributed to the fact that pre-torsion primarily alters the system's initial torsional potential energy while having a negligible impact on axial stiffness within an appropriate torsional range, thereby preserving the stability of the tensile response dynamics.

Conversely, under a constant pre-torsion angle, varying the load mass significantly alters the dynamic response characteristics for both tension and torsion (Supplementary Fig. 26). As the load mass increases, the tensile equilibrium length extends, and the total duration of oscillation increases. Notably, under low-mass conditions (e.g., 10 g), the system exhibits slow elastic recovery, characterized by a gradual decrease in equilibrium length during oscillation (Supplementary Fig. 26a). This phenomenon arises because the reduced inertial forces associated with lower mass render damping effects and the fibre's

viscoelastic relaxation behaviour more pronounced in the macroscopic response. Regarding torsional oscillation, while the initial amplitude remains substantially constant as mass increases, the oscillation period lengthens significantly. This is attributed to the increased moment of inertia associated with the heavier load. Crucially, across all tested conditions—whether varying pre-torsion turns or load masses—the strain components decoupled by our algorithm consistently demonstrate excellent agreement with experimental observations. These results further validate the robustness and reliability of our proposed method under dynamic loading.

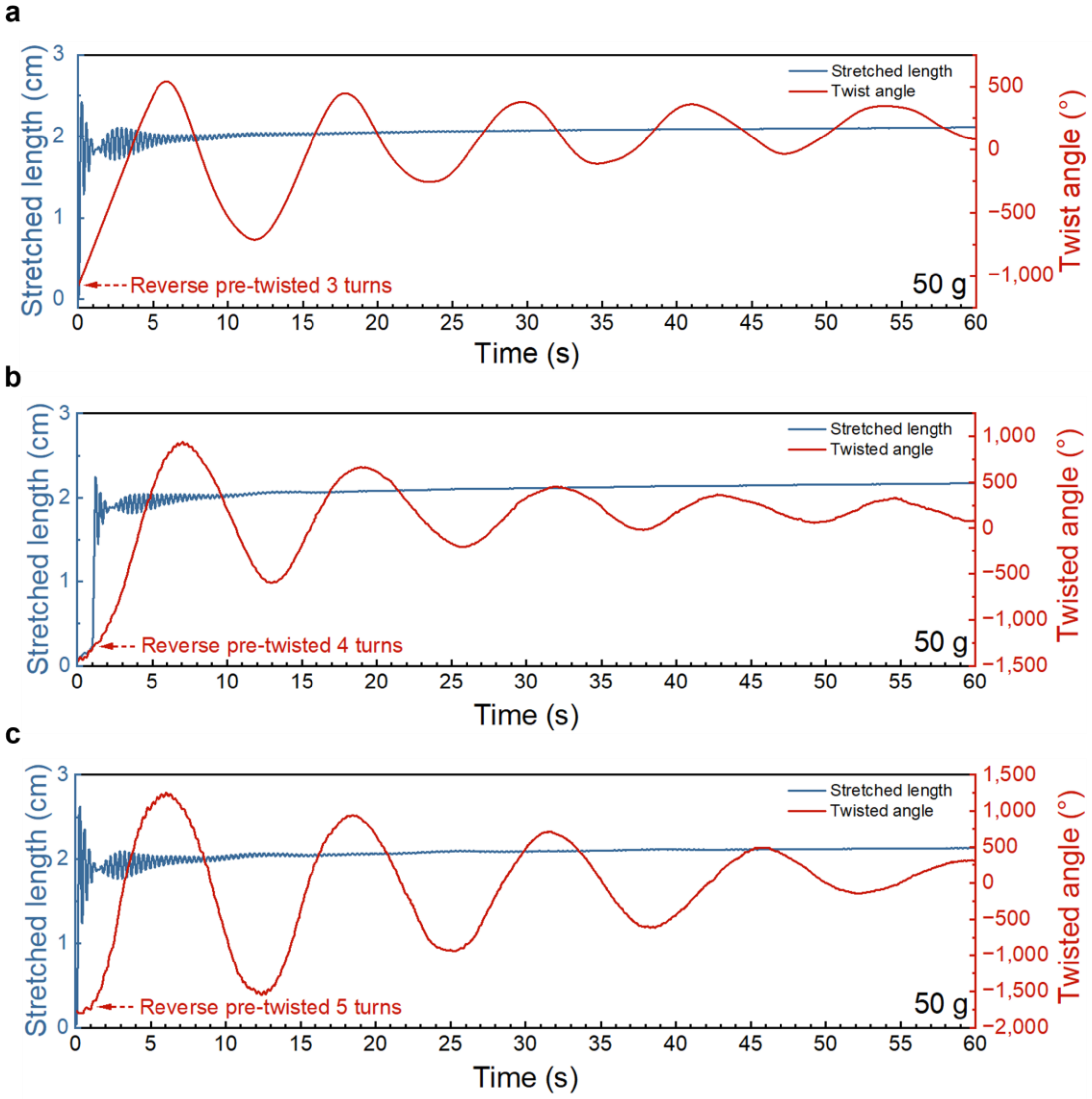

Supplementary Fig. 25 | Real-time decoupling performance under constant load and varying pre-twist turns. a–c, Decoupled information with pre-twist of 3 turns **(a)**, 4 turns **(b)**, and 5 turns **(c)**.

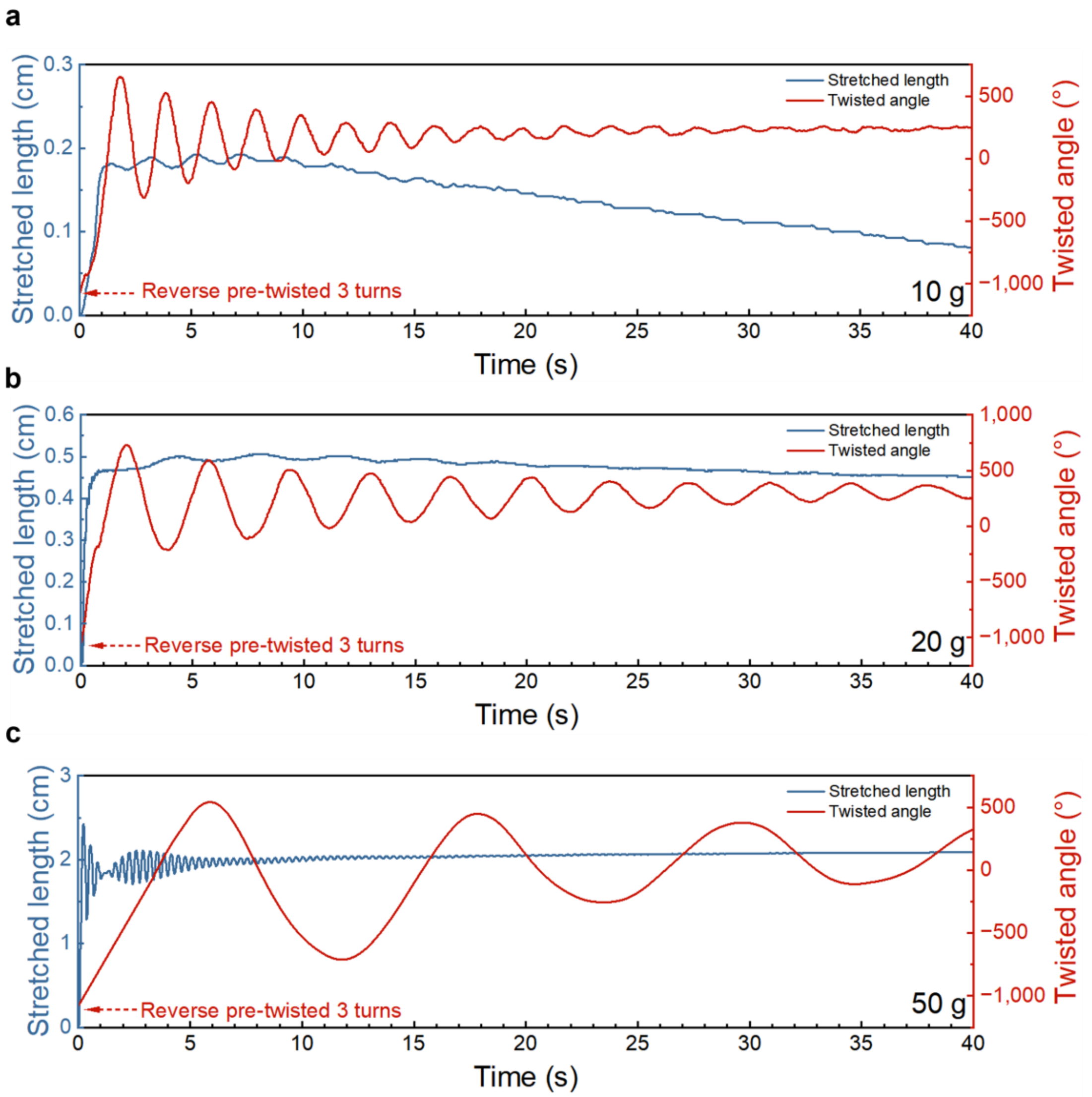

Supplementary Fig. 26 | Real-time decoupling performance under constant pre-twist turns and varying loads. a–c, Decoupled information under applied loads of 10 g **(a)**, 20 g **(b)**, and 50 g **(c)**

These additional data confirm the generality and robustness of the proposed method within a reasonable range of pre-torsion levels. We have revised the relevant sections to clarify the rationale for the experimental conditions and parameter settings as follows:

[Manuscript, Page 21]

Furthermore, decoupling results obtained under varying load masses and pre-torsion turns are presented in Supplementary Figs. 25 and 26. These results highlight the sensor's remarkable ability to monitor and decouple complex mechanical deformation states, providing valuable strategies and methodologies for applications demanding precise decoupling of composite motions.

[Supplementary Information, Page 43-44]

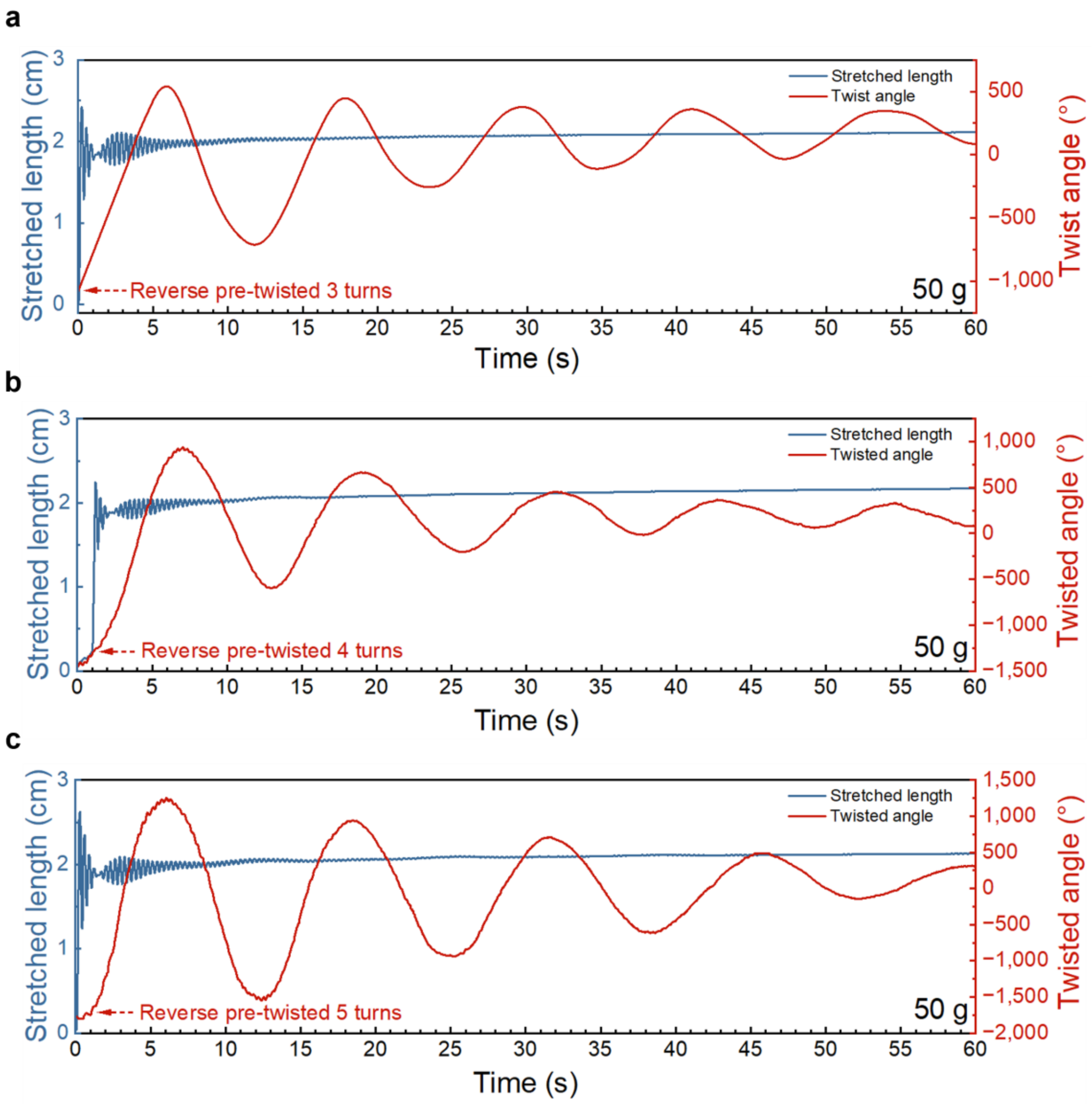

Supplementary Fig. 25 | Real-time decoupling performance under constant load and varying pre-twist turns. a–c, Decoupled information with pre-twist of 3 turns **(a)**, 4 turns **(b)**, and 5 turns **(c)**.

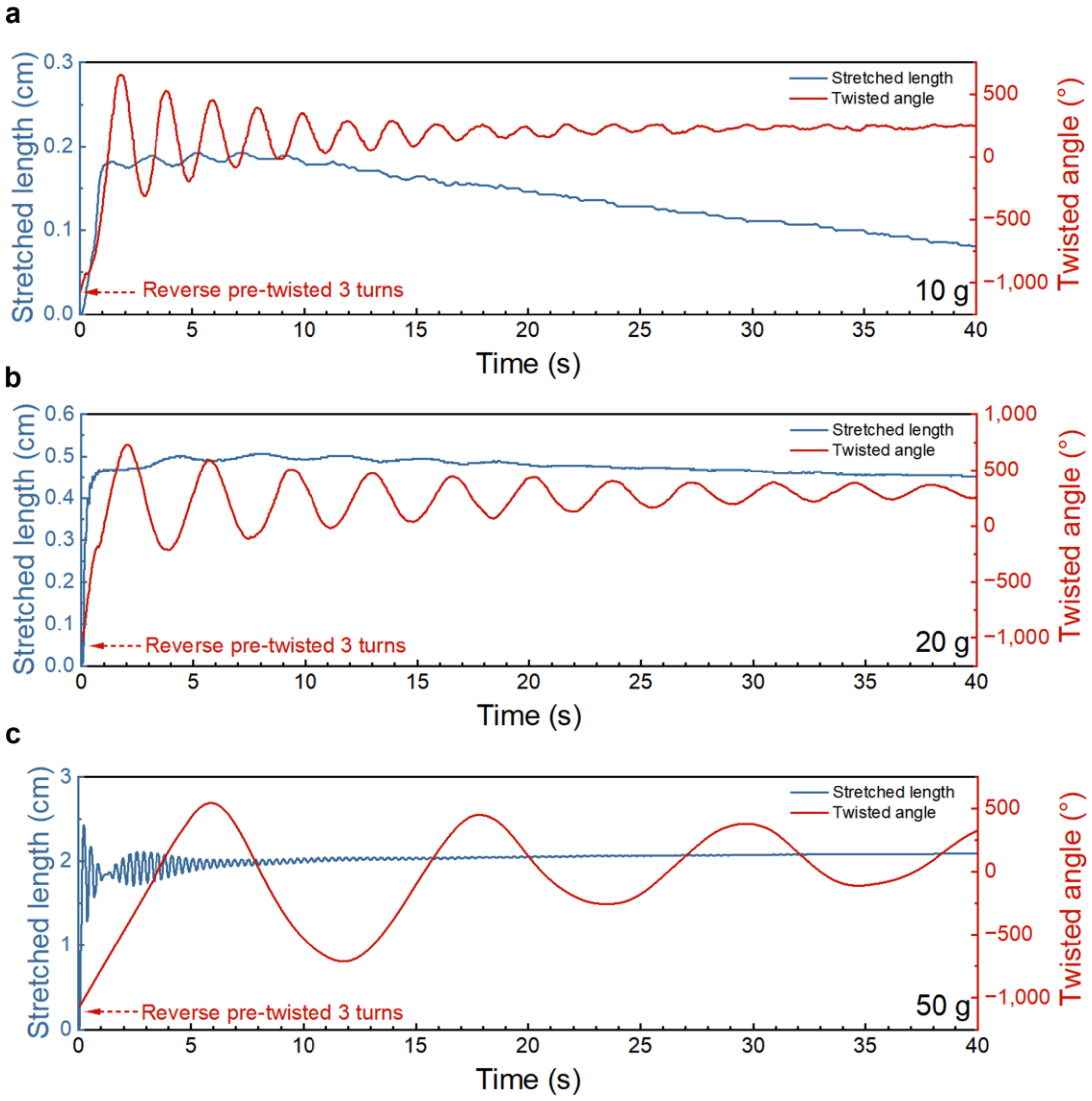

Supplementary Fig. 26 | Real-time decoupling performance under constant pre-twist turns and varying loads. a–c, Decoupled information under applied loads of 10 g **(a)**, 20 g **(b)**, and 50 g **(c)**

Response to Reviewer #3:

This manuscript presents a high-quality and well-executed engineering study with thorough characterization and convincing performance demonstrations. However, the central claims of novelty—particularly concerning rotational thermal stretching and dual-strain decoupling—require stronger mechanistic substantiation to rise to the level of conceptual innovation expected for publication in this venue.

Response: We thank the reviewer for the positive assessment of our study's technical quality. To address the concerns regarding the novelty of our work, we have revised our manuscript and Supplementary Information to clearly demonstrate the mechanism by which our highly stretchable fibre achieves the decoupling of composite motions.

First, regarding the mechanistic innovation in regulating material mechanical properties: By integrating theoretical analysis with experimental characterisation, **we have elucidated—for the first time—the mechanism by which rotational thermal drawing modulates polymer chain dynamics.** Our results reveal that the rotational torque applied during drawing induces a helical reorientation of polymer chains along the fibre axis. This induced structural anisotropy yields a synergistic enhancement in mechanical performance, notably increasing the elongation at break from 805% to 1,918%. This discovery establishes a foundational paradigm for mechanically reinforcing soft electronics through precise microstructural engineering.

Second, regarding the advancement in device performance: We have engineered a dual-strain fibre sensor that effectively overcomes existing trade-offs in multimodal sensing, exhibiting benchmark performance metrics: (i) highly linear response to tensile strain, with $R^2 > 0.999$ over a broad range of 0-800%; (ii) wide bidirectional torsion sensing range of ±2000 rad/m; and (iii) superior durability exceeding 75,000 cycles at 200% tensile strain and 425,000 at ±1,000 rad/m torsional strain. Comparative analysis

confirms that our sensor outperforms state-of-the-art multimodal counterparts across these critical metrics, setting a new standard for durability and linearity.

Third, regarding systemic and methodological innovation: We present a pioneering approach for the accurate, real-time decoupling and monitoring of composite tensile-torsional deformations using our dual-strain fibre sensor. This is achieved through a tri-channel fibre that integrates concentric straight and double-helical microchannel architectures. A dedicated algorithm leverages the differential electrical responses of these distinct microchannels under tensile and torsional loading to enable the real-time decoupling and concurrent measurement of both strain components. This strategy effectively overcomes a key limitation of conventional strain sensors, which typically struggle to decouple multiple strain components under complex loading conditions. Critically, this work provides a scalable and robust paradigm for multidimensional deformation sensing, paving the way for next-generation wearable electronics capable of operating in complex, real-world environments.

We have meticulously addressed the reviewer's comments and incorporated corresponding revisions to strengthen the mechanistic substantiation of our work. We trust that these enhanced explanations and additional data satisfactorily clarify the conceptual novelty and scientific rigor of this study.

1. The claimed novelty of rotational thermal stretching in enhancing mechanical properties is not convincingly demonstrated at the mechanistic level. Thermal drawing is already known to improve fiber mechanics by inducing polymer chain orientation. To establish true novelty, the authors would need to show that rotation leads to a unique structural or molecular outcome beyond conventional hot drawing (e.g., via direct comparison against non-rotational drawing or quantitative

orientation analysis). At present, the manuscript mainly reports structural changes and improved performance, rather than a distinct mechanism attributable to rotation.

Response: We appreciate the reviewer for this insightful comment. We agree that a deeper mechanistic explanation is essential to elucidate the specific role of rotational thermal drawing. **In this work, rotation during thermal drawing serves not merely as an auxiliary step, but as a critical strategy to induce a three-dimensional helical orientation of polymer chains, which is fundamentally distinct from the uniaxial alignment typical of conventional non-rotational drawing.**

To substantiate this, we have revised the relevant sections and presented direct evidence in Fig. 2 and Supplementary Notes 2-3:

(1) Quantitative chain orientation analysis: SAXS measurements and analysis (Fig. 2b-d) confirm that rotation during thermal drawing induces a helical alignment of SEBS chains, characterized by a distinct tilt angle (δ) relative to the fibre axis. In contrast, fibres drawn without rotation ($h = \infty$) exhibit purely axial alignment ($\delta \approx 0$). This helical chain configuration functions as a series of molecular "springs," thereby enhancing energy dissipation and impeding fracture propagation, as detailed in Supplementary Note 3.

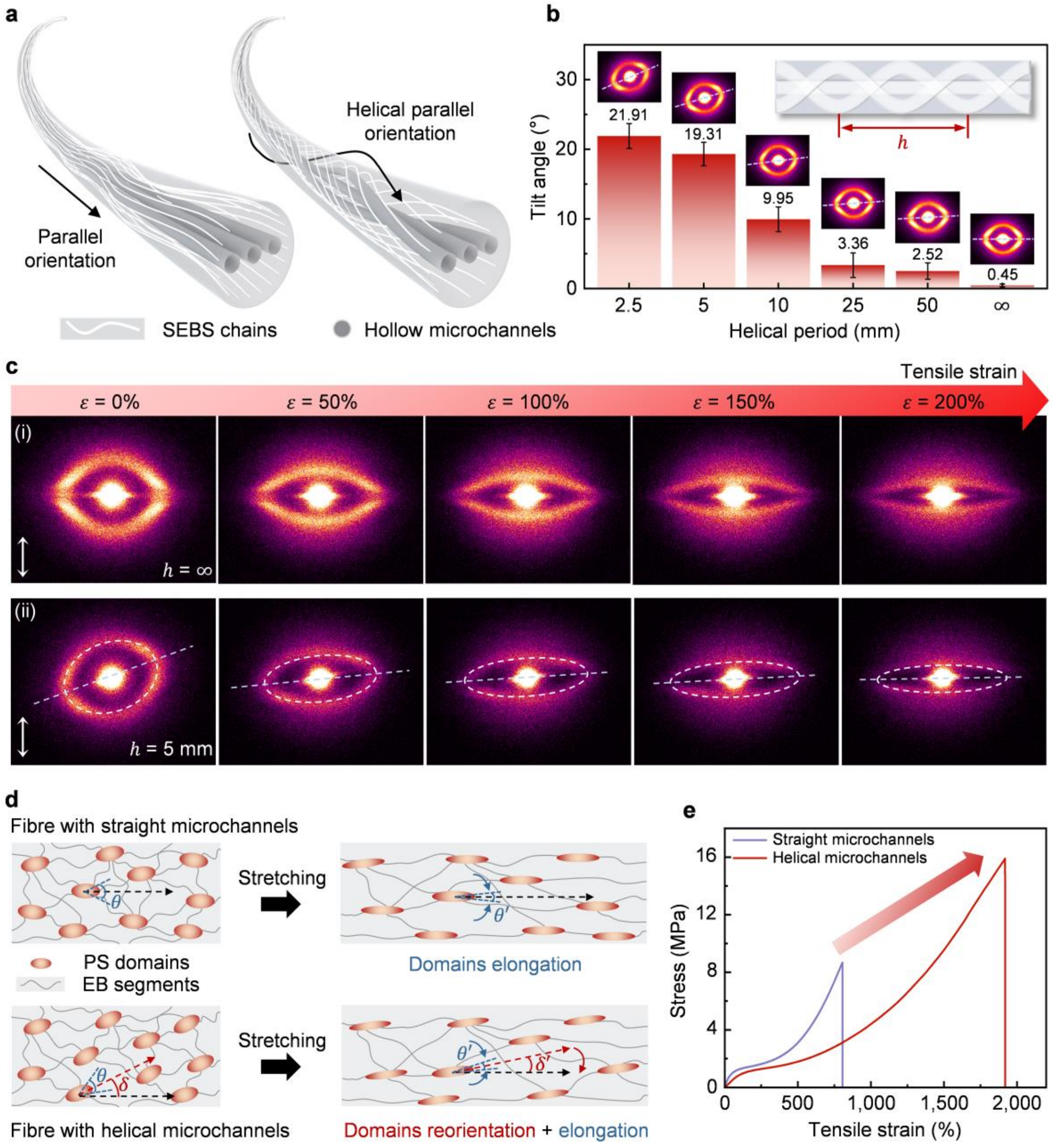

Fig. 2 | Regulation of polymer chain orientation enhances fibre mechanics properties. a, Schematic diagram of polymer chain orientation in fibres with straight and helical microchannels. **b,** Polymer chain tilt angles in tri-channel fibres with different h. The inset shows corresponding 2D SAXS patterns and a schematic of a fibre with helical period of h. **c,** 2D SAXS patterns of the fibre with straight microchannels ($h = \infty$, i) and the fibre with helical microchannels ($h = 5$ mm, ii) under different tensile strains. **d,** Schematic diagram illustrating the evolution of PS domains and polymer chain orientation in fibres with straight and helical microchannels during stretching. **e,** Stress−strain curves for fibres with straight ($h = \infty$, purple) and helical ($h = 5$ mm, red) microchannels.

(2) Comparative mechanical analysis: Fig. 2e and Supplementary Fig. 10a contrast the mechanical performance of fibres featuring helical versus straight microchannels (corresponding to rotational vs. non-rotational drawing). Notably, the fibre with a helical configuration of h = 5 mm shows a >100% increase in elongation at break (rising from 805% to 1,918%) alongside an 83% increase in breaking strength—improvements that surpass the limits of conventional drawing mechanisms.

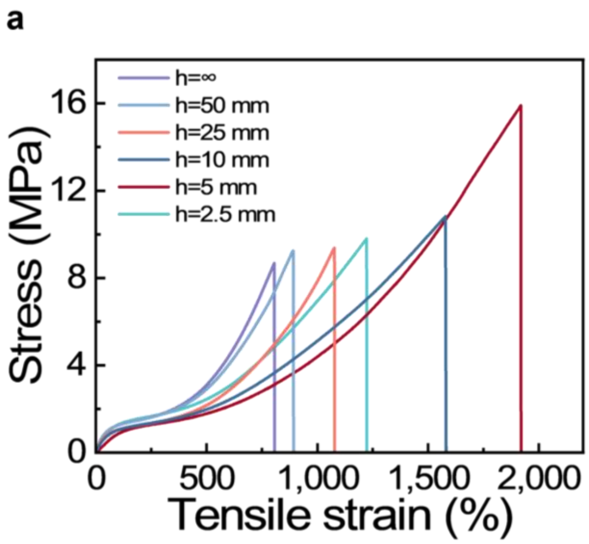

Supplementary Fig. 10 | Mechanical properties and mechanical cycle performance of the tri-channel fibre. a, Stress–strain curves for tri-channel fibres with different h.

(3) Excluding the influence of geometric effects: To isolate the influence of the internal macroscopic channel geometry on the regulation of the fibre's mechanical properties, we fabricated solid SEBS fibre counterparts via both rotational and non-rotational drawing protocols. We systematically compared their mechanical properties against their microchanneled equivalents (Supplementary Fig. 9). **Experimental results reveal that rotationally drawn solid fibre exhibits superior mechanical performance compared to non-rotationally drawn control**, with elongation at break increasing from 804.2% to 2063.2% and breaking strength rising from 9.21 MPa to 16.73 MPa.

Notably, the **non-rotationally drawn solid SEBS fibre** shows similar elongation at break to the **non-rotationally drawn SEBS fibre with straight microchannels**, but the solid structure exhibits slightly higher breaking strength due to the absence of stress concentration caused by internal microchannels. Similarly, the **rotationally drawn solid fibre** demonstrates higher elongation at break and breaking strength than the **rotationally drawn fibre with helical microchannels**. This superiority stems from the inherent structural integrity of the solid fibre, which mitigates potential local collapse or interfacial defects inherent to microchannels during stretching. Consequently, the strengthening and toughening effects of the helically oriented polymer chains are maximized. Furthermore, in the initial strain regime, **non-rotationally drawn fibres** exhibit a higher yield stress, attributable to the alignment of polymer chains parallel to the loading axis. In contrast, the helically oriented chains in **rotationally drawn fibres** lower the energy barrier for deformation onset, resulting in a reduced initial modulus.

Collectively, by eliminating the effect of macroscopic channel geometry, these findings unequivocally confirm that the helical chain orientation induced by rotational thermal drawing is the intrinsic driver of the observed mechanical enhancement.

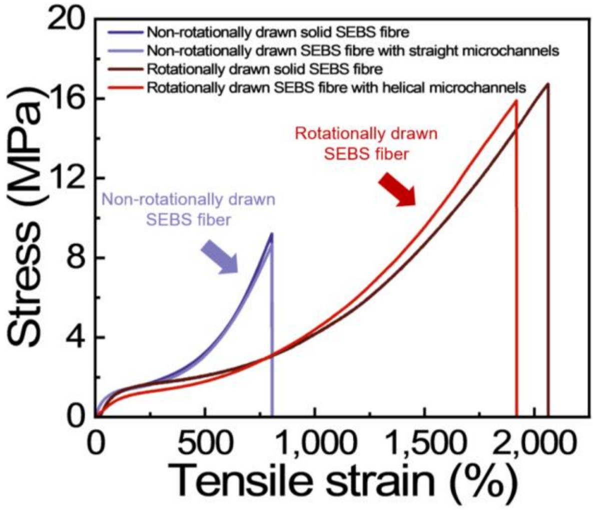

Supplementary Fig. 9 | Comparative mechanical properties of microchanneled fibres and their solid counterparts fabricated via rotational versus non-rotational drawing. The helical period (*h*) of the rotationally drawn fibres is fixed at 5mm.

We have revised and expanded the relevant sections to further elucidate how rotation during thermal drawing regulates polymer chain orientation within the fibre, thereby enhancing its mechanical properties. We trust that these revisions adequately address your concerns and clarify the underlying mechanisms.

[Manuscript, Page 10-11]

As depicted in Fig. 2e, the fibre with helical microchannels exhibits a substantial enhancement in both elongation at break (from 805% to 1,918%) and breaking strength (from 8.68 MPa to 15.91 MPa), relative to its counterpart with straight microchannel. This enhanced mechanical behaviour is primarily attributed to the specific chain orientation regulated by rotational thermal drawing, which strengthens the intermolecular interactions and the material's capacity for energy dissipation during deformation (Supplementary Fig. 9, and see Supplementary Note 3 for details).

[Supplementary Information, Page 9]

To decouple the geometric influence of internal macroscopic channels on mechanical performance, solid SEBS fibre equivalents were fabricated using both rotational and non-rotational drawing protocols. These solid fibres were systematically benchmarked against their microchanneled counterparts to elucidate the contribution of helically oriented polymer chains to mechanical properties (Supplementary Fig. 9). Notably, the rotationally drawn solid fibres exhibited markedly enhanced mechanical performance relative to the non-rotational controls, with elongation at break increasing from 804.2% to 2063.2% and breaking strength rising from 9.21 MPa to 16.73 MPa.

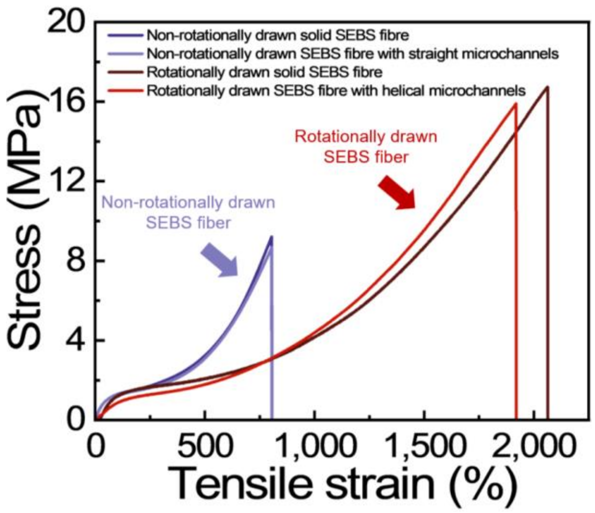

Supplementary Fig. 9 | Comparative mechanical properties of microchanneled fibres and their solid counterparts fabricated via rotational versus non-rotational drawing. The helical period (*h*) of the rotationally drawn fibres is fixed at 5mm.

Additionally, the non-rotationally drawn solid SEBS fibre shows similar elongation at break to the non-rotationally drawn SEBS fibre with straight microchannels, but the solid structure exhibits slightly higher breaking strength due to the absence of stress concentration caused by internal microchannels. Similarly, the rotationally drawn solid fibre demonstrates higher elongation at break and breaking strength than its counterpart with helical microchannels. This enhanced performance stems from the inherent structural integrity of the solid fibre, which circumvents potential local collapse or interfacial defects characteristic of microchannels during stretching. Consequently, the strengthening and toughening effects imparted by the helically oriented polymer chains are maximized. Furthermore, in the initial strain regime, non-rotationally drawn fibres exhibit a higher yield stress, attributable to the alignment of polymer chains parallel to the loading axis. In contrast, the helically oriented chains in rotationally drawn fibres lower the energy barrier for deformation onset, resulting in a reduced initial modulus.

Collectively, by eliminating the influence of macroscopic channel geometry, these findings substantiate that the helical chain orientation induced by rotational thermal

drawing serves as the primary driver of the observed mechanical enhancement.

2. The tri-channel fiber architecture improves robustness and dual-mode sensing, but appears to reflect architectural optimization of existing LM-fiber strategies. The sensing concept and physical mechanisms do not depart substantially from prior works in multi-modal LM fibers.

Response: We thank the reviewer for the insightful comments regarding the conceptual distinction between our tri-channel fibre design and existing LM fibre strategies. While we acknowledge that our sensing mechanisms are grounded in established physical and electrical principles, the primary innovation of this study lies in translating these principles into unprecedented device performance and functional capabilities.

Prior studies utilizing LM fibres have largely been confined to detecting a single strain modality. Those capable of sensing multiple deformation modes often fail to decouple individual strain components in real time under realistic, superimposed loading [*Adv. Funct. Mater.* 2017, **27**, 1605630; *Nat. Commun.* 2021, **12**, 1416; *Sci. Adv.* 2022, **8**, eabo0869; *Adv. Mater.*, 2025, **37**, 2415268; *Nat. Electron.* 2025, **8**, 1072–1081]. This limitation significantly compromises their reliability in dynamic, complex deformation environments.

To bridge this gap, we engineered a novel tri-channel architecture integrated within a single stretchable fibre. This structural innovation is synergized with a custom-developed strain decoupling algorithm, ensuring both high accuracy and computational efficiency in composite strain sensing. Leveraging this design, **we demonstrate—for the first time—the real-time, simultaneous decoupling of tensile and torsional strains over extensive deformation ranges using a single-fibre platform. This capability remains elusive in previously reported multi-modal LM-fibre systems.** Consequently, a key novelty of our work stems from transforming fundamental physical principles into a high-performance device that resolves a critical sensing bottleneck via innovative tri-channel geometry and

fabrication. This represents a pivotal advancement in the field of fibre electronics, paving the way for practical applications in complex motion monitoring and structural health management.

To explicitly articulate this conceptual and functional distinction, we have revised the relevant sections of the manuscript, as detailed below.

[Manuscript, Page 4]

Despite the proliferation of research into multi-modal fibres, the majority of existing stretchable strain sensors prioritize performance optimization under isolated deformation modes[37]. This limitation stems not from a deficiency in fundamental sensing mechanisms, but rather from the inherent challenge of decoupling independent signals from either monolithic materials or simple conductive pathways subjected to complex strain coupling. Consequently, a critical capability gap remains regarding the accurate, real-time monitoring of dynamically superimposed strain states.

[Manuscript, Page 23]

This dual-strain fibre sensor resolves the persistent challenge of real-time decoupling for superimposed strains, holding significant promise for advanced intelligent systems, particularly in applications requiring real-time health monitoring, motion analysis, and high-precision motion capture.

3. The decoupling approach is achieved through additional channels and analytical inference, rather than through intrinsic self-decoupling. While functional, this represents a system-level integration strategy rather than a new sensing mechanism.

Response: We thank the reviewer for this insightful observation, which accurately characterizes the operational principle of our decoupling strategy. We acknowledge that this work does not introduce a novel fundamental sensing mechanism. **Instead, the contribution lies in a system-level integration strategy that synergistically combines**

mechanical regulation of materials and fibre devices, alongside the structural design and engineering implementation of decoupling algorithms. Specifically, we have engineered the internal architecture of a single stretchable fibre to integrate multiple microchannels with distinct geometric configurations. Due to these structural variations, the channels exhibit unique and differential electrical responses when subjected to composite mechanical strains. Leveraging these distinct responses, in conjunction with subsequent analytical processing and decoupling algorithm, allows for the simultaneous detection and decoupling of tensile and torsional strains. In essence, strain decoupling is achieved through the synergy between geometrically distinct conductive microchannels and data post-processing, rather than relying on a single sensing mechanism with intrinsic self-decoupling capabilities. Thus, the decoupling capability arises not from an intrinsic self-decoupling mechanism, but from the coordinated system-level design that integrates material engineering, structural configuration, and signal processing. This integrated strategy is precisely what enables the high sensing fidelity and robust decoupling performance demonstrated in our work.

To clarify this system-level design paradigm and its implications, we have revised the manuscript as follows:

[Manuscript, Page 4]

Despite the proliferation of research into multi-modal fibres, the majority of existing stretchable strain sensors prioritize performance optimization under isolated deformation modes[37]. This limitation stems not from a deficiency in fundamental sensing mechanisms, but rather from the inherent challenge of decoupling independent signals from either monolithic materials or simple conductive pathways subjected to complex strain coupling. Consequently, a critical capability gap remains regarding the accurate, real-time monitoring of dynamically superimposed strain states.

[Manuscript, Page 14-15]

When tensile and torsional strains are applied concurrently, the resistive signal responds exclusively to tensile strain while remaining insensitive to torsion. Conversely, variations in the capacitive signal arise from the synergistic influence of both strain types. Consequently, decoupling these combined loading conditions is unachievable by monitoring either the resistive or capacitive signal in isolation. This necessitates a system-level integration strategy, wherein multiple microchannels are strategically utilized to incorporate distinct sensing mechanisms within a single fibre, thereby enabling strain separation through analytical modeling. Specifically, this involves a sequential analytical approach: first quantifying tensile strain via the resistive signal, and subsequently utilizing this value alongside the capacitive response to decouple and resolve the torsional strain (see Supplementary Note 6 for details). Thus, the synergistic utilization of both electrical sensing mechanisms is essential to not only distinguish individual strains, but also effectively decouple and monitor them under complex combined loading conditions.

Response to Reviewer #1:

Thank you for your thorough and thoughtful responses to my comments. I appreciate the care you have taken to address each point clearly and comprehensively. I have no further comments and believe the manuscript is now suitable for publication.

Response: We sincerely thank Reviewer 1 for their positive evaluation and for recommending our manuscript for publication. We deeply appreciate the time and effort you have dedicated to reviewing our work.

Response to Reviewer #2:

Reviewer #2 has provided comments to the editor only, recommending publication.

Response: We are very grateful to Reviewer 2 for the encouraging feedback and the recommendation for acceptance. Your previous constructive suggestions were invaluable in strengthening our study.

Response to Reviewer #3:

The author has answered my questions. I recommend that this paper be published.

Response: We highly appreciate Reviewer 3 for their thorough review and supportive comments. Thank you for your final endorsement of our research.
